# Sensory neuron–expressed FGF13 controls nociceptive signaling in diabetic neuropathy models

**DOI:** 10.1172/JCI183749

**Published:** 2025-07-15

**Authors:** Aditya K. Singh, Matteo Bernabucci, Nolan M. Dvorak, Zahra Haghighijoo, Jessica Di Re, Nana A. Goode, Feni K. Kadakia, Laura A. Maile, Olumarotimi O. Folorunso, Paul A. Wadsworth, Cynthia M. Tapia, Pingyuan Wang, Jigong Wang, Haiying Chen, Yu Xue, Jully Singh, Kali Hankerd, Isaac J. Gamez, Makenna Kager, Vincent Truong, Patrick Walsh, Stephanie I. Shiers, Nishka Kuttanna, Hanyue Liao, Margherita Marchi, Erika Salvi, Ilaria D’Amato, Daniela D’Amico, Parsa Arman, Catharina G. Faber, Rayaz A. Malik, Marina de Tommaso, Dan Ziegler, Krishna Rajarathnam, Thomas A. Green, Peter M. Grace, Matthew R. Sapio, Michael J. Iadarola, Gregory D. Cuny, Diana S. Chow, Giuseppe Lauria Pinter, Steve Davidson, Dustin P. Green, Jun-Ho La, Jin Mo Chung, Jia Zhou, Theodore J. Price, Elizabeth Salisbury, Subo Yuan, Fernanda Laezza

**Affiliations:** 1 Department of Pharmacology and Toxicology, The University of Texas Medical Branch, Galveston, Texas, USA.; 2 Department of Anesthesiology, University of Cincinnati, College of Medicine, Cincinnati, Ohio, USA.; 3 Department of Neurobiology and; 4 Department of Orthopaedic Surgery and Rehabilitation, The University of Texas Medical Branch, Galveston, Texas, USA.; 5 Anatomic Incorporated, Minneapolis, Minnesota, USA.; 6 Department of Neuroscience, Center for Advanced Pain Studies, The University of Texas at Dallas, Dallas, Texas, USA.; 7 College of Pharmacy, Department of Pharmacological and Pharmaceutical Sciences, University of Houston, Houston, Texas, USA.; 8 Neuroalgology Unit, IRCCS Foundation “Carlo Besta” Neurological Institute, Milan, Italy.; 9 Department of Clinical Genetics, Maastricht University Medical Center, Maastricht, Netherlands.; 10 Institute of Cardiovascular Sciences, Cardiac Centre, Faculty of Medical and Human Sciences, The University of Manchester and NIHR/WellcomeTrust Clinical Research Facility, Manchester, United Kingdom.; 11 Research Division, Weill Cornell Medicine-Qatar, Qatar Foundation, Education City, Doha, Qatar.; 12 Neurophysiopathology Unit, DiBrain Department, Aldo Moro University, Bari, Italy.; 13 Institute for Clinical Diabetology, German Diabetes Center, Leibniz Center for Diabetes Research at Heinrich Heine University, Düsseldorf, Germany.; 14 Department of Biochemistry and Molecular Biology, The University of Texas Medical Branch, Galveston, Texas, USA.; 15 Department of Symptom Research, University of Texas MD Anderson Cancer Center, Houston, Texas, USA.; 16 NIH, Clinical Center, Department of Perioperative Medicine, Bethesda, Maryland, USA.; 17 Department of Clinical Neurosciences, Fondazione IRCCS Istituto Neurologico “Carlo Besta,” Milan, Italy.; 18 Department of Medical Biotechnology and Translational Medicine, University of Milan, Milan, Italy.; 19 Sealy Center for Environmental Health & Medicine, The University of Texas Medical Branch, Galveston, Texas, USA.

**Keywords:** Neuroscience, Public Health, Pain, Sodium channels

## Abstract

Nociception involves complex signaling, yet intrinsic mechanisms bidirectionally regulating this process remain unexplored. Here, we show that the fibroblast growth factor 13 (FGF13)/Nav1.7 protein–protein interaction (PPI) complex bidirectionally modulates nociception, and that the FGF13/Nav1.7 ratio is upregulated in type 2 diabetic neuropathy (T2DN). PW164, an FGF13/Nav1.7 channel C-terminal tail domain (CTD) PPI interface inhibitor, which reduces complex assembly, selectively suppressed Na^+^ currents sensitized by capsaicin-induced activation of TRPV1 channels in human induced pluripotent stem cell–derived (hIPSC-derived) sensory neurons and inhibited mechanical and thermal hyperalgesia in mice. FGF13 silencing mimics PW164 activity in culture and in vivo. Conversely, ZL192, an FGF13 ligand that stabilizes FGF13/Nav1.7 CTD assembly, sensitized Na^+^ currents in hIPSC-derived sensory neurons and exerted pronociceptive behavioral responses in mice. ZL192’s effects were abrogated by FGF13 silencing in culture and in vivo and recapitulated by FGF13 overexpression. In a model of T2DN, PW164 injection reduced mechanical hyperalgesia locally and contralaterally without systemic side effects. In donor-derived dorsal root ganglia neurons, FGF13 and Nav1.7 proteins colocalized, and the FGF13/Nav1.7 protein ratio was upregulated in patients with T2DN. Lastly, we found that SCN9A variant V1831F, associated with painless diabetic neuropathy, abolished PW164-directed modulation of the FGF13/Nav1.7 PPI interface. Thus, FGF13 is a rheostat of nociception and promising therapeutic target for diabetic neuropathy pain.

## Abstract 2

A protein-protein interaction complex in sensory neurons can be tuned to promote or inhibit pain, offering a potential target for diabetic neuropathy treatment.

## Graphical abstract

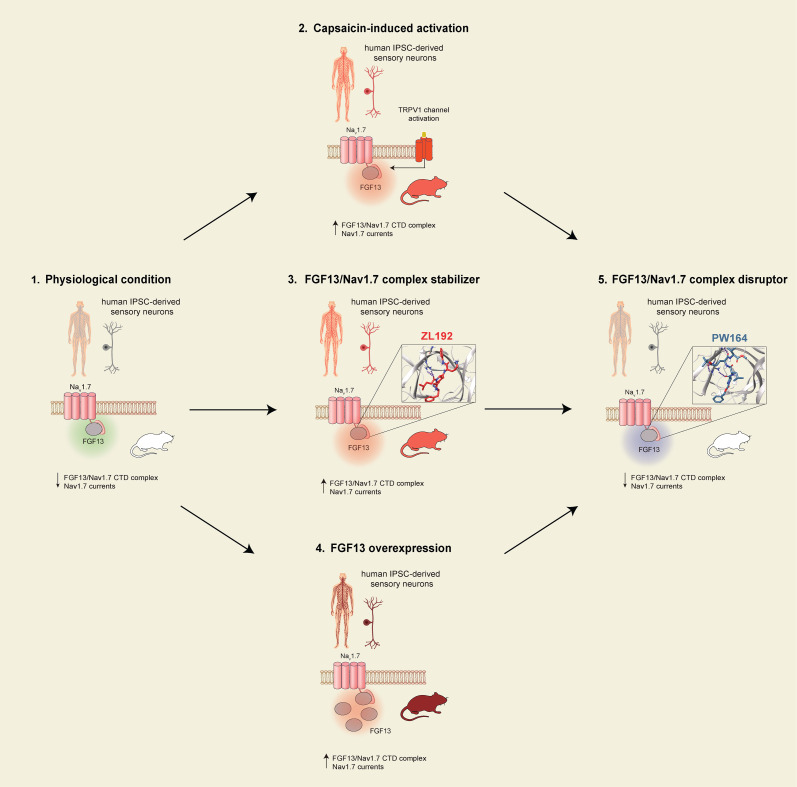

## Introduction

Nociceptive processing begins with activation of peripheral afferent neurons, whose cell bodies are in sensory ganglia such as the dorsal root ganglia (DRG) (1, 2). These neurons maintain defensive homeostasis in response to harmful stimuli, a process encoded by their intrinsic firing and integrated in the spinal cord (2). Disruption of this homeostatic process can cause sustained sensory neuron firing or desensitization, leading to chronic hyperalgesia or hypoalgesia, respectively. Harmful stimuli-induced membrane depolarization and firing of DRG neurons requires activation of the voltage-gated Na^+^ (Nav) channels (Nav1.3, 1.6–1.9) (3) that are central to nociceptive signaling. Milestone gain- and loss-of-function studies and clinical reports demonstrate that the Nav1.7 channel is essential in DRG neuronal firing (4) and important in pain disorders (5, 6). Gain- and loss-of-function mutations in the human Nav1.7 gene cause paroxysmal pain or congenital pain insensitivity (CIP), respectively (7–10). However, despite Nav1.7 gene conservation between rodents and humans (11), decades of drug development targeting Nav1.7 have yielded no antipain medications. The failure, attributed to discrepancies in Nav1.7 function between rodent models and humans (12), warrants exploring novel mechanisms for fine tuning Nav1.7 function and nociception.

The pore-forming α subunit is necessary for Nav1.7 functionality, but auxiliary proteins that bind to the α subunit intracellular C-terminal tail domain (CTD) are essential for full physiological function (13). Among these auxiliary proteins, fibroblast growth factor 13 (FGF13) is a critical modulator of Nav1.7-mediated Na^+^ currents and excitability of DRG neurons in pain-related signals (14–16). Crucially, conditional FGF13 gene deletion attenuates DRG firing and behavioral responses to heat-induced hyperalgesia through a mechanism blocked by a competing peptide mimicking the Nav1.7 CTD (14). Whether FGF13 can be pharmacologically modulated and targeted for therapeutic development in chronic neuropathies remains unclear.

Combining structure-guided drug design with in vitro and in vivo gene silencing studies, we show that 2 recently identified FGF13 ligands confer opposing effects on Nav1.7 currents and nociceptive behavior through bidirectional modulation of Na^+^ currents in rodent- and hIPSC-derived nociceptors, pharmacological effects that are mimicked by genetic manipulation of FGF13 in vitro and in vivo. Compound PW164, which inhibits FGF13/Nav1.7 CTD complex formation, effectively reduces mechanical hyperalgesia in a high-fat diet, type 2 diabetic neuropathy (T2DN) mouse model. Notably, the ratio of FGF13/Nav1.7 mean fluorescent intensity is upregulated in donor-derived DRG neurons from patients with T2DN, suggesting increased complex formation, and a rare genetic variant associated with painless diabetic neuropathy is a predicted hot spot at the FGF13/Nav1.7 complex. These studies reveal that FGF13 is a conserved target with potential for precision treatment of diabetic neuropathy.

## Results

### Structure-guided and in-cell screenings identify FGF13/Nav1.7 complex inhibitors.

**Figure 1:**
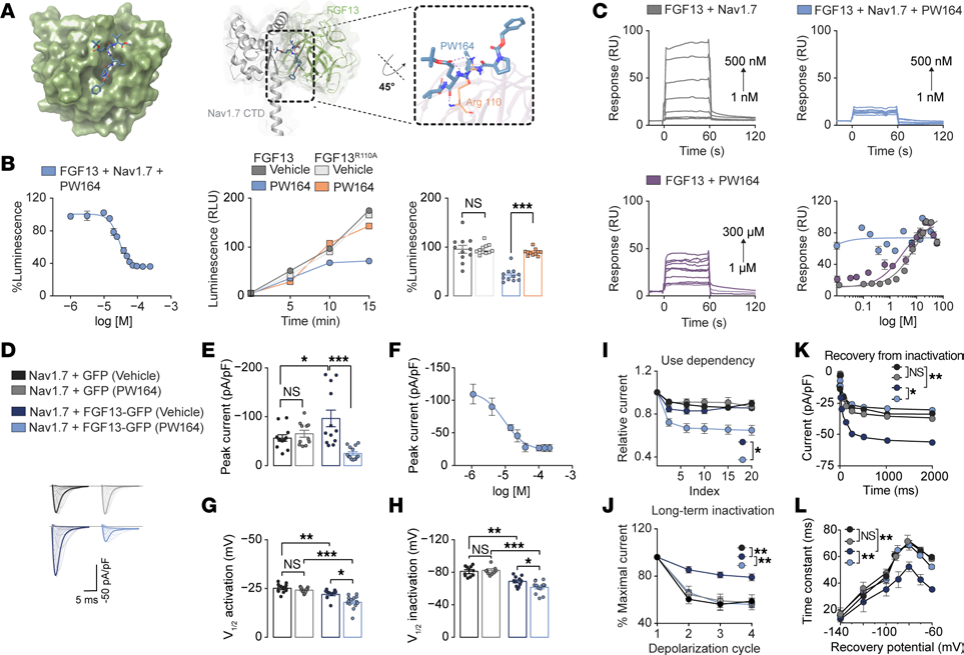
PW164 Inhibition of FGF13/Nav1.7 CTD complex formation selectively modulates Nav1.7 currents. (**A**) PW164 docking on FGF13 surface and docked pose overlay with Nav1.7-CTD; H-bond in purple. (**B**) Percentage luminescence as function of PW164 log_10_ concentration in LCA produced by assembly of CLuc-FGF13/CD4-Nav1.7-CTD-NLuc complex or CLuc-FGF13^R110A^/CD4-Nav1.7-CTD-NLuc with vehicle or PW164 (20 μM). (**C**) Representative SPR sensograms and SSI saturation curves for respective groups. (**D**) Representative traces of *I*_Na_ recorded from HEK-Nav1.7 cells of indicated groups in response to depolarizing voltage steps. (**E** and **F**) Bar graph of peak *I*_Na_ density at voltage step –10 mV and dose response curve (IC_50_ = 6.74 ± 0.5 μM) (*n* = 11–14 cells/group). Scale bar 5 ms, 100 pA/pF. (**G** and **H**) V_½_ of voltage-dependence of activation and steady state inactivation (*n* = 9–17 cells/group). (**I** and **J**) *I*_Na_ amplitudes as a function of time (test pulse number referred to as index or depolarization cycle) in response to trains of variable depolarization protocols denoting use dependency (**I**) or long-term inactivation (**J**) of Nav1.7 (**K** and **L**) Time course (top) and time constants (bottom) of Nav1.7 repriming (recovery from inactivation) shown for indicated groups. Bottom panel denotes *I*_Na_ amplitudes in response to depolarizing pulses to allow channels entering long-term inactivation (*n* = 8–17 cells/group). Data are mean ± SEM; *P < 0.05, **P < 0.01, *** *P* < 0.001, 1-way ANOVA with post hoc Tukey’s multiple comparisons test. Student *t* test compared time constants in panel **L**.

FGF13 belongs to the fibroblast growth factor homologous factors (FHFs) group of intracellular FGF proteins (FGF11–14) (17, 18). While there is evidence of interaction with Nav channels for several iFGF/Nav channel pairs, these complexes are structurally divergent enough to enable selective modulation of different Nav channel isoforms (19, 20). To identify FGF13 ligands capable of tuning the FGF13/Nav1.7 complex, we built a FGF13/Nav1.7 complex homology model based on available structures (20) (Supplemental Figure 1; supplemental material available online with this article; https://doi.org/10.1172/JCI183749DS1) and screened in silico a collection of rationally designed pharmacophores (PW-series) derived from a 4 amino–acid peptide sequence of the FGF1-β12 strand. These were subsequently converted into compounds with improved drug-like properties by adding diverse substituents to the C- and N-termini of the tetrapeptide scaffold (21). In silico screening was run in parallel with split-luciferase complementation assays (LCA) (22), previously utilized to reconstitute iFGFs and Nav channel CTD complexes (23–26) and implemented here to reconstitute the complex formed by the Nav1.7 CTD (CD4-Nav1.7-CTD-NLuc) and FGF13 (CLuc-FGF13) in cells. Unless otherwise noted, FGF13 plasmids used in this study refer to the 1b (U) isoform, due to previous implications in pain signaling (14). LCA combined with in silico screening revealed compound PW164 as a hit (Figure 1A). Hierarchical clustering combining in silico screening parameters (docking score, H-bond coefficient, and lipophilic terms derived from Glide properties), and one concentration of compound tested with the LCA (Supplemental Figure 2, A–D), were used for hit selection. Ligand binding modes of PW164 on the FGF13/Nav1.7 interface showed 2 major stabilizing interactions via H-bonds with Arg110 (Figure 1A). The 2D interaction map revealed hydrophobic interactions between the PW164 benzyl group and FGF13 residues Val153, Leu195, Pro196, Pro198, and Val201 (Supplemental Figure 2E). In silico predictions using Molinspiration and admetSAR revealed that PW164 has a Caco-2 permeability of 84%, surpassing that of morphine (75%) and the FDA-approved ruxolitinib (27) (73%), further supporting PW164’s potential utility. In the LCA, PW164 displayed robust inhibition of FGF13/Nav1.7 complex assembly with a dose-dependent luminescence reduction (Figure 1B; IC_50_ value of 29 ± 5 μM). Consistent with in-silico predictions, a mutation from Arg to Ala at FGF13^R110^, a hot spot at the iFGF/Nav interface (28, 29), specifically prevented PW164 binding to the FGF13^R110A^/Nav1.7 complex without affecting complex formation in control conditions (Figure 1B). Next, we employed surface plasmon resonance (SPR) as a cell-free orthogonal validation, and FGF13 formed a high-affinity complex with the Nav1.7 CTD (Figure 1C, grey; K_D_=75 ± 3 nM). Remarkably, PW164 displaced FGF13 binding to Nav1.7 CTD resulting in a K_D_ value below reliable determination (Figure 1C, blue). To determine if these effects were from direct binding to FGF13 or the CTD of Nav1.7, the K_D_ of PW164 towards each protein was determined separately. PW164 displayed appreciable binding for FGF13 (Figure 1C, purple; K_D_ of 21 ± 3 μM) but showed no interaction with Nav1.7 CTD (K_D_ not measurable). These in vitro data, consistent with LCA and molecular modeling studies, substantiate that PW164 directly binds to FGF13, disrupting FGF13/Nav1.7 complex assembly. To assess functional activity of PW164 on Nav1.7 currents, we performed whole-cell patch-clamp electrophysiology in HEK293 cells expressing human Nav1.7 and either FGF13-GFP (FGF13U or FGF13-1b) or GFP. Transfection of GFP alone was the control. As previously reported, coexpression of FGF13 significantly increased peak transient Na^+^ current density (I_Na_) (Figure 1, D and F, dark blue) and caused depolarizing shifts in activation (Figure 1G) and steady-state inactivation (SSI) (Figure 1H). Additionally, while FGF13 alone did not affect Nav1.7 use dependency (Figure 1I), it prevented Nav1.7 long-term inactivation (LTI; Figure 1J) and accelerated recovery from fast inactivation known as channel repriming (FGF13-GFP + vehicle: τ=53 ms compared to Nav1.7-GFP+vehicle: τ=70 ms; Figure 1, K and L). Consistent with an effect requiring binding to FGF13, PW164 was functionally active toward Nav1.7 currents only in cells coexpressing FGF13-GFP (Figure 1, D–L, light blue). However, PW614 exhibited mixed functional responses, indicating antagonism, agonism, or additional gain-of-function effects. PW164 reversed FGF13-induced increase in I_Na_ density dose dependently, with an IC_50_ of 6.74 ± 0.5 μM (Figure 1F) and induced LTI (Figure 1J) while decelerating Nav1.7 repriming (Figure 1, K and L). However, PW164 exacerbated FGF13’s effects on the voltage-dependence (V_1/2_) of activation (Figure 1G) and SSI (Figure 1H) and enhanced use dependency, preventing channel reopening during repetitive stimulation (Figure 1I). Additional representative traces are presented in Supplemental Figure 3 and results are summarized in Supplemental Table 1. Notably, PW164 was inactive on Nav1.7 cells coexpressing FGF13-1a-GFP (FGF13S) (Supplemental Table 2), on HEK293 cells stably expressing Nav1.6 alone or coexpressing FGF13 (Supplemental Figure 4 and Supplemental Table 3), or in Nav1.5-expressing HEK293 cells alone or with FGF13-1b or FGF13-1a (Supplemental Table 4). These findings demonstrate that: (a) the FGF13/Nav1.7 CTD protein complex is a suitable target for developing protein-protein interaction (PPI) modulators and (b) ligands targeting the FGF13/Nav1.7 complex provide structural specificity for selective pharmacological modulation of Na^+^ currents.

### PW164 inhibition of the FGF13/Nav1.7 protein complex selectively suppresses capsaicin-induced signaling in hIPSC-derived sensory neurons.

The Nav1.7 channel is widely expressed in sensory neurons and its hyperactivity is characteristic of peripheral inflammatory pain (30–32). Nav1.7 interactions with FGF13, also abundant in sensory neurons (16), mediate noxious stimuli–induced cellular signaling (14), a process typically activated by the transient receptor potential cation channel subfamily V member 1 (TRPV1) (33, 34). Previous reports demonstrate that heat-induced potentiation of DRG excitability is dependent on formation of the FGF13/Nav1.7 complex (14), which may maintain or potentiate signals initiated by TRPV1 activation, although the effects on Nav channel activity have not been fully elucidated. Thus, we sought to assess the effects of TRPV1 activation on Na^+^ currents in sensory neurons and if the observed effects are blocked by PW164. To test this, we utilized commercially available human induced pluripotent stem cell–derived (hIPSC-derived) sensory neurons trademarked as RealDRG (Anatomic Incorporated) (35–37). These neurons express *TRPV1* mRNA and the encoded protein was detected (Supplemental Figure 5). Through immunocytochemistry with previously validated anti-Nav1.7 (38) and anti-FGF13 (39) antibodies, we also confirmed the presence of FGF13 and Nav1.7 (Supplemental Figure 6). Furthermore, we showed increased colocalization of FGF13 and Nav1.7 proteins after capsaicin treatment that was reduced by PW164. Interestingly, we found that PW164 increased the intracellular pool of Nav1.7 channels, suggesting that capsaicin enhances FGF13/Nav1.7 channel complex trafficking to the plasma membrane, while PW164 promotes complex dissociation. This was evidenced by increased fluorescence intensity of the cytoplasmic intracellular pool of Nav1.7 channels (approximately 8 μm away from the inner leaflet of the plasma membrane), presumably internalized and endocytosed away from the plasma membrane (Supplemental Figure 6) (40).

**Figure 2:**
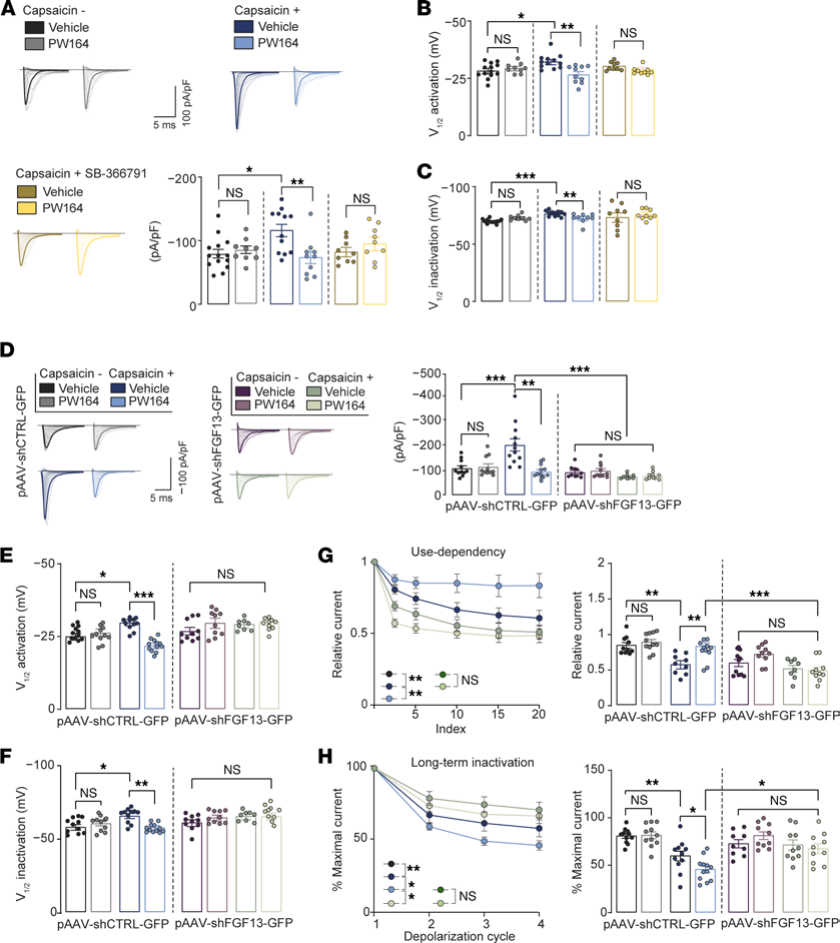
FGF13 chemical inhibition or genetic silencing selectively suppress capsaicin-induced signaling in hIPSC-derived sensory neurons. (**A**) Representative traces of *I*_Na_ in human RealDRG with indicated groups (*n* = 9–14 cells/group) and corresponding bar plots of peak *I*_Na_ density (**B**) V_½_ of voltage-dependence of activation. (**C**) V_½_ of steady state inactivation (right, *n* = 9–12 cells/group). (**D**) Representative traces of *I*_Na_ recorded in human RealDRG transfected with pAAV-CTRL-GFP or pAAV-shFGF13-GFP or from color-coded groups (*n* = 7–12 cells/group). (**E**) V_½_ of the voltage-dependence of activation. (**F**) V_½_ of voltage-dependent steady state inactivation (*n* = 7–12 cells/group). (**G**) Line and bar plots of use-dependent cumulative inactivation recorded at 10 Hz. (**H**) Percentage maximal current as a function of depolarization cycle denoting channel long-term inactivation (bottom, index; *n* = 8–10 cells/group). Data are mean ± SEM, *P < 0.05, **P < 0.01, *** *P* < 0.001, 1-way ANOVA with post hoc Tukey’s multiple comparisons test.

Next, we conducted whole-cell patch-clamp electrophysiological recordings in hIPSC-derived sensory neurons under three conditions: unstimulated and treated with 0.07% ethanol (capsaicin vehicle), treated with capsaicin (100 nM, 10 minutes), and treated with capsaicin plus SB-366791 (10 μM), a selective TRPV1 inhibitor. Each group was exposed to either vehicle (0.01% DMSO) or PW164 (50 μM), with all recordings performed in the presence of the Nav1.8 blocker A-803467 (300 nM) (41). The experimental groups are color coded in Figure 2A. Capsaicin caused potentiation of *I*_Na_ (Figure 2A) and induced hyperpolarizing shifts in the V_1/2_ of activation (Figure 2B) and SSI (Figure 2C). Notably, PW164 (50 μM, 30–60 minutes) reversed capsaicin-induced phenotypes restoring Na^+^ currents to a nonstimulated state (Figure 2, A–C), except for LTI, where PW164 exacerbated the capsaicin phenotype (Figure 2H). PW164 showed no significant alterations of Na^+^ currents in neurons in unstimulated control conditions or treated with either the TRPV1 antagonist SB-366791 (Figure 2, A–C) or the Nav1.7 blocker ProTx II (10 nM; Supplemental Figure 7 and Supplemental Table 5) indicating PW164 is a selective modulator of Nav channels sensitized by capsaicin-induced activation of TRPV1 channels. Next, we investigated whether silencing FGF13 could replicate PW164’s effects and prevent additional activity of the compound. hIPSC-derived sensory neurons were transfected with a previously validated pAAV-shFGF13-GFP construct (42) (Supplemental Figure 8). Whole-cell patch recordings revealed that, while neurons expressing pAAV-shCTRL-GFP exhibited the same sensitivity to capsaicin and/or PW164 as untransfected neurons (Figure 2, A–C), neurons expressing pAAV-shFGF13-GFP were resistant to changes induced by capsaicin alone or in combination with PW164 (Figure 2, D–H). Neither PW164 nor FGF13 silencing affected Na+ currents in neurons transfected with pAAV-shCTRL-GFP or pAAV-shFGF13-GFP, whether unstimulated or treated with capsaicin in the presence of ProTx II (Supplemental Figure 9 and Supplemental Table 6). Thus, PW164 selectively modulated FGF13-dependent Na^+^ current sensitization via TRPV1, with FGF13 silencing mimicking and validating PW164’s effect.

### PW164 is an FGF13-dependent analgesic that preserves normal sensory function.

**Figure 3:**
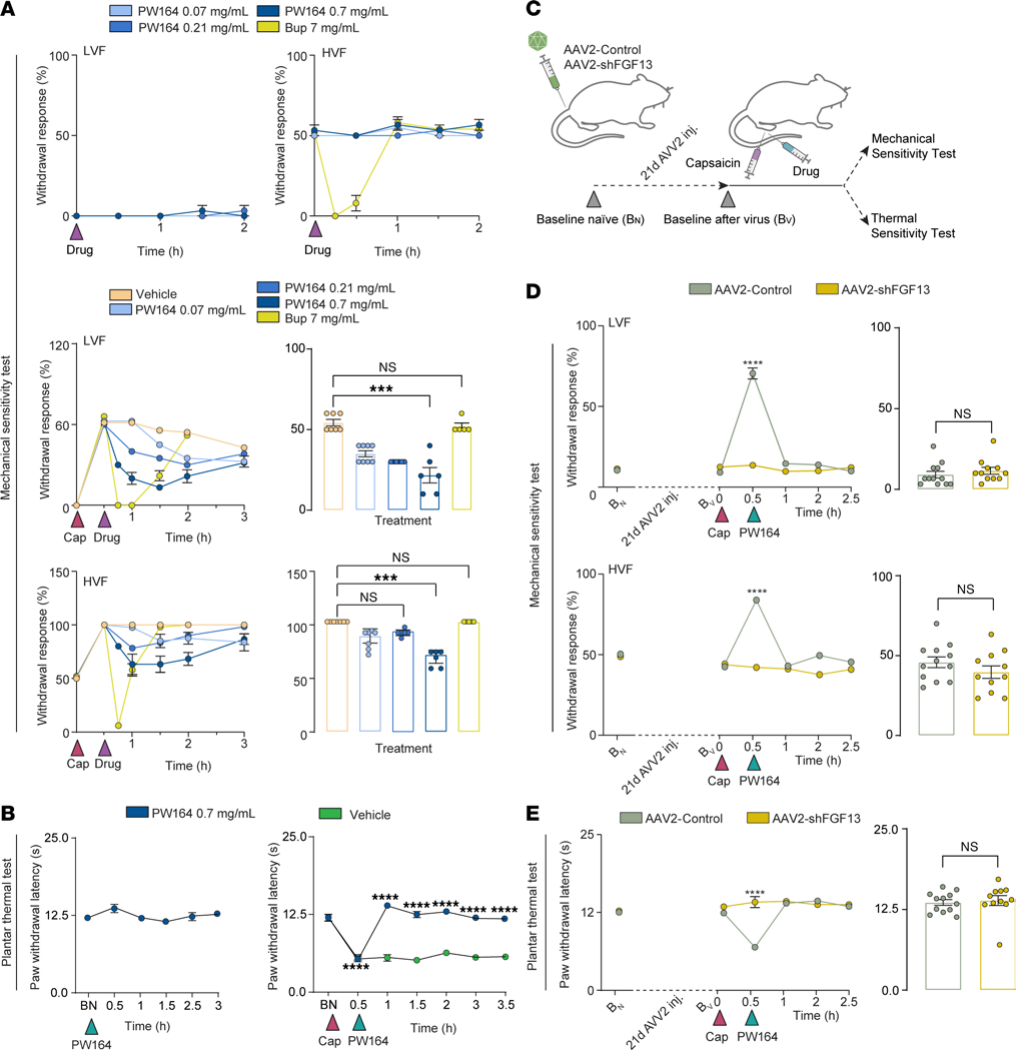
Inhibition of FGF13 exerts antinociceptive effects and mimics in vivo FGF13 knockdown. (**A**) Paw withdrawal response at low (LVF) and high intensity (HVF) von Frey filament stimulation after intraplantar PW164 injection or bupivacaine (bup) (*n* = 3–4 mice/group). Paw withdrawal response to LVF and HVF stimulation with bup or PW164 30 minutes after capsaicin (cap); corresponding plots at 2 hours after capsaicin injection. (**B**) Time course of thermal radiant heat (Hargreaves) test showing paw withdrawal response (latency) in mice after intraplantar PW164 injection (0.7 mg/mL) alone or 30 minutes after capsaicin; *n* = 12/group. Green represents vehicle-only injection with capsaicin. (**C**) Experimental design showing lumbar intrathecal injection of viral particles and tests 21 days after viral injection (BV). Capsaicin and subsequent PW164 (0.7 mg/mL) injected locally in the same paw area 30 minutes apart. (**D**) Paw withdrawal response to LVF and HVF stimulation before and after local capsaicin injection followed by PW164 and corresponding bar graphs at 2 hours after PW164. (**E**) Plots corresponding to the same groups as **D** in response to thermal sensitivity test. Data are mean ± SEM; *P < 0.05, **P < 0.01, ***P < 0.001, ****P < 0.0001; 1-way ANOVA with post hoc Tukey’s or 2-way ANOVA with Šidák’s multiple comparisons test.

*FGF13* is a neuronal gene ubiquitously transcribed in DRG neuronal subtypes (43–46). To investigate neuronal subtype enrichment of the FGF13 transcript, we examined a gene panel including *FGF13*, *SCN9A,* and several marker genes in mouse single-cell transcriptomic data (Supplemental Figure 10) (43–46). *FGF13* and *SCN9A* showed similar enrichment, with highest levels of expression in 2 populations. Critically, putative silent nociceptors marked by somatostatin (*SST*), and the nicotinic cholinergic receptor α-3 subunit (*CHRNA3*) contribute to development of inflammatory mechanical hyperalgesia (47). *FGF13* is expressed at comparatively lower levels in large diameter proprioceptors and low-threshold mechanoreceptors (LTMRs), which express high levels of osteopontin (*SPP1*). This indicates robust coexpression of *FGF13* and *SCN9A* in subpopulations of DRG neurons relevant to nociceptive signaling. We next determined if PW164 modulation of the FGF13/Nav1.7 complex could suppress nociceptive behaviors in WT mice. We tested whether intraplantar (i.pl.) injection (5 μL) of PW164 altered hind paw withdrawal in response to low or high intensity von Frey filament (LVF or HVF, respectively) mechanical stimulation (Figure 3A). Unlike the anesthetic bupivacaine (7 mg/mL, 5 μL local injection), PW164 did not affect paw withdrawal at the doses tested (Figure 3A). Neither did PW164 affect thermal hindpaw withdrawal thresholds measured as latency responses in the radiant heat test (Figure 3B). Thus, PW164 does not affect normal mechanical or thermal sensitivity. Next, we evaluated PW164’s effect on mechanical and thermal hindpaw sensitivity in capsaicin-induced nociceptive hypersensitivity models. Thirty minutes post-capsaicin (0.1%, 10 μL; 1 μg/10μL; i.pl.), mice exhibited increased mechanical and thermal sensitivity reflecting sensitization of peripheral nociceptors (Figure 3, A and B). This was inhibited by the administration of PW164 at the capsaicin injection site (Figure 3, A and B); a dose-dependent effect observed down to 0.21 mg/mL. Next, we performed in vivo target validation in WT mice that received an intrathecal (i.t.) injection in L5 of AAV2-shFGF13 (6 μL, viral titers 1 × 10^12^ vector genomes (vg)/mL); a control group received an equal number of AAV2-shCTRL particles (Figure 3C). Knockdown efficacy of AAV2-shFGF13 is shown in Supplemental Figure 11A. Baseline mechanical and thermal responses were done before and three weeks after viral particle delivery and showed no altered sensitivity to harmful mechanical and heat stimulations (Figure 3, E). Mice injected with AAV2-shFGF13 particles were resistant to capsaicin-induced mechanical and thermal hypersensitivity and subsequent hind paw injection of high dose PW164 (5 μL, 0.7mg/mL) did not further modify mechanical or thermal sensitivity (Figure 3, D–E). Separate animal groups—naive mice and mice injected with AAV2-shCTRL viral particles—developed local mechanical and thermal hypersensitivity after capsaicin that was reversed by i.pl. PW164 injection (0.7 mg/mL), confirming its analgesic effect for at least 2 hours. These results indicate that: (a) PW164 selectively inhibits capsaicin-induced hyperalgesia, preserving normal sensory function; (b) in vivo FGF13 gene silencing mimics the antinociceptive effect of PW164; (c) PW164 pharmacological activity is dependent on FGF13 expression; and (d) FGF13 is necessary for mechanical and thermal nociception.

### ZL192 shares binding properties with PW164 but is a positive modulator of the FGF13/Nav1.7 complex.

**Figure 4:**
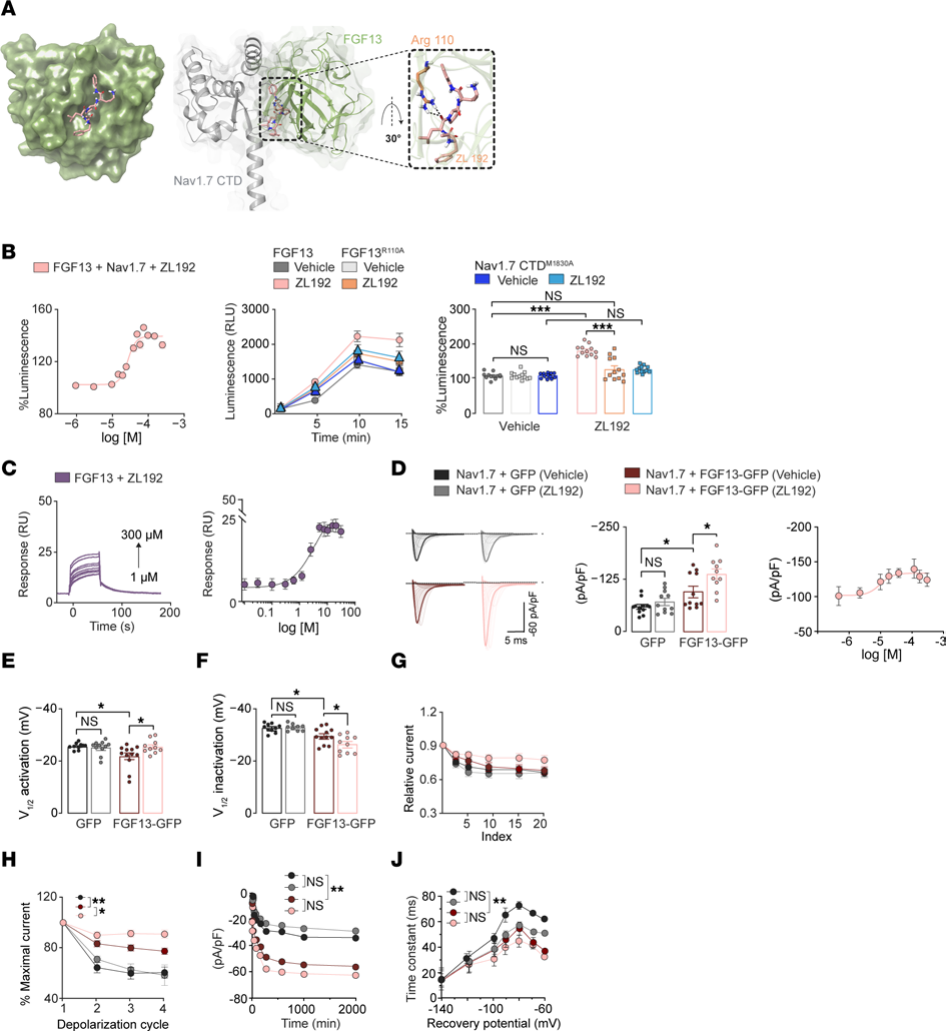
FGF13 activator ZL192 increases FGF13/Nav1.7 CTD complex formation and potentiates Nav1.7 currents. (**A**) Docking of FGF13 positive modulator ZL192 on FGF13 surface and docked pose overlay with Nav1.7-CTD; H-bond is in purple. (**B**) Left, percentage luminescence as a function of log_10_ concentration of ZL192 in LCA produced by assembly of CLuc-FGF13/CD4-Nav1.7-CTD-NLuc. Center, percentage luminescence. Right, corresponding bar graph produced by assembly of CLuc-FGF13^R110A^/CD4-Nav1.7-CTD-NLuc or the CLuc-FGF13/CD4-Nav1.7^M1830A^-NLuc complex with either vehicle (0.5%) or ZL192 (50 μM). (**C**) Representative SPR sensograms and SSI saturation curves for respective groups. (**D**) Representative traces of *I*_Na_ recorded from HEK-Nav1.7 cells of indicated groups in response to depolarizing voltage steps, and bar graph of peak *I*_Na_ density at voltage step –10 mV (*n* = 11–12 cells/group; dose response EC_50_ = 8.5 ± 0.9 μM). (**E** and **F**) Bar graph of voltage dependence of activation and SSI inactivation for indicated groups (*n* = 11–12 cells/group). (**G**) Use dependency showing *I*_Na_ amplitudes as a function of time (test pulse number referred to as index) in response to a train of depolarizations presented at a frequency of 10 Hz. (**H**) *I*_Na_ amplitudes in response to depolarizing pulses representing LTI (*n* = 11–12 cells/group). (**I** and **J**) Time course (**I**) and time constants (**J**) of repriming (recovery from fast inactivation) of Nav1.7 channels in indicated groups. Data are mean ± SEM; *P < 0.05, **P < 0.01, ***P < 0.001, 1-way ANOVA with post hoc Tukey’s multiple comparisons test. Student *t* test compared time constants among groups in **I** and** J**.

We next tested if a structurally diverse compound with opposite effects on FGF13/Nav1.7 complex formation could potentiate Nav1.7 to be pronociceptive. We screened FGF13 ligands that exerted opposite modulation of FGF13/Nav1.7 complex assembly with increased luminescence in the LCA. Because no PW compound exhibited a robust agonistic effect on in the LCA (Supplemental Figure 2), we chose an FLPK sequence-derived library of pharmacophores (the ZL series) (26), a segment adjacent to PLEV at the FGF13 β12 strand (20). Hits for the ZL series were selected via a screening approach combining VS and 50 μM LCA (Supplemental Figure 12), which identified compound ZL192. Docking between ZL192 and Nav1.7 CTD predicted π-π interactions with Arg110, an H-bond with Met1830, and salt bridges with Glu1878 (Figure 4A and Supplemental Figure 12). Thus, while differences in docking positions were found in ZL192 versus PW164, Arg110 was a hotspot mediating energetically favored ligand-protein interactions. In the LCA dose-response, ZL192 increased FGF13/Nav1.7 complex assembly with an EC_50_ = 28 ± 5 μM (Figure 4B), and, like PW164 (Figure 1B), ZL192 had no agonist activity against the FGF13^R110A^/Nav1.7 complex (Figure 4B). Docking predicted that ZL192 binds to both FGF13 at Arg110 and Nav1.7 CTD at M1830, supporting a bridging mechanism where the compound simultaneously interacts with both partners to stabilize the complex. Thus, we introduced an Ala mutation at M1830 and repeated the LCA. ZL192 showed no agonist activity against the FGF13/Nav1.7^M1830A^ complex (Figure 4B), supporting the bridging mechanism hypothesis. Additionally, ZL192 has a predicted Caco-2 permeability of 72%, similar to morphine (75%) and the FDA-approved kinase inhibitor ruxolitinib (27) (73%), further supporting ZL192’s potential utility as a tool compound. Using SPR, we confirmed ZL192 binding to FGF13 (Figure 4C; K_D_ = 4 μM) but could not test direct binding to Nav1.7 because the SPR CTD fragment lacked M1830. We then tested ZL192 functional activity in HEK-Nav1.7 cells, because upon stabilizing the FGF13/Nav1.7 complex, ZL192 should potentiate FGF13 modulation of Nav1.7 currents. As expected, ZL192 exerted regulatory effects on Nav1.7 currents only in HEK-Nav1.7 cells coexpressing FGF13, exacerbating the phenotypes conferred by FGF13 on I_Na_ (EC_50_ = 8.5 ± 0.9 μM; Figure 4D), SSI (Figure 4F), and LTI (Figure 4H and Supplemental Table 7). In contrast, ZL192 inhibited the FGF13-induced depolarizing shift on V_1/2_ of activation (Figure 4E) with no effects on use dependence (cumulative inactivation; Figure 4G and Supplemental Table 4) or recovery from fast inactivation (FGF13-GFP + ZL192: τ = 50 ms compared with FGF13-GFP + vehicle: τ = 52 ms; Figure 4, I and J). Additional representative traces are in Supplemental Figure 13. Like PW164, ZL192 exhibited mixed effects on Nav1.7 currents, consistent with either FGF13 agonism with respect to I_Na_, LTI, and V_1/2_ of SSI, and antagonism or lack of regulation with respect to kinetics of activation, cumulative inactivation, and recovery from fast inactivation. These results indicate that: (a) FGF13 ligands that enhance FGF13/Nav1.7 CTD interactions are sufficient to potentiate Nav1.7 currents and mimic FGF13’s regulatory activity on Nav1.7 currents (except for V_1/2_ of activation) and (b) FGF13/Nav1.7 complex assembly can be bidirectionally modulated by structurally diverse FGF13 ligands with opposite effects on Nav1.7 currents.

### ZL192 and FGF13 overexpression sensitize Na^+^ currents in hIPSC-derived sensory neurons and are pronociceptive.

**Figure 5:**
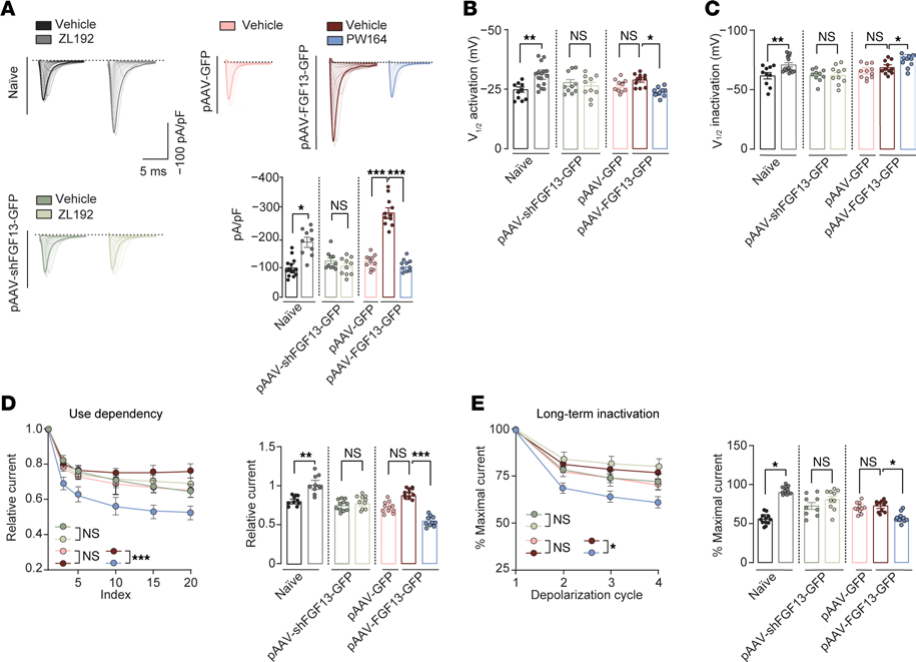
ZL192 and FGF13 overexpression are sufficient to potentiate Na^+^ currents in hIPSC-derived sensory neurons without triggering stimuli. (**A**) Representative traces of *I*_Na_ and peak *I*_Na_ density recorded from human RealDRG neurons in the color-coded groups (*n* = 7–16 cells/group). (**B**) Bar graphs represent V_½_ of activation and (**C**) SSI (right, *n* = 7–12 cells/group). (**D**) Line (left) and bar plots (right) representing use-dependent cumulative inactivation recorded at 10 Hz (left). (**E**) Line (left) and bar plots of long-term inactivation represented as percentage maximal current as a function of the depolarization cycle (right; *n* = 7–16 cells/group). Data are mean ± SEM, *P < 0.05, **P < 0.01, ***P < 0.001, ****P < 0.0001. One-way ANOVA with post hoc Tukey’s multiple comparisons test.

**Figure 6:**
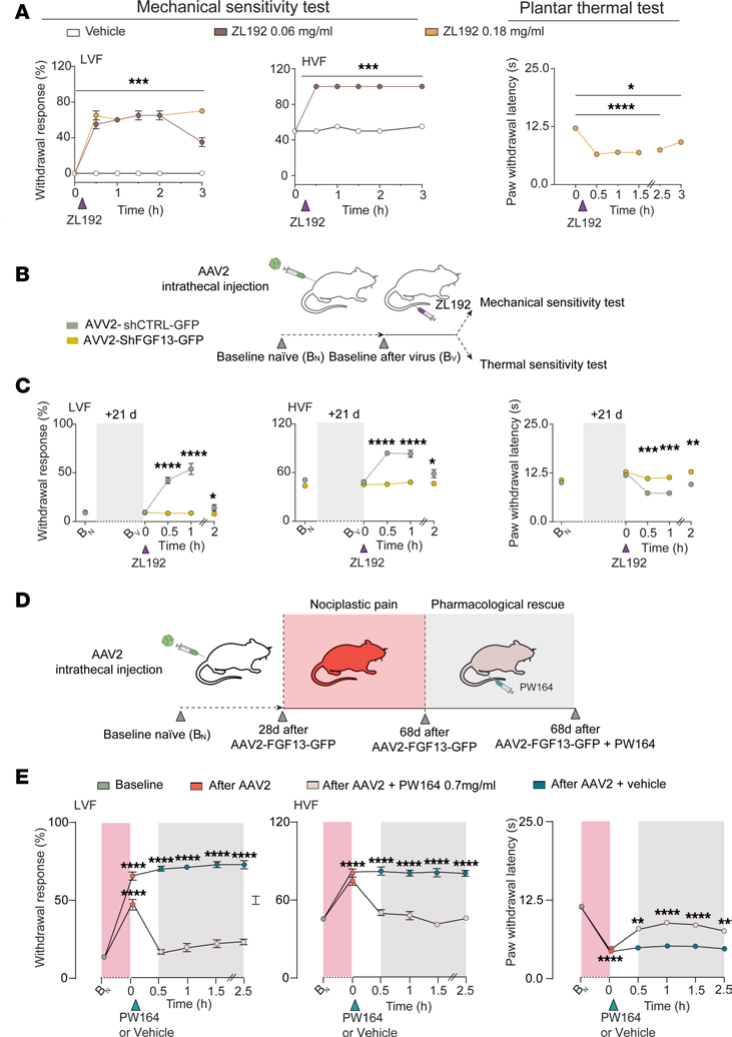
ZL192 exhibits pronociceptive roles that are prevented by in vivo FGF13 silencing and recapitulated in FGF13 overexpression. (**A**) Paw withdrawal response to LVF and HVF stimulation before and after local ZL192 injection and corresponding experiments for thermal sensitivity in indicated groups. (**B**) Illustration of experimental design showing lumbar intrathecal injection of viral particles and tests before AAV injection (baseline naive, B_N_), or 21 days after viral injection (B_V_). (**C**) Frequency of paw withdrawal responses (in %) to LVF (left) and HVF (central) stimulation before and after ZL192 (0.18 mg/mL) intraplantar injection at different time points. Thermal sensitivity before and after ZL192 (0.18 mg/mL) intraplantar injection. (**D**) Experimental design showing lumbar intrathecal injection of viral particles, AAV2-FGF13-GFP or AAV2-GFP, in naive mice tested for mechanical and thermal responses from postinjection day 28 to day 68 and following intraplantar PW164 injection (0.7 mg/mL). (**E**) Data show development of mechanical (left and central) and thermal (right) chronic nociplastic hyperalgesia and its complete or partial pharmacological inhibition in response to intraplantar PW164 injection (0.7 mg/mL). In all groups unless noted, *n* = 12 mice/group were used except panel **A** (*n* = 4/group). Data are mean ± SEM, *P < 0.05 **P < 0.01, ***P < 0.001, ****P < 0.0001. One-way ANOVA with post hoc Tukey’s and/or 2-way ANOVA with Šidák’s multiple comparisons test.

Next, we hypothesized that ZL192 treatment alone is sufficient to potentiate Na^+^ currents in hIPSC-derived sensory neurons (RealDRG) without triggering stimuli, and that FGF13 overexpression could mimic this effect. Whole-cell patch-clamp recordings were conducted in naive hIPSC-derived sensory neurons expressing either: pAAV-GFP (vehicle only), pAAV-shFGF13-GFP (vehicle (0.1% DMSO) or ZL192 (50 μM), or pAAV-FGF13-GFP (FGF13 overexpressing; vehicle or FGF13 inhibitor PW164 (50 μM)). Recordings were performed in the presence of the Nav1.8 blocker (A-803467, 300 nM). In naive neurons (black), ZL192 (grey) potentiated I_Na_ (Figure 5A), induced hyperpolarizing shifts in V_1/2_ of activation (Figure 5B) and SSI (Figure 5C), and reduced use dependency (Figure 5D) and LTI (Figure 5E). Remarkably, these phenotypes were abolished by silencing FGF13 (Figure 5, A–E, light green). FGF13 overexpression in pAAV-FGF13-GFP neurons greatly potentiated I_Na_ compared with neurons expressing pAAV-GFP (Figure 5A, maroon versus pink); potentiation that was fully attenuated by PW164 (Figure 5A, blue). Other parameters related to Na^+^ currents were sensitive to PW164 (Figure 5, A–E, blue) but not significantly different from pAAV-GFP neurons (Figure 5, A–E, pink). Conversely, ZL192 caused depolarizing shifts in V_1/2_ of activation (Figure 5B) and SSI (Figure 5C), and inhibited use dependency (Figure 5D) and LTI (Figure 5E) of Na^+^ currents compared with vehicle control. Differences between ZL192 treatment and FGF13 overexpression on Na^+^ current modulation may arise from FGF13 overexpression’s impact on peak I_Na_, obscuring other regulatory mechanisms or inducing compensatory changes that mask additional phenotypes. No appreciable changes in parameters related to Na^+^ currents were observed in hIPSC-derived sensory neurons from the ProTx II group (Supplemental Figure 14). This indicates that: (a) FGF13 ligands stabilizing the FGF13/Nav1.7 complex or FGF13 overexpression are sufficient to potentiate Na^+^ currents and (b) FGF13 modulation can autonomously regulate nociceptive signaling, independent of receptor-mediated activation. Summary data are in Supplemental Table 8. Based on our hIPSC-derived sensory neuron findings, we tested whether ZL192 induced mechanical and thermal hypersensitivity and whether this was FGF13 dependent. Naive WT mice receiving i.pl. injection of ZL192 (5 μL, 0.06 mg/mL or 0.18 mg/mL) were tested for mechanical and thermal sensitivity (Figure 6A). Remarkably, one injection of ZL192 at either dose increased the response to both LVF and HVF stimulation lasting up to 2 hours compared with vehicle (Figure 6A), an effect also seen in thermal sensitivity tests (Figure 6A). Next, the in vivo PW164 strategy was applied to test ZL192 activity in mice that received i.t. injection (L4-L6) of either AAV2-shCTRL or AAV2-shFGF13 viral particles (10 μL; Figure 6B). Mechanical and thermal sensitivity were evaluated prior to and 3 weeks after injection, and immediately before testing with ZL192 and showed unaltered withdrawal and latency responses (Figure 6C). Notably, ZL192 caused mechanical and thermal hypersensitivity in AAV2-shCTRL mice (0.18 mg/mL, i.pl.), (Figure 6C), phenotypes absent in AAV2-shFGF13 mice (Figure 6C), supporting in vivo target validation of ZL192. Next, we tested whether prolonged in vivo overexpression of FGF13 in naive mice induces chronic hyperalgesia without noxious stimuli. Supporting this, i.t. injection of AAV2-FGF13-GFP (10 μL) induced mechanical allodynia, hyperalgesia, and thermal hypersensitivity (Figure 6, D and E, and Supplemental Figure 15A). At day 68 (Figure 6E), AAV2-FGF13-GFP–expressing mice received i.p. PW164 (5 μL, 0.7 mg/mL), resulting in complete recovery from allodynia and hyperalgesia, and partial rescue from FGF13 overexpression–induced thermal hypersensitivity lasting up to 2.5 hours (Figure 6E). Complete recovery from thermal hypersensitivity was obtained at 7 mg/mL (5 μL) of PW164 (Supplemental Figure 15B). The overexpression efficacy of AAV2-FGF13 is shown in Supplemental Figure 11. These findings indicate that either ZL192 or overexpression of FGF13 is sufficient to potentiate Nav1.7 currents in hIPSC-derived sensory neurons, which leads to pronociceptive behaviors in vivo.

### PW164 reduces mechanical hyperalgesia in a high fat–diet model of T2DN.

**Figure 7:**
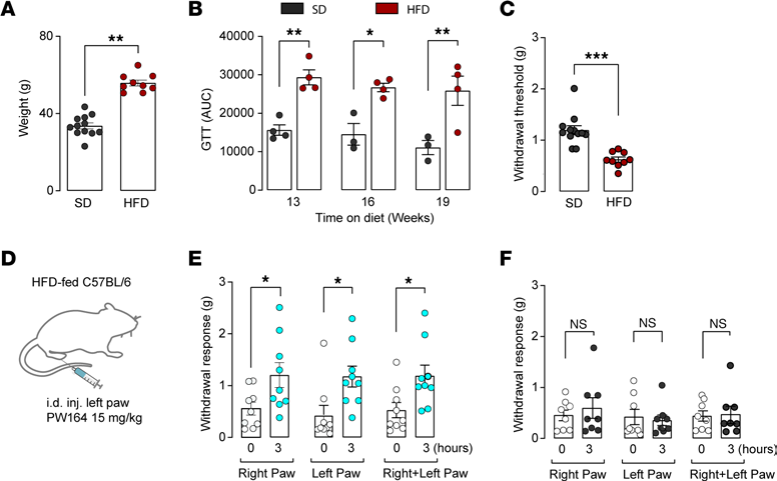
PW164 reduces mechanical hyperalgesia in the HFD-induced T2DN mouse model. (**A**) Body weights of mice fed a high fat diet (HFD) compared with standard chow diet (SD). (**B**) Glucose tolerance testing (GTT) performed at 13, 16, and 19 weeks. After measuring baseline fasting blood glucose levels, glucose (1 g/kg body weight) was injected i.p. and blood glucose measured at 15, 30, 60, and 120 minutes. For statistical analyses, AUC was calculated for each mouse. The mean ± SEM of each time point is shown. (**C**) Mice fed a HFD develop pain, shown as a decrease in the average 50% hindpaw withdrawal threshold upon von Frey stimulation of both hindpaws. (**D**) Experimental design showing intradermal PW164 injection (i.d.) (15 mg/kg) in the left paw. (**E**) Bar graph showing paw withdrawal response (both left and right paws) to von Frey stimulation before and after PW164 injection to left paw at 0 and 3 hours after PW164. (**F**) Bar graph showing paw withdrawal response (both left and right paws) to von Frey stimulation before and after vehicle (DMSO) injection to left paw at 0 and 3 hours. Animal groups were noted as (*n* = 4–9). Data are mean ± SEM, *P < 0.05, **P < 0.01, ***P < 0.001, Student *t* test.

Chronic inflammatory and neuropathic pain affects over 90% of patients with Type 2 diabetes (48, 49), and links between FGF13 and diabetic neuropathy in mouse models (50) and in humans (51) have been reported. Thus, we tested whether PW164 inhibition of FGF13 is therapeutically valuable for T2DN. We used the translationally relevant and common high fat–diet induced (HFD-induced) diabetic neuropathy mouse model of human disease (50, 52). Before in vivo testing, intradermal (i.d.) PW164 pharmacokinetics were evaluated. Mice received a single 15 mg/kg i.d. dose (Supplemental Table 9). PW164 demonstrated acceptable plasma exposure (PO 20 mg/kg: AUC_0–1_ = 225.4 ± 43.4 h·ng/mL; IV 10 mg/kg: AUC_0–1_ = 634.5 ± 52.4 h·ng/mL), Cmax (PO: 364.0 ± 166.3 ng/mL; IV: 2059.1 ± 321.1 ng/mL), volume of distribution (Vz = 3.8 ± 0.71 L/kg), and plasma clearance (CL = 15.64 ± 1.15 mL/min/kg). No PW164 was detected in the brain (brain/plasma ratio = 0). Its activity against human ether-a-go-go-related gene (hERG) was assessed using the Predictor hERG Fluorescence polarization assay kit (Invitrogen) with no detectable inhibition (IC_50_ > 30 μM versus the positive control E-4031 IC_50_ = 0.022 μM, *n* = 4 sample/concentration), confirming cardiac safety. No changes in blood pressure or body weight were observed up to 8 hours after injection, confirming no impact on sympathetic function (Supplemental Table 10), unlike other Nav1.7 channel inhibitors (53–55). As expected, HFD mice gained 34% more weight and exhibited impaired glucose tolerance compared with those on a standard diet (Figure 7, A and B). Glucose tolerance is shown by AUC analysis (SD: 15,619.0 ± 1,349.0 at 13 weeks, 14,522.6 ± 2,819.8 at 16 weeks, 11,075.3 ± 1,839.7 at 19 weeks and HFD: 29,340.2 ± 1,949.0 at 13 weeks, 26,722.7 ± 1,090.8 at 16 weeks, 25,845.2 ± 3,811.5 at 19 weeks, Figure 7B). After at least 19 weeks of HFD feeding, 41% mice developed an average 52.17% increase in mechanical sensitivity (SD: 1.2 ± 0.08, HFD: 0.62 ± 0.05, *n* = 12 and 9; Figure 7C) and were subsequently treated with PW164. At 3 hours after injection, both the left and right hindpaws showed an increase in 50% hindpaw withdrawal threshold (left hindpaw: 0 hours, 0.44 ± 0.16; 3 hours, 1.1 ± 0.2; right hindpaw: 0 hours, 0.31 ± 0.07; 3 hours, 1.2 ± 0.24, *n* = 9; Figure 7E). However, animal groups injected with vehicle showed no significant effects in 50% hindpaw withdrawal (Figure 7F). Increased 50% hindpaw withdrawal demonstrates the analgesic effects of PW164 (Figure 7, D–F).

### FGF13/Nav1.7 protein complex is a translationally relevant target associated with diabetic neuropathy.

**Figure 8:**
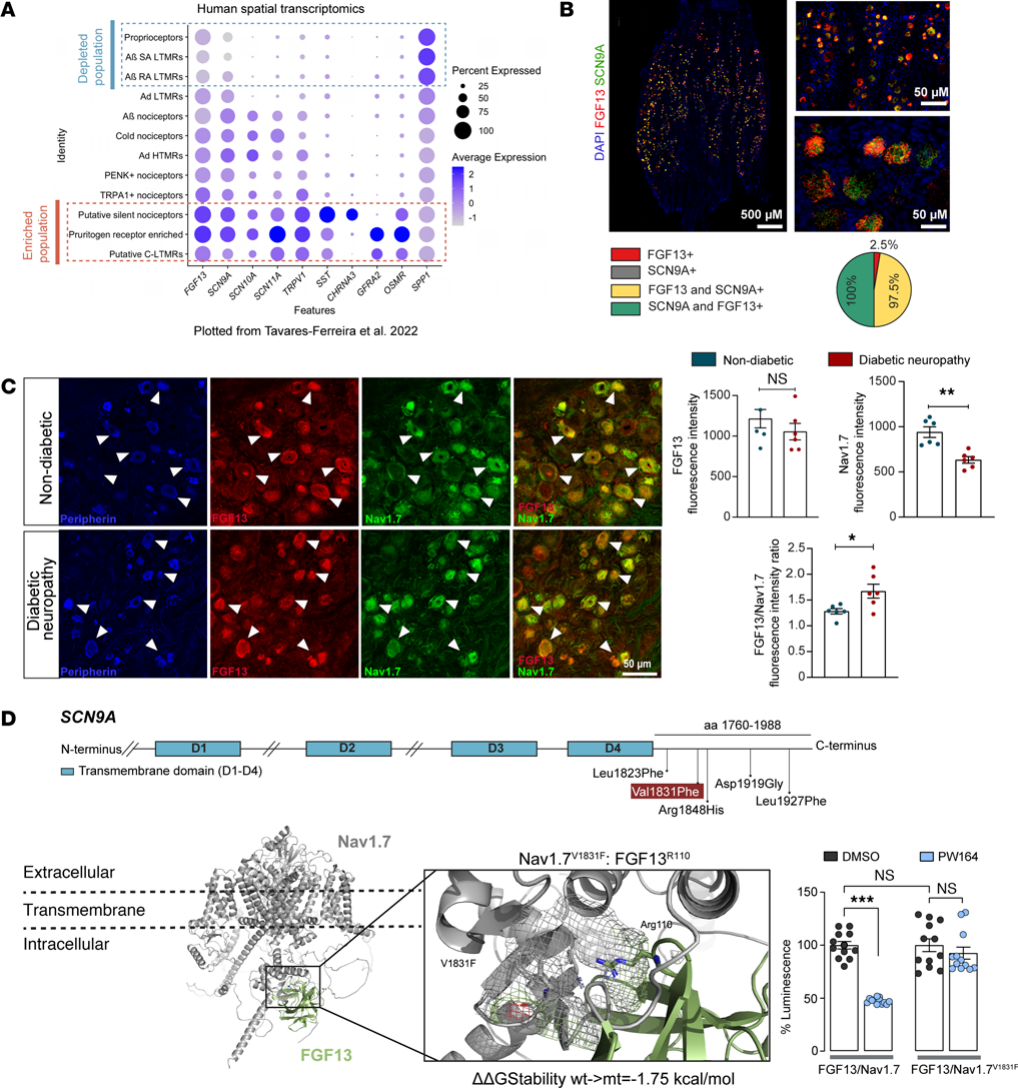
Changes in FGF13/Nav1.7 complex formation correlate with diabetic neuropathy. (**A**) Expression profile of FGF13 and SCN9A in molecularly defined neuronal subtypes, obtained from previous investigations of spatial transcriptomics in human dorsal root ganglia (47). (**B**) RNAscope-based expression pattern of FGF13 (red) and SCN9A (green) in donor-derived DRG neurons. The nuclear marker 4′,6-diamidino-2-phenylindole (DAPI) is shown in blue in the 3 panels on the left. A pie chart summarizes the percentage of neurons expressing each individual probe or pairs of probes. Quantification was done from 3 donors. Scale bars: 500 um (left); 50um (right top and bottom). (**C**) Representative confocal images from human nondiabetic (NDC) controls and patients with type-2 diabetic neuropathy (T2DN) DRG neurons stained with anti-FGF13 antibody (red), anti-Nav1.7 antibody (green), and peripherin (blue). To the right, summary bar graphs of mean and ratio fluorescence intensity for NDC versus T2DN. 21. White arrowheads indicate representative cells exhibiting FGF13/Nav1.7 colocalization. Scale bar: 50 μm. (**D**) Schematic illustration of *SCN9A* rare variants in the coding region of the Nav1.7 CTD found associated with neuropathies (top). ackFold model of the Nav1.7/FGF13 complex; the interaction with FGF13 occurs intracellularly. Close up showing the V1831F residue in direct interaction with the R110 residue on FGF13. R110 is a structural determinant of the FGF13/Nav1.7 CTD PPI interface. DDmut predicts the V1831F mutation to destabilize the FGF13/Nav1.7 complex ΔΔGStability = –1.75 kcal/mol. On the bottom right, LCA analysis of FGF13/Nav1.7 vs FGF13/Nav1.7 V1831F mutation does not alter complex assembly but prevents PW164’s pharmacological activity. Data are mean ± SEM, ns = not significant, *P < 0.05; **P < 0.01; ***P < 0.001, 1-way ANOVA post-hoc Tukey HSD (**D**); Mann Whitney test (**C**).

Discrepancies in pain mechanisms between preclinical models and humans (12) impede pain therapeutic development. Consequently, we sought to validate FGF13 and Nav1.7 expression in human samples. We first examined a gene panel, including *FGF13*, *SCN9A,* and several marker genes, in human single-cell transcriptomic data by mining previously published data (56) (Figure 8A). *FGF13* and *SCN9A* showed similar enrichment patterns to the mouse transcriptomics data (Supplemental Figure 10), with highest levels of expression in the putative silent nociceptors and pruritogen receptor enriched populations. Next, we assessed the impact of the FGF13/Nav1.7 complex on excitability of human donor–derived DRG neurons from healthy controls (HC) (57, 58). For this, we applied RNAscope to confirm expression of *FGF13* and *SCN9A* transcripts (Figure 8B). Colocalization analysis revealed almost complete overlap in expression of the 2 transcripts; 100% coexpression of *FGF13* in neurons positive for *SCN9A* (Figure 8B) corroborated the necessity of FGF13 as an accessory subunit of Nav1.7 in these cells. Next, whole-cell current-clamp recordings were performed on the DRG neurons 1 hour after preincubation with either PW164, ZL192, or vehicle. Notably, neurons exposed to PW164 exhibited an increased rheobase to fire an action potential and reduced action potential firing relative to control, while neurons exposed to ZL192 exhibited a decreased rheobase to fire an action potential (Supplemental Figure 16). These results demonstrate that FGF13-modulation of DRG neuron activity is conserved across species and can be pharmacologically targeted.

To complement our in vivo studies in HFD animal models, we compared FGF13 and Nav1.7 protein levels in donor DRG of patients with T2DN to nondiabetic control participants (NDC). FGF13 and Nav1.7 were colocalized in peripherin-positive somas in both groups (Figure 8C). Nav1.7 levels were significantly reduced in T2DNP DRG neurons compared with controls (NDC: 940.4 ± 58.5 versus T2DNP: 633.0 ± 36.3; *P* = 0.0022), while FGF13 levels remained unchanged (NDC: 1,215 ± 113.7 versus T2DNP: 1,054 ± 102.8; *P* = 0.2403). Notably, the FGF13 to Nav1.7 ratio was significantly increased in T2DNP neurons (NDC: 1.281 ± 0.052 versus T2DNP: 1.671 ± 0.135; *P* = 0.0411), indicating increased complex formation in disease.

Next, we examined the presence of rare genetic variants in *FGF13* and *SCN9A* within the Besta cohort (59) and the PROPANE study cohort (60), which include patients with various chronic pain and painless conditions. Our focus was on mutations that were: (a) rare in the general population (less than 1% in GnomAD_total); (b) located in coding exons of FGF13 and/or the CTD of Nav1.7; (c) not carried by healthy controls. Of seven rare variants in the *SCN9A* gene and one in the *FGF13* (Figure 8D and Supplemental Table 11) meeting these criteria, the V1831F variant (NM_001365536.1 2:167055658_C/A c.5491G>T) in the *SCN9A* gene found in a painless diabetic neuropathy patient was particularly relevant. Notably, in the WT Nav1.7 protein the V1831 site is at the PPI interface of the FGF13/Nav1.7 CTD complex (Supplemental Figure 1) and is predicted to bind via hydrophobic interactions to FGF13^R110^, the ligand site of PW164 and ZL192. DDMut (61) predicted that the mutant V1831F destabilizes FGF13/Nav1.7 channel interaction with a change in protein-free energy values (ΔΔG) of –1.75 kcal/mol compared with WT (Figure 8D). We incorporated the V1831F mutation at the Nav1.7 CTD and used the LCA to assess FGF13/ Nav1.7^V1831F^ complex formation in the presence of PW164 and vehicle. Notably, while the V1831F mutation did not inhibit complex formation under basal conditions, it blocked the PW164-modulation of the FGF13/Nav1.7^V1831F^ complex (Figure 8D).

## Discussion

While consistent with the emerging role of FGF13 in neuronal function (62–67), our findings have broader implications. We show that pharmacological modulation of the FGF13/Nav1.7 complex — using 2 structurally diverse ligands with opposing effects — can exert anti- or pronociceptive effects. By combining in vitro studies with in vivo genetic silencing, overexpression in rodents, and hIPSC-derived sensory neurons, we propose a rheostat mechanism for FGF13 in nociception regulation. Pharmacological inhibition of the FGF13/Nav1.7 complex reduces mechanical hyperalgesia in the HFD-induced T2DN mouse model, a result that aligns with increased colocalization of FGF13/Nav1.7 in T2DN patient DRG neurons. The Nav1.7^V1831F^ variant, associated with painless diabetic neuropathy, is located at the FGF13/Nav1.7 PPI interface and loses its sensitivity to PW164.

Studies show that FGF13 genetic knockdown alleviates nociceptive behavioral responses (14). Our findings define a rheostat mechanism by which FGF13 regulates nociception and demonstrate that its function can be pharmacologically and genetically tuned bidirectionally.

Under basal conditions, FGF13 remains functionally silent, with no contribution to Na^+^ currents or normal sensory function but becomes activated by pain-related (in vitro) or pain-inducing (in vivo) stimuli. Consistent with this, the FGF13 inhibitor PW164 suppressed Na^+^ currents only in capsaicin-stimulated hIPSC-derived sensory neurons. PW164 had no effect on Na^+^ currents in neurons lacking FGF13 or treated with the Nav1.7-specific toxin ProTxII, confirming its specificity for FGF13-sensitized Nav1.7 currents. FGF13 silencing on Na^+^ currents mirrored that of PW164; reduced FGF13 expression in hIPSC-derived sensory neurons did not affect unstimulated Na^+^ currents but restored hyperactive Nav1.7 channels to baseline levels. In vivo, PW164 alleviated capsaicin-induced mechanical and thermal hypersensitivity without affecting normal sensory function, unlike the broad suppression of sensory function seen with local anesthetics (68). Thus, PW164 selectively blocked pain-related signals in vitro and in vivo. Additionally, silencing FGF13 with intrathecal delivery of AAV2-shFGF13 prevented mechanical and thermal hypersensitivities, and PW164 had no further effect on paw withdrawal responses or latency, an experiment that served as target validation of the FGF13 ligand. Our data suggests that the FGF13/Nav1.7 complex forms under normal physiological conditions but remains functionally silent. Rapid activation may serve as a molecular sensor for acute noxious stimuli, whereas persistent activation, as seen with FGF13 overexpression, drives pathological hyperalgesia independent of extracellular triggers.

The effect of the FGF13 positive modulator ZL192 in neurons and in vivo supports the hypothesis of a dormant physiological reservoir of FGF13/Nav1.7 protein complexes. In unstimulated hIPSC-derived sensory neurons, ZL192 potentiated Na^+^ currents, and, in vivo, it induced acute mechanical and thermal allodynia. This indicates that the FGF13/Nav1.7 complex is readily available and can be rapidly activated on demand and autonomously from noxious stimuli. Likewise, FGF13 overexpression mimicked the effect of ZL192, enhancing Na^+^ currents in hIPSC-derived sensory neurons without capsaicin and causing mechanical and thermal allodynia. However, the effect of ZL192 in vivo was acute, while FGF13 overexpression led to chronic hyperalgesia. These differences may arise from ZL192 transiently activating the FGF13/Nav1.7 complex, while an increased FGF13 protein pool could persistently alter the complex’s stoichiometry with effects on membrane trafficking via β-tubulin interactions (41, 66).

In studying the role of FGF13 in DRG neuron function and nociception, we found some discrepancies with the published literature. Effraim et al. showed in a 2022 study that overexpression of FGF13 in rat cultures protected against DRG hyperexcitability (16). This difference might be due to variations in the model, species, and their balance of endogenous and overexpressed FGF13 isoforms. The 2017 study by Yang et al. (14) showed deficiency only in thermal sensitivity, whereas we demonstrate that silencing *Fgf13* or local delivery of PW164 prevents both mechanical and thermal hyperalgesia. Similarly, overexpression of FGF13 induces chronic mechanical and thermal hyperalgesia. These differences might stem from the genetic strategy in *SNS*-*Cre/Fgf13*^F/Y^ mice, which could spare some DRG neurons, as not all FGF13-containing neurons express Nav1.8. Alternatively, the genetic strategy may induce compensatory mechanisms or adaptive changes in the genetic landscape, affecting pain pathways differently than more acute approaches such as pharmacological intervention or AAV silencing.

Given FGF13’s association with diabetic neuropathy in mouse models (50) and that altered FGF13 expression has been observed in patients with T2DN (51), we evaluated in vivo efficacy of PW164 in the T2DN HFD model. Intradermal PW164 effectively reduced mechanical hyperalgesia at both the injection and contralateral sites, indicating systemic exposure. Importantly, PW164 demonstrated favorable safety, with no systemic effects or adverse cardiac or sympathetic outcomes; an improvement over other Nav1.7 modulators (53–55). Previous reports describe decreased FGF13 levels in serum from patients with T2D (51). Although the Nav1.7 level was lower in T2DN neurons, the FGF13/Nav1.7 ratio increased, suggesting enhanced complex formation under disease conditions. These findings, consistent with animal studies (50), highlight the translational potential of PW164 and related FGF13 ligands for T2DN pain therapies. To link FGF13/Nav1.7 complex formation to clinical pain, we screened rare FGF13 and SCN9A variants in the Besta (59) and PROPANE (60) cohorts. The Nav1.7^V1831F^ variant preserved baseline FGF13/Nav1.7 binding but blocked PW164 modulation, suggesting compromised PPI interface integrity. Future studies will examine its impact on Na^+^ currents and pain perception.

Decades of studies in pain mechanisms have highlighted Nav1.7’s role in nociception (69–71) with extensive efforts targeting this channel without satisfactory clinical results (72). Failure of Nav1.7 clinical translation is attributed to a lack of in vivo efficacy or severe side effects (5, 73) not anticipated in preclinical models. Discrepancy between human and preclinical studies in both pharmacological and genetic studies targeting Nav1.7 (74) raises the need for fresh approaches (53, 75). New classes of inhibitors made possible by recent Nav channel structural resolution (76–80) or based on toxin derivatives (81, 82) are under investigation (83–85), along with Nav1.7 interactome modulators (13, 38, 40, 86–88).

FGF13-based PPI modulators have not been studied and differ mechanistically from traditional agonists, antagonists, and known allosteric modulators of membrane receptors, because they act intracellularly, likely via complex intermolecular interactions during the channel cycle (89). PW164’s antagonistic effect on FGF13 regulation of Na^+^ currents is likely due to its direct binding to the FGF13^R110^ site (20, 28, 29, 90), disrupting FGF13 interactions with the Nav1.7 CTD. However, its seemingly opposite effects on voltage dependence, inactivation, use dependence, and repriming may result from dynamic regulation of intramolecular interactions with domains beyond the CTD as the channel progresses through its cycle (91–93). Conversely, ZL192 appears to act more as a “molecular glue,” stabilizing the FGF13/Nav1.7 complex throughout the channel cycle. Its mechanism, likely driven by strong bridging interactions between R110 and M1830 via salt bridges, shows more consistent potentiation of the FGF13-induced phenotypes on Na^+^ currents, except for a modest effect on V_1/2_ of activation. Future studies will focus on structure-activity relationships and targeted mutations to gain further insights into these and other PPI modulators for pain management and neurotherapeutics (89). Here, we present evidence for FGF13 as a rheostat of nociception and a translationally relevant target for pain therapeutics and probes for pharmacogenomic studies, patient stratification (94), and disease biomarkers (95).

## Methods

### Sex as a biological variable.

Only male mice were used in in vivo studies due to documented sex-specific differences in nociceptive processing, which could confound the interpretation of treatment effects. For DRG analyses, only male donor samples were included due to high variability observed in female samples, which limited statistical reliability in this cohort.

### Study design.

This study investigated the role of the FGF13/Nav1.7 PPI complex in nociception and pain using in silico analyses, in-cell split-luciferase assays, surface plasmon resonance, whole-cell patch clamp electrophysiology, RNAscope, immunocytochemistry, and behavioral assays in rodent models of acute and chronic pain. Cell lines stably or transiently expressing WT or mutated genes of interest, along with commercially available human iPSC-derived RealDRG were used. We tested whether manipulations affecting FGF13/Nav1.7 complex formation or FGF13 expression correlated with changes in Na^+^ currents in RealDRG and nociception, consistent with increased interaction leading to hyperalgesia and decreased interaction preventing hyperalgesia. These findings were validated in vivo using gene transfer to silence or overexpress FGF13 in rodent models. Human studies were conducted in donor-derived DRG neurons, including RNAscope, patch clamp electrophysiology, data mining of deposited data sets, postmortem DRG tissue, and genetic analysis from the Besta and PROPANE study groups. FGF13 ligands were administered in vivo to assess impact on nociception and explore their potential for pain management. Sample sizes were determined through power analysis or prior experience, with predetermined endpoints. There were no specific inclusion/exclusion criteria, and outliers were not treated. Random assignment of mice to experimental groups was ensured, and procedures were performed blinded. Sample numbers for all studies are in figure legends or tables. Additional details for all experimental methods are provided in the supplementary material.

### Statistics.

Statistical values were calculated as mean ± SEM, unless otherwise specified. Data were first analyzed for normal distribution and log-normalized where appropriate. Power analyses determined in vivo study sample sizes. After analyzing dataset variance differences, Welch’s corrections were applied where appropriate. For normally distributed samples, statistical significance between different groups (*P < 0.05) was determined using Student’s *t* test or 1-way ANOVA followed by post hoc Bonferroni’s test or Tukey’s multiple comparisons test. For samples not normally distributed, the Kruskal-Wallis 1-way ANOVA on ranks with post hoc Dunn’s method was used. Two-way ANOVA with Šidák’s multiple comparisons test was also used when appropriate. Analysis was performed using Sigma Stat or GraphPad Prism software version 9. Blinding and randomization to mitigate bias were applied before analysis. Heat maps illustrating compound performance were generated with Heatmapper. For dose response, nonlinear regression curve fitting to determine IC_50_/EC_50_ values was performed using GraphPad Prism. Data were plotted using GraphPad and final figures compiled using Adobe Illustrator (27.2). Additional statistical analyses and data collection are provided in Supplemental Materials.

### Study approval.

All experiments presented were performed in accordance with the NIH guidelines for the Care and Use of Laboratory Animals and were approved by the University of Texas Medical Branch Institutional Animal Care and Use Committee. The human dorsal root ganglia (DRGs) procurement procedures were performed in accordance with the Institutional Review Boards at the University of Texas at Dallas. DRGs were procured from organ donors through a collaboration with the Southwest Transplant Alliance (STA). All human clinical data were obtained under protocols approved by the appropriate IRBs, and all samples were deidentified prior to analysis to ensure compliance with ethical and regulatory standards.

### Data availability.

All data supporting the findings of this study are reported in the Supporting Data Values file. Genetic data utilized in this study from patients with chronic pain are available at the Fondazione IRCCS Istituto Neurologico Carlo Besta, namely the “Besta cohort” and the “PROPANE 454 study cohort.”

## Author contributions

AKS performed LCA experiments, HEK293 cell and RealDRG electrophysiology and SPR, data analysis, and writing. MB did behavioral tests, intrathecal injections, and writing. NMD performed electrophysiological recordings. ZH performed homology modeling, docking, and virtual screening. JDR did confocal imaging. OOF did LCA and behavioral tests. FKK performed human DRG neuron recordings. LAM did RNAscope analysis in human DRG neurons. PAW performed LCA experiments. CMT did electrophysiological recordings. P Wang did FGF13 ligand chemical synthesis. JW gave technical assistance for behavioral testing. HC did homology modeling and docking. YX did FGF13 ligand chemical synthesis. KH provided behavioral testing assistance. JS performed mutagenesis and LCA. DD did pAAV-shRNA subcloning. VT and P Walsh performed RealDRG\RNAScope and confocal imaging. MM, E Salvi, ID, and GLP did genetic analysis. CGF, RAM, MDT, and DZ provided patient data on behalf of PROPANE study group. MRS and MJI did bioinformatic analyses. PA provided interpretation of electrophysiology. TAG provided guidance in shRNA hairpin design. PMG gave intellectual contribution to electrophysiological data interpretation. KR did SPR analysis. DPG and SY guided intrathecal injections and behavioral testing. MKW, E Salisbury, SY, and IJG performed i.d. injections and behavioral testing in T2DN mouse models. SIS, NK, and NAG, performed confocal imaging of T2DNP patient DRGs. TJP provided guidance, interpretation, and analysis of DRG neurons and overall feedback on the manuscript. GDC, DSC, and HL provided guidance, interpretation, and analysis of DMPK studies. JZ contributed to the chemical design of FGF13 ligands. SD contributed to electrophysiology studies in human donor DRG neurons. JHL and JMC contributed to behavioral testing. FL conceived the project, designed experiments, and provided intellectual guidance. FL, along with AKS and MB, wrote the manuscript.

## Supplementary Material

Supplemental data

Supporting data values

## Version 1 07/15/2025

Electronic publication

## Version 2 08/20/2025

Correction to Grant R01NS122571 to SY
