# Supplementary material for: Sensory neuron–expressed FGF13 controls nociceptive signaling in diabetic neuropathy models
Source: J Clin Invest. 2025 Jul 15;135(14):e183749. doi: 10.1172/JCI183749 (PMC12259270; doi:10.1172/JCI183749)
Supplement: Supplemental data [file jci-135-183749-s009.pdf]

Supplementary Materials

Sensory neuron-expressed FGF13 controls nociceptive signaling in diabetic neuropathy models

Aditya K. Singh[1#], Matteo Bernabucci[1#], Nolan M. Dvorak[1], Zahra Haghighijoo[1], Jessica Di Re[1], Nana A. Goode[1], Feni Kadakia[2], Laura Maile[2], Oluwarotimi O. Folorunso[1,&], Paul A. Wadsworth[1,%], Cynthia M. Tapia[1], Pingyuan Wang[1], Jigong Wang[2], Haiying Chen[1], Yu Xue[1], Jully Singh[1], Kali Hankerd[3,$], Isaac J. Gamez[3], Makenna Kager[6], Vincent Truong[7], Patrick Walsh[7], Stephanie I. Shiers[17], Nishka Kuttanna[17], Hanyue Liao[18], Margherita Marchi[8], Erika Salvi[8], Ilaria D'Amato[8], Daniela D'Amico[1], Parsa Arman[1], Catharina G. Faber[9], Rayaz A. Malik[10,11], Marina de Tommaso[12], Dan Ziegler[13], Krishna Rajarathnam[5], Thomas A. Green[1], Peter M. Grace[4], Matthew R. Sapio[14], Michael J. Iadarola[14], Jun-Ho La[3], Jin Mo Chung[3], Jia Zhou[1], Gregory D. Cuny[19], Diana S. Chow[18], Giuseppe Lauria Pinter[15,16], Steve Davidson[2], Dustin Green[3], Theodore J. Price[17], Elizabeth Salisbury[6], Subo Yuan[3] & Fernanda Laezza[1,5*]

Supplementary Materials

1. Methods
2. Supplemental Figures 1-16
3. Supplemental Tables 1-11

Methods

In silico molecular modeling

Homology modeling (template base). The FGF13/Nav1.7 homology model was built with the automated protein structure homology-modeling server Swiss-Model using FGF13/Nav1.5/CaM ternary complex crystal structure (PDB ID:4DCK) as a template. The FASTA file of FGF13 (aa 64-211) and Nav1.7-CTD (aa 1764–1916) sequences were downloaded from www.uniprot.org. The homology model was generated based on the target-template alignment using ProMod3 and a Protein Data Bank file was created. This coordinate file was then submitted to the Swiss-Model server for an evaluation and structure assessment. QMEANDisCo global score was 0.84 ± 0.05, MolProbity Score was 0.72.

AF2-multimer (template-free) structure prediction. To discover the potential protein/protein interaction sites, the FASTA file of FGF13 (aa 64-211) and Nav1.7-CTD (aa 1764–1916) sequences was performed using AlphaFold_2.0_multimer.v3 (AFM V3) implemented through a Jupyter Notebook on Google Colab in ChimeraX. To validate the best model, they were superimposed onto the available X-ray crystal structures of iFGFs:Nav PDB code 4DCK with RMSD 0.102Å. Both the homology model and AF2-multimer model of FGF13/Nav1.7 demonstrated the same protein-protein interaction. Analysis of the FGF13/Nav1.7 interface was then performed with Maestro Schrodinger. Subsequently, hot spot residues on the FGF13/Nav1.7 complex homology model were identified using Schrodinger Protein Interaction Analysis. The neighboring pair of interacting residues from each chain on the homology complex was set at 4.0 Å distance.

Molecular docking of ligands. The molecular docking study was performed using the Schrödinger Small-Molecule Drug Discovery Suite (Schrödinger, 2020). The FGF13 crystal structure (3HBW) was prepared with the Schrödinger Protein Preparation Wizard using default settings. During this step, hydrogens were added, crystal waters were removed, and partial charges were assigned using the OPLS-2005 force field. The SiteMap (Schrödinger) calculation was performed on FGF13, and a potential binding site was identified at the interface of the FGF13/Nav1.7 complex. Glide Grid generation was performed on this predicted site. The grid center was selected on the following coordinates: X=26.91, Y=-14.83, Z=-17.00; the grid box was sized in 20 Å on each side to cover the FGF13 surface. The structure of ligands was created using Schrödinger Maestro and a low energy

conformation was calculated using LigPrep. Ligand docking was employed with Glide using the SP protocol. The highest scoring docked poses were incorporated into Schrödinger Maestro for visualization of the binding interface. The docked pose of PW164 and ZL192 was superimposed to the FGF13/Nav1.7 complex homology model for an overlay analysis using the Schrödinger suite (Schrödinger Release 2023-4: SiteMap, Schrödinger, LLC, 2023).

To predict the effects of mutations on FGF13/Nav1.7 stability, we utilized DDMut a rapid and precise network designed for predicting changes in Gibbs Free Energy resulting from single-point mutations. The corresponding AF2 structure of full length of Nav1.7 with UniProt ID: Q15858 was retrieved from (https://alphafold.ebi.ac.uk), superimposed, and replaced in the model. To assess the validity of the structures, we conducted protein structure analysis using PROSA (https://prosa.services.came.sbg.ac.at/prosa.php), resulting in an overall quality Z-score of -3.99 and local model quality evaluation.

Chemicals

A collection of rationally designed compounds targeting the iFGF/Nav channel PPI interface were designed and synthesized as previously described (21). All compounds were reconstituted in dimethyl sulfoxide (DMSO; Sigma-Aldrich) to obtain a stock solution of 50 mM. D-luciferin substrate was purchased from Gold Biotechnologies and reconstituted in phosphate-buffered saline (PBS) to achieve a stock concentration of 30 mg/ml before storage as 1.25 ml aliquots at -20 °C. The Nav1.7-CTD (1876-1899 including the FGF13 binding domain peptide (14) was synthesized from Ontores Biotechnologies Co. The Nav1.8 channel inhibitor, A-803467, SB-366791, and the Nav1.7 channel inhibitor, ProTx II, were purchased from Sigma-Aldrich, MedChemExpress LLC (cat # 5.08505), and Peptide International Inc. (cat # 07PTX002), respectively. A-083467 and SB-366791 were reconstituted in DMSO and brought to a stock concentration of 300 µM and 10 mM while Pro TX II was reconstituted in H_2O and brought to a stock concentration of 100 µM, after which all were stored at -20 °C.

Plasmids

The respective gene ID numbers used in this study are as follows: NM_033642 (human, *FGF13-1b*) and NM_002977 (human *SCN9A*). Full-length FGF13-1b (aa 1-192) was subcloned into appropriate plasmids for use in this investigation, unless otherwise noted. For consistency with AlphaFold modeling presented, numbering of point mutations to FGF13-1b are based on the longest FGF13 isoform (FGF13-1a, NM_004114.2). CLuc-FGF13, CLuc-FGF13^{R110A}, CD4-Nav1.7-CTD-NLuc, CD4-Nav1.7-CTD^{M1830A}-NLuc, CD4-Nav1.7-CTD^{V1831F}-NLuc, and constructs expressing the CLuc or the NLuc fragments of Firefly (*Photinus pyralis*) luciferase (24) respectively were engineered, mutated (CLuc-FGF13^{R110A}) and sequenced at the Molecular Genomics Core (University of Texas Medical Branch). The CD4-Nav1.7-CTD-NLuc cassette was synthesized from GeneArt (Nav1.7 residues 1780-1977) (ThermoFisher) and subcloned at 5'-HindIII and 3'-BsiWi restriction sites in pcDNA3.1 (Invitrogen). Additionally, FGF13 was amplified with polymerase chain reaction (PCR) by using gene-specific primers, and subcloned in pcDNA3.1 at 5'-BsiWi and 3'-Not I restriction sites. For protein expression and purification in the bacterial system, the pET expression bacterial vector pETDuet-1-FGF13U was used as described previously (29). The pQBI25/50fc1-FGF13-1a or 1b-GFP (FGF13-1a-GFP or FGF13-1b-GFP) and pQBI25/50fc1-GFP (GFP) constructs were used for whole-cell patch-clamp electrophysiology in HEK293 cells stably expressing human Nav1.5, Nav1.6, or Nav1.7 channels. The shFGF13 was sub-cloned in the pAAV2-shRNA-GFP vector at SapI + XbaI sites, as previously described (42). The AAV2-control-GFP, AAV2-shFGF13-GFP and AAV2-hFGF13-GFP were packaged in virus particles at the Gene Therapy Center-Vector Core (University of North Carolina). Alignment of the mRNA sequence targeted by AAV-shFGF13 across species was performed by blasting the rat mRNA sequence corresponding to the AAV-shFGF13 region using ncbi_blastn. The alignment report showed a 100% match between rat and mouse (sequence ID: AF020737.1) and a 95.83% match with human mRNA (sequence ID: U66198.1).

Cell Culture and transfections

HEK293 cells were purchased from ATCC (catalog # CRL-1573). Cells were cultured and incubated at 37 °C with 5% CO_2 in medium with equal volumes of Dulbecco modified essential medium (DMEM) and F12

(Invitrogen) supplemented with 10% fetal bovine serum, 100 U/ml penicillin, and 100 mg/ml streptomycin. HEK293 cells stably expressing human Nav1.7 (HEK293-Nav1.7) were cultured with 80 μg/ml G418 (Invitrogen) along with 100 U/ml penicillin and 100 mg/ml streptomycin to ensure stable $Na_v1.7$ expression. HEK293-Nav1.7 cells were transiently transfected with pQBI-GFP (GFP) or human pQBI-FGF13-1a or 1b-GFP (FGF13-1a-GFP or FGF13-1b-GFP) using Lipofectamine 2000 (Invitrogen). 24-48 hours post transfection, HEK293-Nav1.7 cells were plated at low density on glass coverslips and processed for electrophysiological recordings. Human IPSC-derived sensory neurons trademarked as RealDRG™ (Anatomic Incorporated 1020F1-1M) were cultured using a commercially available kit (Senso-MM, Anatomic Incorporated 1030) onto 24-well plates coated with 0.01% Ploy-L-Ornithine (Sigma-Aldrich A-004-C) plus iMatrix-511 SILK (1:100, Anatomic Incorporated M511S) or iMatrix-coated plastic coverslips (Thermo Scientific) for 7-14 days following manufacturer's instructions. hIPSC-derived sensory neurons were transfected at day 5-10 after plating with pAAV-GFP or pAAV-shFGF13-GFP or pAAV2-FGF13-GFP using the NeuroMagTM Kit (OZ Biosciences; cat# KC30800) following manufacturer's procedure and processed for electrophysiological recordings within ~ 1 week after transfection.

Human donor DRG (hDRG) neurons. Dorsal root ganglia were recovered from consented, de-identified organ and tissue donors at the University of Cincinnati Medical Center following previously described protocols (57, 58). Recovered tissues were transported in oxygenated, ice-cold N-methyl-D-glucamine (NMDG) artificial spinal fluid (aCSF, mM): 93 NMDG, 2.5 KCl, 1.25 NaH_2PO_4, 30 $NaHCO_3$, 20 HEPES, 25 Glucose, 5 Ascorbic acid, 2 Thiourea, 3 Na+ pyruvate, 10 $MgSO_4$, 0.5 $CaCl_2$, 12 N-acetylcysteine, adjusted to pH 7.4 with HCl). hDRGs were isolated from connective tissue, fat, roots, and nerves and then minced into small 1-3 mm pieces which were enzymatically treated with warmed papain (45U) followed by collagenase (4.5 mg/200μl) each for 40 minutes at 37 ºC, 5% CO_2 in HBSS with 5 mM HEPES (Hanks' Balanced Salt Solution with 4-2 (2-hydrozyethyl)-1-piperazineethananessulfonic acid; Gibco). Neurons were dissociated by trituration and incubated in warmed Neurobasal A-based DRG media (with penicillin/streptomycin, B27 supplement (Gibco), fetal bovine serum (FBS), and GlutaMAX (Gibco, cat # 35050061), as previously described. Dissociated DRG suspension was plated onto poly-d-lysine (0.01 mg/ml, Sigma) and collagen (1 mg/ml, Sigma) coated coverslips and incubated 2 days.

Split-luciferase Complementation Assay (LCA)

Twenty-four hours post transfection, cells were trypsinized using TrypLE (Gibco; cat # 12604013), centrifuged at 1750 rpm for 5 min, triturated in medium, and seeded into white clear-bottom 96-well CELLSTAR µClear® plates (Greiner Bio-One; cat # 655073) at ~0.8 x 10^5 cells per well in 200 µl of medium. The cells were incubated for another 24 hours, and then the growth medium was replaced with 100 µl serum-free, phenol red-free DMEM/F-12 medium (Invitrogen). All compounds from the PW- and the ZL-series, including PW164 and ZL192, were dissolved in DMSO, added to a final concentration of 1–500 µM (0.5 % DMSO) in the serum-free medium and then cells were incubated for two hours. The bioluminescence reaction was initiated by injection of 100 µl of D-luciferin substrate (1.5 mg/ml dissolved in PBS) using a Synergy H4 multimode microplate reader (BioTek). Luminescence readings were initiated after 3 s of mild plate shaking and performed at 2 min intervals for 20-30 min with integration times of 0.5 s, and the cells were maintained throughout the measurements at 37 °C. Raw signal intensity was computed by averaging peak luminescence plus two adjacent time points. Normalized signal intensity was expressed as the percentage of mean signal intensity relative to per plate control wells treated with 0.5% DMSO.

Surface plasmon resonance (SPR)

All SPR experiments were performed on a Biacore T100 (GE Healthcare). Biotin-Nav1.7-CTD peptide (QEDVSATVIQRAYRRYRLRQNVK), which was previously described (14) or purified FGF13 were immobilized on CM5 sensor chip using 10 mM sodium acetate buffer (pH 5.5) with Amine Coupling Kit (GE Heathcare) and on SA sensor chip with 75% isopropyl alcohol, 50 mM NaOH as per manufacturer's instructions. The FGF13 was immobilized to the final 10050 RU, and Nav1.7 to the 10 or 1000 RU, and no protein was coupled to the control channel of the chip. For each compound, stock solution (50 mM) was serially diluted from 300 to 1 µM; the Nav1.7-CTD peptide (1876-1899, 23 amino acids, MW ~2.8 kDa) was serially diluted from 150 to 1 nM in PBS-P^+ (PBS supplemented 0.005% with tween 20). Samples were injected over the chip for 60 s followed by dissociation for 120 s at the flow rate 40 µl/min; finally, the chip was regenerated using the solution of 1.5 M NaCl, 3% DMSO for 180 s. The interaction of compound or un-conjugated Nav1.7-CTD peptide with FGF13, as

well as a compound with the Nav1.7-CTD peptide was studied at 25 °C. The nonspecific responses (buffer alone) were extracted for injected compound or peptide from experimental sensograms before data analysis. Data was analyzed for kinetic properties using Biacore T100 Analysis software. Visually inspected sensograms/traces were used to calculate the equilibrium constant (K_D): i. the maximal responses were plotted against the treated concentrations, and the steady state K_D was calculated from the fitted saturation binding curve; ii. a kinetic analysis for all ligand/analyte interaction was obtained by using Langmuir 1:1 interaction fitting model ($K_D = k_{off}/k_{on}$).

RNA isolation from DRG

Mouse surgical and experimental procedures employed were approved by the University of Texas Medical Branch Institutional Animal Care and Use Committee. Animals were sacrificed using carbon dioxide before confirmation of death, rather than cervical dislocation. Mouse lumbar dorsal root ganglia (L4 and L5) were dissected and collected in Buffer RLT Plus (RNeasy Plus Mini Kit, QIAGEN, cat. nos. 74136). RNA was isolated according to the protocol reported in the same kit.

Quantitative reverse transcriptase PCR (qRT-PCR)

RNA samples for real-time analysis were then quantified using a Nanodrop Spectrophotometer (Thermo Scientific). cDNA was generated from total RNA (1 µg) in a 20 µl reaction using the reagents of iScript™ Reverse Transcription Supermix for RT-qPCR (Bio-Rad Laboratories, cat. 1708840). FGF13 gene expression was determined by qRT-PCR. qRT-PCR was performed in duplicate for each sample using 4 µl of cDNA in a total volume of 20 µl using the iTaq Universal SYBR Green Supermix (Bio-Rad, Hercules, CA, United States). All RT-PCR assays were run using the BioRad CFX384 Touch Real-Time PCR System (Bio-Rad, Hercules, CA, United States) with the following conditions: 95°C (20s), 95°C (5s), 60°C (30s) repeated by 40 cycles and 4°C. RT-PCR primer sets (Bio-Rad, Hercules, CA, United States) for FGF13 were: 5′-AGG CCG AGG GTG GTA TCT G-3′ and reverse 5′-AGA TCG GGA GAA CTC CGT GAG-3′—which produced a 160 bp amplicon. The internal reference gene for SYBR Green assays was 18S 5’-CGC TCC ACC AAC TAA GAA CG-3’ and 5’-CTC

AAC ACG GGA AAC CTC AC-3'. Samples were replicated three times. The mRNA expression levels were normalized with GAPDH and 18S and relative gene expression was calculated using the ΔΔCt method.

Whole-cell patch clamp electrophysiology

HEK293-Nav1.7 cells, HEK293 stably expressing human Nav1.6 (HEK-Nav1.6), HEK293 stably expressing human Nav1.5 (HEK-Nav1.5), human hIPSC-derived sensory neurons (RealDRG™ from Anatomic Incorporated 1020F1-1M) and donor-derived DRG neurons were plated at low density on glass coverslips and processed for whole-cell patch clamp electrophysiology in voltage-clamp and/or current clamp mode. Whole-cell patch electrophysiological experiments were conducted following previously published studies (26). Current densities were obtained by dividing I_{Na} amplitude by membrane capacitance. Current–voltage relationships were generated by plotting current density as a function of the holding potential. Conductance (G_{Na}) was calculated by the following equation:

$$G_{Na} = \frac{I_{Na}}{(V_{m} - E_{rev})}$$

where I_{Na} is the current amplitude at voltage V_m, and E_{rev} is the Na^+ reversal potential.

Activation curves were derived by plotting normalized G_{Na} as a function of test potential and fitted using the Boltzmann equation:

$$\frac{G_{Na}}{G_{Na,max}} = 1 + e^{V_a - E_m/k}$$

Where $G_{Na,Max}$ is the maximum conductance, V_a is the membrane potential of half-maximal activation, E_m is the membrane voltage and k is the slope factor. For steady-state inactivation, normalized current amplitude (I_{Na}/I_{Na},Max) at the test potential was plotted as a function of prepulse potential (Vm) and fitted using the Boltzmann equation:

$$\frac{I_{Na}}{I_{Na,max}} = \frac{1}{1 + e^{V_h - E_m/k}}$$

Where V_h is the potential of half-maximal inactivation, E_m is the membrane voltage, and k is the slope factor. Transient I_{Na} inactivation decay was estimated with the standard exponential equation. Inactivation time constant (tau, τ) was fitted with the following equation:

$$I_{(t)} = I_{Na} e^{\left(-\frac{t}{\tau}\right)} + I_{SS}$$

where $I_{(t)}$ is the amplitude of the current at time t and I_{ss} is the steady-state current during a single voltage-step. The goodness of fitting was determined by correlation coefficient (R), and the cutoff of R was set at 0.85. To observe effects on long-term inactivation (LTI), a four-step protocol was employed during which cells were subjected to four 0 mV, 20 ms depolarization pulses separated by −90 mV, 40 ms recovery intervals. To control for various cell sizes, the current densities were calculated by dividing I_{Na} peak current amplitude/membrane capacitance (C_m). To represent the fraction of channels entering into LTI, the peak I_{Na} observed during depolarization cycles 2-4 was normalized to the peak I_{Na} observed during depolarization cycle 1 ($I_{\mathrm{Na}}/I_{\mathrm{Na},Cycle\ 1}$) and plotted as a function of the depolarization cycle.

The cumulative (frequency-dependent) use-dependency was determined by applying 20 pulses with a depolarization to −10 mV (50 ms duration) and 50 ms recovery intervals with a train of 20 pulses at 10 Hz from a holding potential at -70 mV. The current pulses were normalized to the first recorded pulse and the currents at the 2nd to 20th pulses were compared. The same train was used to record use-dependency at various frequencies ranging from 1 to 20 Hz.

The rate of recovery from inactivation was recorded at -80 mV with a standard repriming voltage protocol. The cells were prepulsed to -20 mV for 20 ms to inactivate all currents, then driven back to the recovery potential (Vrec) for increasing recovery durations before the test pulse to 0 mV. The maximum pulse rate was 0.5 Hz. The time course (2, 5, 9, 17, 33, 65, 129, 257, 513, 1025, and 2049 ms) used for the recovery duration for that trace follows previous publications (16). The time constants were estimated from single exponential decay to time courses measured at recovery potentials ranging from -140 to -60 mV with the protocol as mentioned above.

Electrophysiological Recordings

HEK293 cells: Electrophysiological recordings in HEK293 stably expressing Nav1.7 (HEK-Nav1.7), Nav1.5 (HEK-Nav1.5) or Nav1.6 (HEK-Nav1.6) were performed at room temperature using a MultiClamp 700B or an Axopatch 200B amplifier (Molecular Devices) after incubating coverslips for 60 minutes either with 0.1% DMSO or 50 μM PW164 or ZL192 in the extracellular solution. The composition of recording solutions consisted of the

following salts: extracellular (mM): 140 NaCl, 3 KCl, 1 MgCl2, 1 CaCl2, 10 HEPES, 10 glucose, pH 7.3; intracellular (mM): 130 CH3O3 SCs, 1 EGTA, 10 NaCl, 10 HEPES, pH 7.3. Membrane capacitance and series resistance were estimated by the dial settings on the amplifier and compensated for electronically by 70–80%. Data was acquired at 20 kHz and filtered at 5 kHz before digitization and storage. All experimental parameters were controlled by Clampex 9.2 software (Molecular Devices) and interfaced to the electrophysiological equipment using a Digidata 1300 analog–digital interface (Molecular Devices). Voltage-dependent inward currents for HEK-Nav1.7 cells were evoked by depolarization of test potentials between −100 mV and +60 mV from a holding potential of −70 mV. Steady-state (fast) inactivation of Nav channels was measured with a paired-pulse protocol. From the holding potential, cells were stepped to varying test potentials between −120 mV and +20 mV (prepulse) before a test pulse to −20 mV. Additional information regarding electrophysiological recordings is available in the supplementary information section.

hIPSC-derived sensory neurons (RealDRG^{TM}): Whole-cell voltage clamp electrophysiological recordings used the same protocol as for HEK293 cells and were conducted using a MultiClamp 700B or on Axopatch 200B amplifier (Molecular Devices) following a 60 min incubation of coverslips with either with 0.1% DMSO or 50 μM PW164 or ZL192 in the extracellular solution. The recording solution consisted of the following salts: extracellular (mM): 130 NaCl, 3 KCl, 30 TEA, 0.1 $CdCl_2$ 1 $MgCl_2$, 1 $CaCl_2$, 10 HEPES, 10 glucose, pH 7.3; intracellular (mM): 140 CsF, 1.1 EGTA, 10 NaCl, 10 HEPES, pH 7.3.

Human donor DRG neurons: Coverslips were transferred to a recording chamber and bathed in extracellular solution (mM): 145 NaCl, 3 KCl, 2.5 $CaCl_2$, 1.2 $MgCl_2$, 10 HEPES, 7 glucose, 0.05 $CdCl_2$ adjusted to pH 7.4 with NaOH, with a flow rate of 1-2 ml/min. Borosilicate glass pipettes with the resistance of 1.5 to 2.5 MΩ were made using a P-1000 micropipette puller (Sutter Instrument, Novato, CA, USA). The intracellular solution contained (mM): 130 K-gluconate, 5 KCl, 5 NaCl, 2 $MgCl_2$, 0.3 Ethylene glycol-bis(2-aminoethylether)-NNN'N'-tetraacetic acid (EGTA), 10 HEPES, 2 Na ATP, adjusted to pH 7.3 with KOH and 294 mOsm with sucrose. Coverslips were pre-incubated in 50 μM PW164 or ZL192 or 0.1% DMSO vehicle for 1 hour before recording. Recordings were obtained on MultiClamp 700B and Digidata 1550A (Molecular Devices) and sampled

at 20 kHz. Neurons were investigated if resting membrane potential was below -45 mV. Neurons were held under current clamp, and membrane properties and action potential discharge were assessed as previously described (23).

Human Dorsal Root Ganglia Procurement

Human dorsal root ganglia (DRGs) procurement procedures were performed in accordance with the Institutional Review Boards at the University of Texas at Dallas. DRGs were procured from organ donors through a collaboration with the Southwest Transplant Alliance (STA). DRGs were frozen in powdered dry ice at the time of extraction and then stored in a -80°C freezer until use. Details on tissue procurement procedures, quality metrics, and tissue preparation can be found on protocols.io doi:10.17504/protocols.io.kqdg32qr1v25/v1 (2024). The medical history of each donor was provided by the STA and is a summary of information from hospital records and family members. Donor demographics, condition (non-diabetic vs diabetic painful neuropathy (DPN)), cause of death, and DRG levels are outlined below:

Donor demographics and DRG information.

Donor ID	Age	Sex	COD	Condition	DRG level
DN0157	68	M	CVA/Stroke	DPN	L4
DN0221	52	M	CVA/Stroke	DPN	L5
DN0229	63	M	CVA/Stroke	Non-diabetic	L4
DN0145	60	M	CVA/Stroke	Non-diabetic	L5

DPN = Diabetic painful neuropathy; CVA=cerebrovascular accident; COD = Cause of Death

Human DRG Immunofluorescence

Three 20 μm experimental tissue sections (technical replicates) and one 20 μm negative control section were cryosectioned from each human DRG onto SuperFrost Plus charged slides (Fisher Scientific; Cat# 12-550-15). Slides were then removed from the cryostat and thawed in a 37°C incubator for 1 minute, and then immediately transferred to 4% PFA/4% sucrose in 1X PBS for 15 minutes. Slides were rinsed in 1X PBS. Excess PBS was blotted off the sides of the slides with a Kim wipe, and hydrophobic boundaries were drawn around each section using a hydrophobic pen (ImmEdge PAP Pen, Vector Labs). Slides were placed into a light-protected humidity control tray and blocking solution (10% Normal Goat Serum, 0.3% Triton X-100 in 1X PBS) was pipetted onto each section until fully covered. Sections were incubated in blocking solution for 1 hour at room temperature.

Following incubation, the slides were rinsed in 1X PBS in a coplin jar, returned to the humidity control tray, and then incubated in primary antibody diluted in blocking buffer overnight at 4°C. The primary antibodies used were mouse-anti-FGF13/FHF2 (Neuromab clone N91/27 2µg/ml, Antibodies Incorporated; RRID: AB_2877474), mouse-anti-Nav1.7 (Neuromab clone N68/6 2µg/mL, Antibodies Incorporated; RRID: AB_2877500), and chicken-anti-peripherin (EnCor 1:750, Cat# CPCA-Peri; RRID: AB_2284443). Negative control sections were exposed to blocking solution in place of primary antibody. The following day, all slides were washed in 1X PBS and incubated in secondary antibodies (each at 1:2000) with DAPI (1:5000; Cayman Chemicals Cat# 14285) diluted in blocking buffer for 1 hour at room temperature in the light-protected humidity-controlled tray. The secondary antibodies used were goat-anti-chicken H&L 488 (Invitrogen, Cat# A11039), goat anti-mouse IgG2a 555 (Invitrogen, Cat# A21137), and goat anti-mouse IgG1 647 (Invitrogen, Cat# A21240). Slides were then washed in 1X PBS, and then each section was covered in the lipofuscin blocker, True Black (1:20 dilution in 70% EtOH; Biotium, Cat# 23014), for 1 minute at room temperature. The slides were then thoroughly rinsed with ultrapure water and left to dry away from light. Once dried, the slides were immediately coverslipped with Prolong Gold Antifade reagent and left to cure for 24 hours. All sections were imaged on an FV4000 confocal microscope (Evident Scientific) at 20X magnification (UPLXAPO 20X objective, Olympus). Sequential line scanning was performed using the 405nm, 488nm, 561nm, and 640nm laser wavelengths to capture DAPI, peripherin, FGF13, and Nav1.7, respectively. Image size (pixels) was 1024 x 1024, and the automatic confocal aperture setting was selected (131µm). 2-3 single plane xy images were acquired of each section, and a minimum of three sections were imaged per DRG/donor. Image settings for FGF13 and Nav1.7 were kept consistent for non-diabetic vs. DPN sample comparisons. Negative controls were imaged using the same settings. Image analysis was conducted blinded by converting each file name to a random 14-digit code using a custom script (written by Jason Faulkner, how-to-geek.com). Unblinding occurred after image analysis was completed by referencing the script's translation file. Image files were opened in Olympus CellSens (v1.18, Evident Scientific) and the soma of each neuron was traced using the ROI closed polygon tool using the peripherin-stained channel. The program automatically measured the maximum neuronal diameter and mean fluorescence intensity of the FGF13 and Nav1.7 channels of each neuronal ROI. The mean fluorescence intensity values from each section (three

images/section) were averaged for each channel for each DRG/donor. Corrected mean fluorescence intensity was calculated by subtracting the average neuronal background from the negative controls for each channel. The ratio of FGF13:Nav1.7 for each section was also calculated. Final values were then graphed in GraphPad Prism version 10.0.2 (GraphPad Software, Inc.). Statistical analysis (non-parametric t-test) was run in GraphPad, and sample sizes are indicated on the graph and/or figure captions.

Expression and purification of FGF13

To purify FGF13, the pETDuet-1-FGF13U plasmid was transformed into *E. coli* BL21 (DE3) pLys cells (Invitrogen) and purified using standard two-step purification as described here. Cells were grown at 37 °C (OD_{600} = 0.7), and protein expression was induced with 0.1 mM isopropyl thio-beta-D-galacto-pyranoside (IPTG) for 24 hours at 16 °C. Cells were harvested and lysed by sonication at 4 °C in lysis buffer containing (mM): 10 sodium phosphate (prepared from 0.5 M of Na_2HPO4 and NaH_2PO_4), 25 HEPES, 150 NaCl, 0.1 phenyl methyl sulphonyl fluoride (PMSF), 0.1% CHAPS pH 7.0. The lysate was centrifuged at 40,000xg for one hour at 4 °C. The filtered supernatant was applied to a pre-equilibrated heparin column, washed using (wash buffer (mM): 10 sodium phosphate, 25 HEPES, 150 NaCl, 0.1% CHAPS pH 7.0) and then FGF13 eluted with NaCl 0.2-2.0 M in sodium phosphate 10 mM + NaCl 0.2-2.0 M, pH 7.0 buffer. Furthermore, the concentrated FGF13 was purified on AKTA FPLC (GE Healthcare) using a Superdex 200 HiLoad 16x60 size exclusion chromatography column pre-equilibrated with 50 mM Tris, 150 mM NaCl, pH 7.5 (GE Healthcare). Eluted fractions were checked on SDS-PAGE for protein expression and concentration was determined using UV absorbance with a NanoDrop (Thermo Scientific).

Quantitative reverse transcriptase PCR (qRT-PCR)

RNA samples for real-time analysis were quantified using a Nanodrop Spectrophotometer (Thermo Scientific) cDNA was generated from total RNA (1 µg) in a 20 µL reaction using the reagents of iScript™ Reverse Transcription Supermix for RT-qPCR (Bio-Rad Laboratories cat. 1708840). *FGF13* gene expression was determined by qRT-PCR. qRT-PCR was performed in duplicate for each sample using 4 µL of cDNA in a total volume of 20 µL using the iTaq Universal SYBR Green Supermix (Bio-Rad, cat# 1725270). All RT-PCR assays

were run using the BioRad CFX384 Touch Real-Time PCR System (Bio-Rad, Hercules, CA, United States) with the following conditions: 95°C (20s), 95°C (5s), 60°C (30s) repeated by 40 cycles and 4°C. RT-PCR primers set (Bio-Rad, Hercules, CA, United States): *FGF13* (Unique Assay ID: qMmuCED0048180), *GAPDH* (Unique Assay ID: qMmuCID0018612), *18S* sense 5'-CTCAACACGGGAAACCTCAC, *18S* anti-sense 5'-CGCTCCACCAACTAAGAACG. Samples were replicated three times. The mRNA expression levels were normalized to either *GADPH* or *18S* and relative gene expression was calculated using the ΔΔCt method.

Immunocytochemistry

After treatment, coverslips containing hIPSC-derived sensory neurons were fixed with 4% PFA/4% sucrose for 15 minutes, permeabilized for 5 minutes using 0.1% Triton X-100 in PBS and blocked with 10% Normal Goat Serum (ThermoFisher Scientific) for 30 minutes. Cells were stained using primary antibodies ms IgG1 Nav1.7 (NeuroMab, Catalog # 75-103; 1:200), ms IgG2a FGF13 (Neuromabs, Catalog # 75-248; 1:200), and Rb βIII Tubulin (Abcam, catalog # ab18207; 1:2000) overnight at 4∘C in 3% BSA. Isotype specific secondary antibodies ms IgG1 Alexa 568 (Life Technologies, catalog # A-21124; 1:200), ms IgG2a Alexa 647 (Life Technologies, catalog # A-21241; 1:200), and rb Alexa 488 (Life Technologies, catalog # A-11008; 1:200) in 3% BSA were applied for 2 hours. After washing, cell membranes were labeled using CellBrite Cytoplasmic Membrane Dye (Biotium) according to manufacturer directions. After final washing, coverslips were mounted using ProLong Gold Antifade Mountant (ThermoFisher). hIPSC-derived sensory neurons were fixed at day 14 in buffered formalin (10 wt%/vol%, Fisher Scientific 23-305-510) for 10 minutes, permeabilized in DPBS plus Triton X-100 (0.2 vol%/vol%, MilliporeSigma, T8787-100ML), blocked in DPBS plus bovine serum albumin (1 wt%/vol%, MilliporeSigma A3059-100G) and Tween-20 (0.1 vol% MilliporeSigma P1379) for 2 hours, and incubated with primary antibodies overnight. Cultures were washed twice with blocking solution and incubated 1 hour with secondary antibodies. 4′,6-diamidino-2-phenylindole dilactate (DAPI, Thermo Fisher D3571) was added for 10 minutes before washing three times in DPBS. Antibodies used include: TUJ1 (1:250, MilliporeSigma MAB1637), TRPV1 (1:500, Abcam ab3487), Alexa Fluor 488 Donkey anti-Mouse (1:500, Thermo Fisher Scientific A21202), and Alexa Fluor 555 Donkey anti-Rabbit (1:500, Thermo Fisher Scientific A-31572). Negative controls included

unstained cultures, cultures stained with secondary-only antibodies, and positively stained cultures known not to express the antigens of interest. Cultures were imaged on a Biotek Cytation1 running Gen5 software.

RNAscope

RealDRG^{TM}. hIPSC-derived sensory neurons were fixed at day 14 in buffered formalin (10 wt%/vol%, Fisher Scientific 23-305-510) for 30 minutes. RNAscope in situ hybridization multiplex v2 was performed as instructed by Advanced Cell Diagnostics (ACD) for cell-based assays. The HS-TRPV1 probe (ACD 415381) was used. All tissues were checked for RNA quality using a positive control probe cocktail (ACD) and a negative control probe against the bacterial DapB gene (ACD) was used to reference nonspecific/background label. Cultures were imaged on a Biotek Cytation1 running Gen5 software. For quantification, cells were manually counted positive if there were more than 5 puncta within the soma.

Donor-derived DRG neurons. For in situ hybridization using RNAscope, whole hDRG were fixed in 4% paraformaldehyde for 24 hours followed by transfer to 30% sucrose overnight. Frozen sections were cut at 14-18 microns and then stored at -80°C. RNAscope Multiplex Fluorescent Reagent v2 Assay (Advanced Cell Diagnostics Inc.; cat # 323100) was carried out for fresh-frozen tissue as recommended by the manufacturer. The RNAscope FGF13 (873891) probe was hybridized and developed with a single channel. A second probe for SCN9A (562251-C2) was hybridized and developed in a second channel. Tyramide Signal Amplification (TSA) Plus Cyanine 3 (1:1500, PerkinElmer) and Cyanine 5 (1:1000, PerkinElmer) were each diluted with TSA buffer to visualize the FGF13 and SCN9A hybridized signals, correspondingly. Slides were counterstained with 4',6-diamidino-2-phenylindole (DAPI) and coverslipped with Prolong Gold Antifade Reagent (Invitrogen). Image acquisition was captured as stitched Z stacks using a Keyence BZ-X800E fluorescence microscope. Cells positive for *FGF13* and *SCN9A* were hand counted using ImageJ software to mark positive cells. Cells containing ten or more fluorescent puncta were considered to be positive.

In vivo studies

Animals

Experiments were performed on 6- to 12-week-old male C57BL/6 mice (The Jackson Laboratory, Bar Harbor, ME) housed in the AAALAC-accredited facility at the University of Texas Medical Branch. Mice were group-housed with up to 5 mice per cage on a 12-to-12-hour light–dark cycle and had access to water and food *ad libitum*. All protocols were approved by the Institutional Animal Care and Use Committee (IACUC) at the University of Texas Medical Branch and in accordance with the National Institutes of Health (NIH) guidelines.

Drugs injections

PW164 and ZL192 were dissolved in DMSO and saline in a ratio of 1:10. 0.1% capsaicin (10% ethanol, 10% Tween-20 in saline; Sigma-Aldrich). All the compounds were injected at the center of the hind paw. The control group received a DMSO-saline mixture. 0.1% capsaicin was injected at the center of the hind paw and then PW164 (0.07 mg/ml) or DMSO was intradermally administered at the site of capsaicin injection Capsaicin was prepared from 1% (wt/vol) capsaicin stock solution dissolved in 100% ethanol, and 0.1% of diluted stock was made using tween-80 and saline solution at a 1:1:8 ratio. Bupivacaine was prepared from a 1:2 dilution with sterile 0.9% saline and locally injected in the center of the paw at 7 mg/ml.

Intrathecal injections

AAV2 viral particles packaged by the University of North Carolina Gene Therapy Core Facility (viral titer ranging from $1 \times 10^{10.2}$ to 1×10^{13} DRP/ml) were used for intrathecal injections. Before intrathecal injections in the lumbar region, mice were maintained in an anesthetizing box with 2% isoflurane to induce unconsciousness. Six microliters of viral vector were gently injected into the CSF through a 30-gauge needle with a Hamilton syringe in the lumbar area between vertebrae L4 and L5 by gently gripping the iliac crest of the mouse and inserting the needle with an angle of 45°. The correct procedure was confirmed by the mouse's tail reflexive flick.

Assessment of mechanical sensitivity

Mechanical sensitivity was assessed using von Frey filaments (VFF 0.1g and 1.0 g force) in mice that were either naïve or received intraplantar (i.p.) injection of capsaicin. VFFs were applied at the injection site (capsaicin or drug unilateral intradermal injection at the center of the hind paw using a 30-G needle). VFF of 1.0 g force, which evokes a 40–60% withdrawal response in the naïve condition, was used to detect hyperalgesia, and VFF of 0.1 g

force, which normally does not evoke a withdrawal response, was used to detect allodynia. The extent of mechanical sensitivity was expressed as % withdrawals from 10 consecutive stimulations.

Assessment of thermal sensitivity

Plantar tests for thermal sensitivity were performed using the Hargreaves apparatus (Ugo Basile, Italy) in mice that were either naïve or received i.p. injection of capsaicin or test compound. A thermal stimulus, generated by an infrared-focused light source, invisible to the rodent, was applied through a glass pane, to the plantar surface of the hind paw of the animal. The response to this stimulus was considered the withdrawal of the stimulated paw (lifting and licking). Light intensity was set up at 30% and the cut-off at the 20 s. The final value of thermal withdrawal latency, expressed in seconds, was the average value of three consecutive trials.

Bioinformatic analysis of transcriptomic datasets

Previously published transcriptomic datasets from mouse, rat and human sensory ganglia were reanalyzed to determine the neuronal subtypes molecularly defined by expression of FGF13 alongside specific marker genes. Overall expression pattern was first assessed in bulk RNA-Seq datasets (44, 46-47). Subsequently, expression analysis was performed using data from human spatial transcriptomics (56) and mouse single-cell transcriptomics datasets (44). Processed data files were mined from supplementary files associated with the published reports.

High fat diet-induced model of diabetic neuropathy

Male C57BL/6 wild-type mice were fed either a standard chow diet (SD) or a high-fat diet (HFD) with 60%kcal fat from lard (Research Diets, Inc.# D12492) from 6 weeks of age.

Glucose Tolerance Testing

Mice were fasted for 4 hours, and baseline blood glucose was measured using an Abbott FreeStyle Freedom Lite blood glucose meter (Abbott Laboratories). Mice were administered a glucose bolus (1 g/kg body weight i.p.) and blood glucose was monitored over 2 hours.

Pharmacological testing in the HFD mouse model

PW164 was reconstituted in 10% DMSO in sterilized saline, with a final maximum dissolved concentration of 24 μg/μl. The mouse dose of PW164 used to attenuate pain was 15 μg/g body weight (bw). The volume for hindpaw

intradermal injection was calculated using the formula: (body weight * 15 µg/µl) / 24. For hindpaw intradermal (i.d.) injections, mice were briefly anesthetized with inhalation of isoflurane at a concentration of 3% in oxygen at a flow rate of 2 L/min to induce anesthesia, and 2.5% to maintain it. The PW164 solution was delivered using a 50 µl microsyringe (Hamilton 80501). The needle tip was inserted into the hindpaw glabrous dermis at an angle ≤20°, and the calculated volume of PW164 solution was delivered slowly. The formation of an obvious bump without leakage of the injected solution indicated successful injection. After drug delivery, the needle was withdrawn after a 2–3 second wait. Following the injection, animals were returned to their home cages to rest, move freely, and await the time course for the Von Frey test.

Assessment of mechanical sensitivity in the HFD mouse model

Mechanical sensitivity was assessed using von Frey filaments (VFF 0.07 g and 2.0 g force) in mice that received either vehicle (DMSO) or compound PW164 via intradermal (i.d.) injection. During von Frey testing, gentle mechanical stimulation was applied to the hindpaws to measure mechanical allodynia, expressed as the 50% hindpaw withdrawal threshold, an indicator of whether the mice developed pain. Prior to the von Frey test, mice were acclimatized to the testing conditions and environment for three consecutive days. Additionally, mice were restricted inside a transparent, ventilated chamber on top of a metal mesh stand for 1 hour each day during the acclimatization period. Mice were injected with either the vehicle or PW164 via i.d. injection under anesthetic conditions. The von Frey time course was initiated ≥1 hour post-injection. For mechanical stimulation, Von Frey filaments ranging from 2.84 (0.07 g) to 4.31 (2 g) were applied to the glabrous center of the hindpaw. Filaments were applied from beneath the mesh to the center of the mouse's hindpaw. A positive response was defined as perpendicular withdrawal or horizontal removal of the hindpaw, without causing pain or stress to the mouse. Stimulation was repeated, increasing or decreasing the filament gauge until three pairs of responsive and unresponsive recordings were obtained. The 50% hindpaw withdrawal threshold was calculated using the formula: 50% threshold (g) = $10^{(X + kd)}/10^4$. The results were expressed as the mean ± SEM and plotted using GraphPad Prism 10.

Statistical Analysis of human DRG from non-diabetic vs diabetic donors

Three sections per male donor were analyzed, and each section comprised three images. The mean fluorescence intensity values of Nav1.7 and FGF13 for all small neurons (diameter <76 um) in each image per section were averaged into 1 mean value per section. This generated 3 mean values per donor and thus, 6 mean values per condition. The 6 mean values for each protein in non-diabetic donors were compared with the 6 mean values for diabetic neuropathy donors with a non-parametric t-test (Mann-Whitney U test). Additionally, the ratio of FGF13 to Nav1.7 was also determined and compared between non-diabetic vs diabetic donors.

Genetic analysis

Study population

FGF13 and *SCN9A* genes were investigated in patients with chronic pain to identify rare genetic variants, with a potential impact at the protein level or on gene expression. We analyzed two cohorts of patient genetic data available at Fondazione IRCCS Istituto Neurologico Carlo Besta, namely the "Besta cohort" and the "PROPANE study cohort". The Besta cohort is constituted by 781 outpatients reporting symptoms of chronic pain, recruited according to previously published criteria (59). The PROPANE cohort is composed by 1191 subjects, including 230 patients with painful diabetic neuropathy (PDN), 317 painless diabetic neuropathy (PLDN), and 644 painful small fiber neuropathy (PSFN) (60). Collectively, the analyzed group of patients with chronic non-diabetic pain was 1425 (781 from Besta; 644 SFN from PROPANE). We included in the study the genetic analysis of 216 healthy controls, aged more than fifty years old, in order to recognize rare variants carried by subjects who didn't develop any chronic pain condition, thus with a predicted minor impact.

Sequencing approach

Two different approaches were used for the library preparation, to capture all exons and intron-exon junctions (±20 bp) of 107 pain-related target genes, including SCN9A and FGF13. The Besta cohort samples were all tested by Illumina DNA Prep with Enrichment (Illumina Inc.) and sequenced on MiSeq System with 2×150-bp paired-end reads (Illumina, Inc., San Diego, CA, USA). Sequenced data were analyzed according to in-house pipeline of quality check, variant calling, and annotation (59). The PROPANE Study cohort libraries were obtained

through smMIPs-NGS (single molecule Molecular Inversion Probes-Next Generation Sequencing) enrichment and sequenced using an Illumina NextSeq500 System with 2×150-bp paired-end reads (Illumina, Inc.). Sequenced data were analyzed by using an in-house smMIPs-NGS data analysis pipeline. Variants that did not pass the variant filtering (total reads count ≤20, alternative allele depth ≤10, and allele balance of 25%) were removed. Captured on-target regions were compared with the reference sequence GRCh37.

Variants selection and annotation

Rare genetic variants have been selected according to the following criteria: rare in the general population (minor allele frequency <1% in GnomAD_total), having a functional impact on the coding sequence (non-synonymous and splicing mutations), localizing in the coding region of *FGF13* and in the CTD of *SCN9A*. Only C-terminal region was analyzed in this context, given its involvement in the molecular interaction between the encoded proteins FGF13 and Nav1.7. Variants detected were annotated according to the guidelines of the Human Genome Variation Society (https://varnomen.hgvs.org/), and classified referring to ACMG recommendations (*54*) using Alamut Visual Plus (SophiaGenetics). The position of nucleotide substitutions (c.pos) in *SCN9A* gene has been annotated on transcript NM_001365536.1, and *FGF13* on transcript NM_004114.5. For mutations included in the ClinVar database (Build 156, Release September 21, 2022), the ID number and the interpretation of pathogenicity are reported.

Figs. S1 to S17

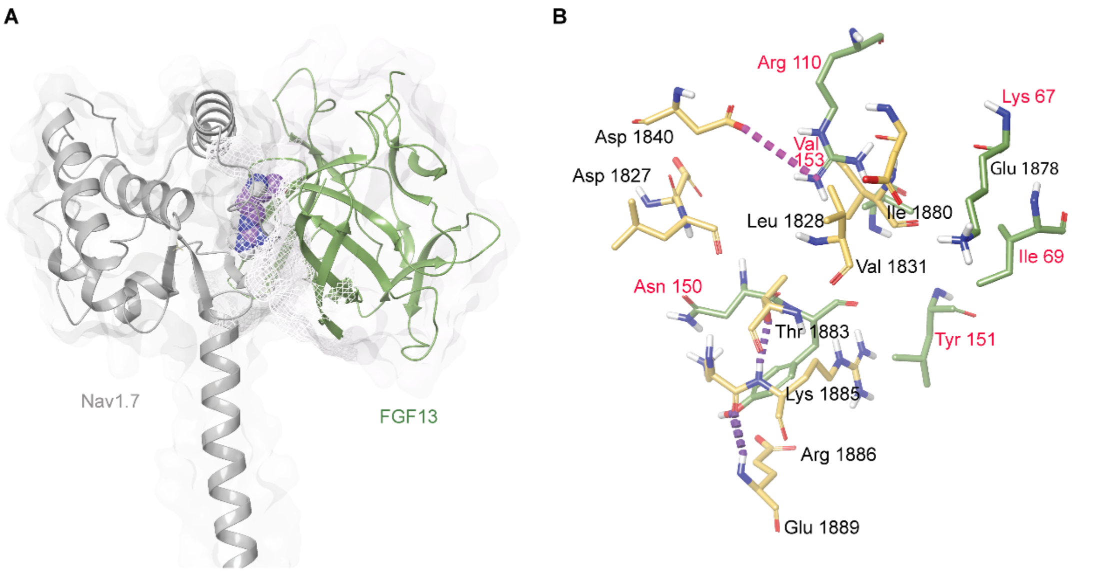

C

Hot spot residues at the interface of the FGF13/Nav1.7 complex and their interaction analysis

FGF13 (green)	Nav1.7 CTD (gray)	Interactions
Tyr151	Glu1889	H-bond
Tyr151, Leu195	Arg1886	H-bond, π cation, hydrophobic
Asn150, Tyr151	Lys1885	H-bond, hydrophobic
Asn150	Thr1883	H-bond
Asn150	Asp1827	H-bond
Asn150	Leu1828	H-bond
Val153	Ile1880	hydrophobic
Ile69	Thr1881	hydrophobic
Arg110	Asp1840	H-bond, salt bridge
Arg110	Val1831	Hydrophobic
Lys67, Arg110	Glu1878	H-bond, salt bridge, hydrophobic

Colors refer to the homology model as shown in panel “B”

Supplemental Figure 1. The PPI interface of the FGF13/Nav1.7 CTD complex includes the hot spot Arg110. (**A**) Homology model of the FGF13/Nav1.7 complex using FGF13/CaM/Nav1.5 (4DCK) as a template; FGF13 is shown in green ribbon, and the Nav1.7 CTD is shown in light grey. The binding site is represented as the white mesh and Arg 110 orientation depicted in CPK. The predicted binding pocket at the protein complex interface was defined by the Site Map algorithm. The binding pocket from the complex was then superimposed to the FGF13 crystal structure (3HBW) and used for docking studies. (**B**) Hot spot residues at the FGF13/Nav1.7 interface are shown as yellow sticks for Nav1.7 CTD and green sticks for FGF13. H-bonds are shown as purple dotted lines. (**C**) Hot spot residues at the interaction interface with a list of interactions.

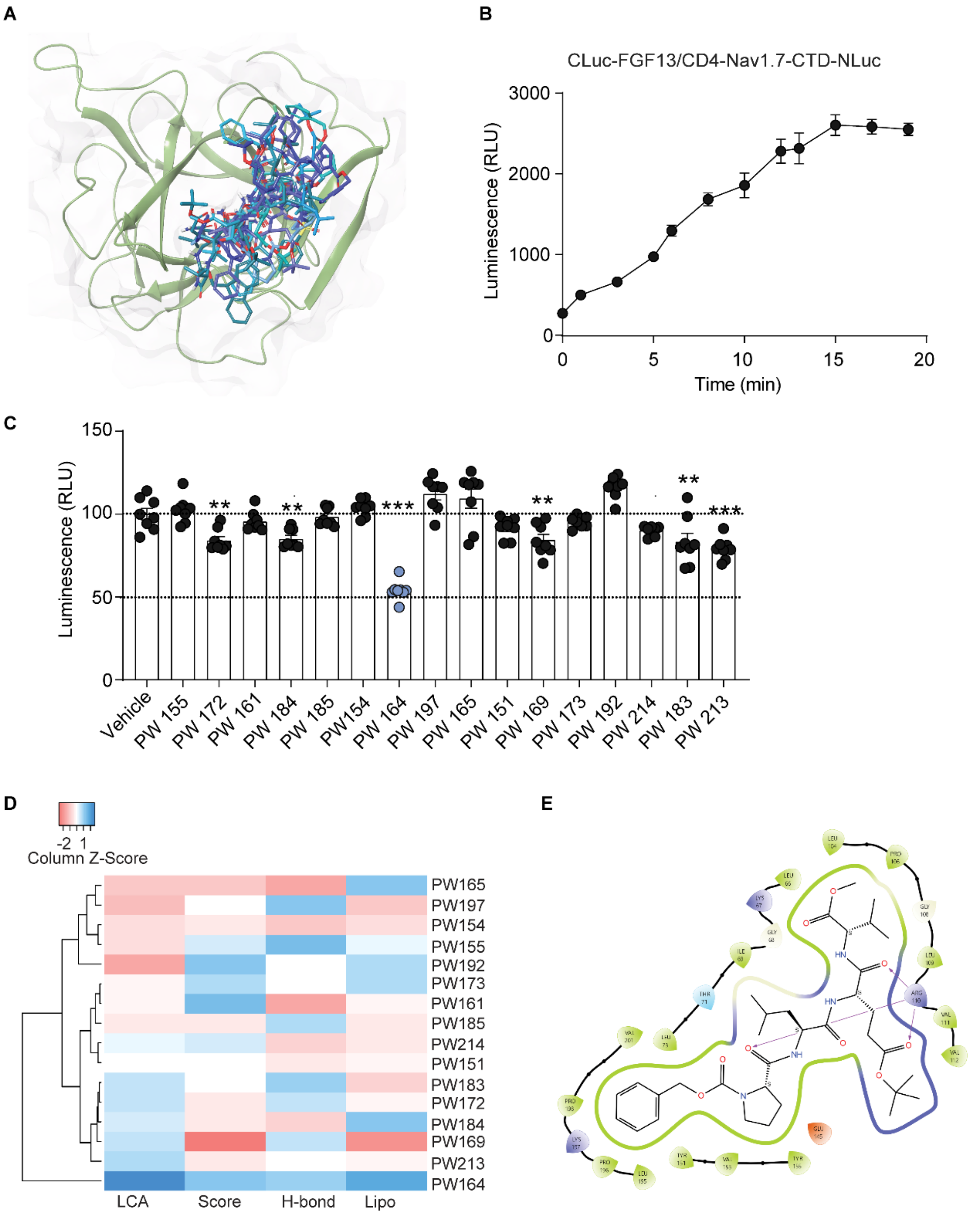

Supplemental Figure 2. In silico screening and in-cell selection identify the FGF13 negative modulator PW164. (**A**) Representative set of docking poses from the PW-series screening is shown on FGF13 binding site. FGF13 is shown as green ribbons. (**B**) Split-luciferase complementation assay (LCA) of CLuc-FGF13/CD4-Nav1.7-CTD-NLuc complex; the D-luciferase substrate is added to the reaction at time zero and luminescence signal (relative luminescence unit) reflecting FGF13/Nav1.7 CTD complex formation is shown over the course of 20 min. (**C**) Bar graph summarizing the LCA screening of the PW-series compounds (50 µM) against the FGF13/Nav1.7 complex; % luminescence signal is normalized to per-plate vehicle control wells (0.5% DMSO). (**D**) Heat map depicting docking score, H-bond coefficient, and lipophilic terms derived from Glide properties and one the LCA signal of PW-series compounds. The colorimetric scale refers to each column Z-score ranging from red to blue. PW164 was selected as hit based on in-cell and in silico performance. (**E**) 2D interaction map of PW164 with FGF13 showing H-bond interactions (purple arrow) with Arg110 as well as hydrophobic interactions between the PW164 benzyl group and FGF13 residues Val153, Leu195, Pro196, Pro198 and Val201.

Data represented as mean ± SEM; ** $p<0.01$, *** $p<0.001$, one-way ANOVA with post hoc Tukey's multiple comparisons test.

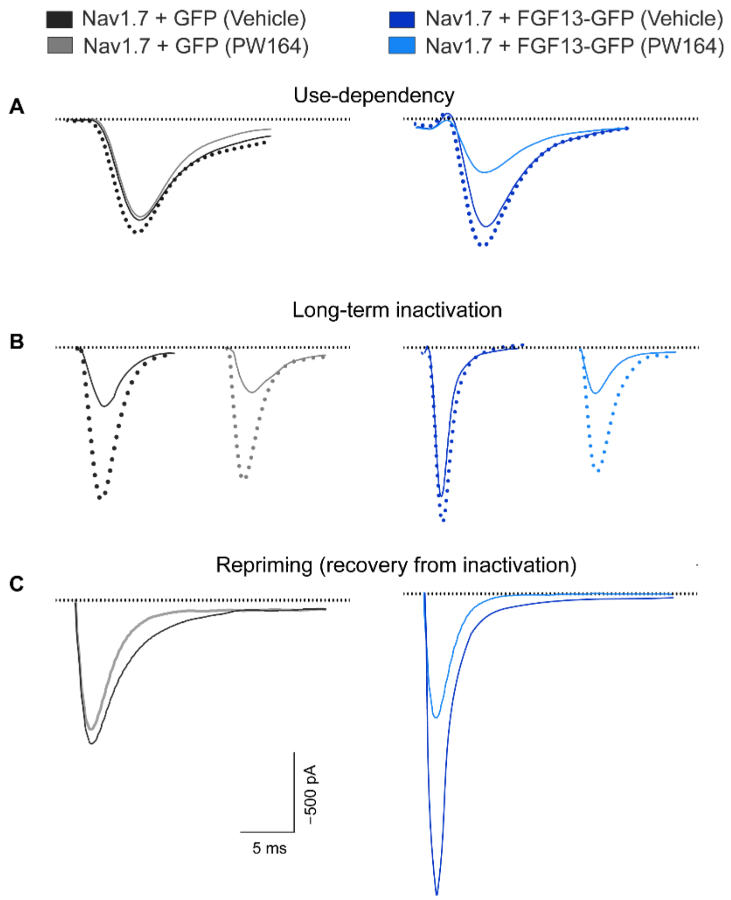

Supplemental Figure 3. PW164 modulates use-dependency and long-term inactivation of Nav1.7 currents in an FGF13-dependent manner. (**A**) Representative traces of use-dependency in color-coded experimental groups. (**B**) Representative traces of long-term inactivation in color-coded experimental groups. (**C**) Representative traces of repriming in color-coded experimental groups. Dotted line represents the first step with respect to that trace for both use-dependency and long-term inactivation.

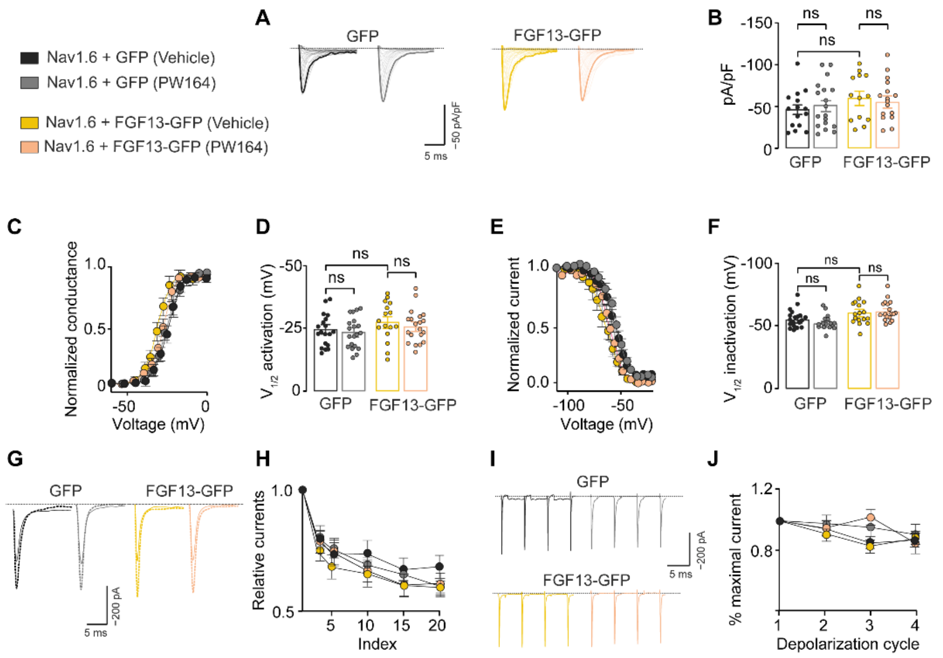

Supplemental Figure 4. PW164 does not modulate Nav1.6-mediated currents. (**A,B**) Representative traces of I_{Na} recorded from HEK-Nav1.6 cells transiently transfected with either GFP or FGF13-GFP in response to depolarizing voltage steps, and relative bar graph of peak I_{Na} density at -10 mV (n = 14-20 cells/group). (**C,D**) Normalized conductance as a function of voltage; bar graph of voltage-dependence of activation from experimental groups (n = 14-19 cells/group) described in A. (**E,F**) Normalized current as a function of voltage (n = 14-19 cells/group); bar graph of voltage-dependence of steady-state inactivation for experimental groups described in A. (**G,H**) Representative traces of I_{Na} recorded before (1st pulse) and after (20th pulse) a series of repetitive stimulations at -10 mV (dotted line-1st pulse); plot of use dependency recorded at 10 Hz. Scale bar is 5 ms, 200 pA. (**I,J**) Representative traces and relative plots of Nav1.6 channels entering long-term inactivation (LTI) for the experimental groups described in A. Dotted line represents the first step with respect to that trace for both use-dependency and long-term inactivation. Data represented as mean ± SEM; ns = not significant; one-way ANOVA with post hoc Tukey's multiple comparisons test.

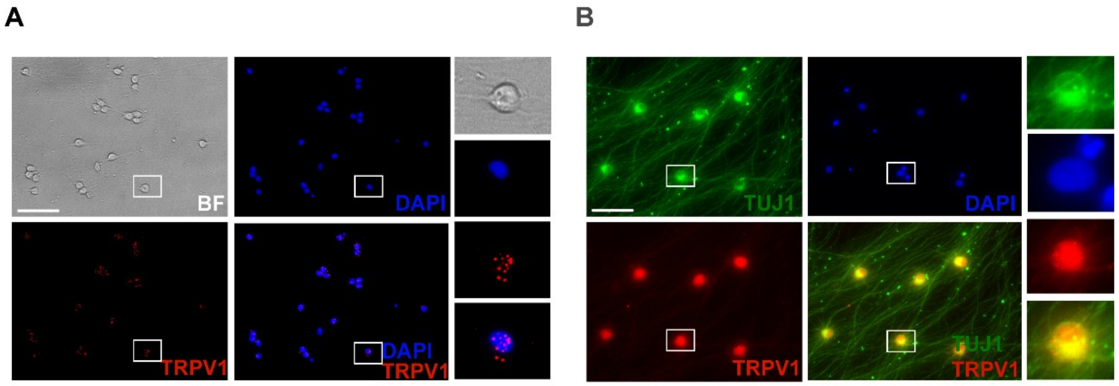

Supplemental Figure 5. TRPV1 is expressed in RealDRG™ hIPSC-derived sensory neurons. (**A**) Representative TRPV1 expression via RNAscope in hIPSC-derived sensory neurons: Brightfield image and DAPI stain (top), TRPV1 expression and Overlay (bottom), and corresponding zoomed view (right), Scale bar 100 µm. (**B**) Representative TRPV1 expression via immunocytochemistry in hIPSC-derived sensory neurons: field view of TUJ1 expression and TRPV1 expression (top), DAPI stain and Overlay (bottom), and corresponding zoomed view (right), scale bar 100 µm.

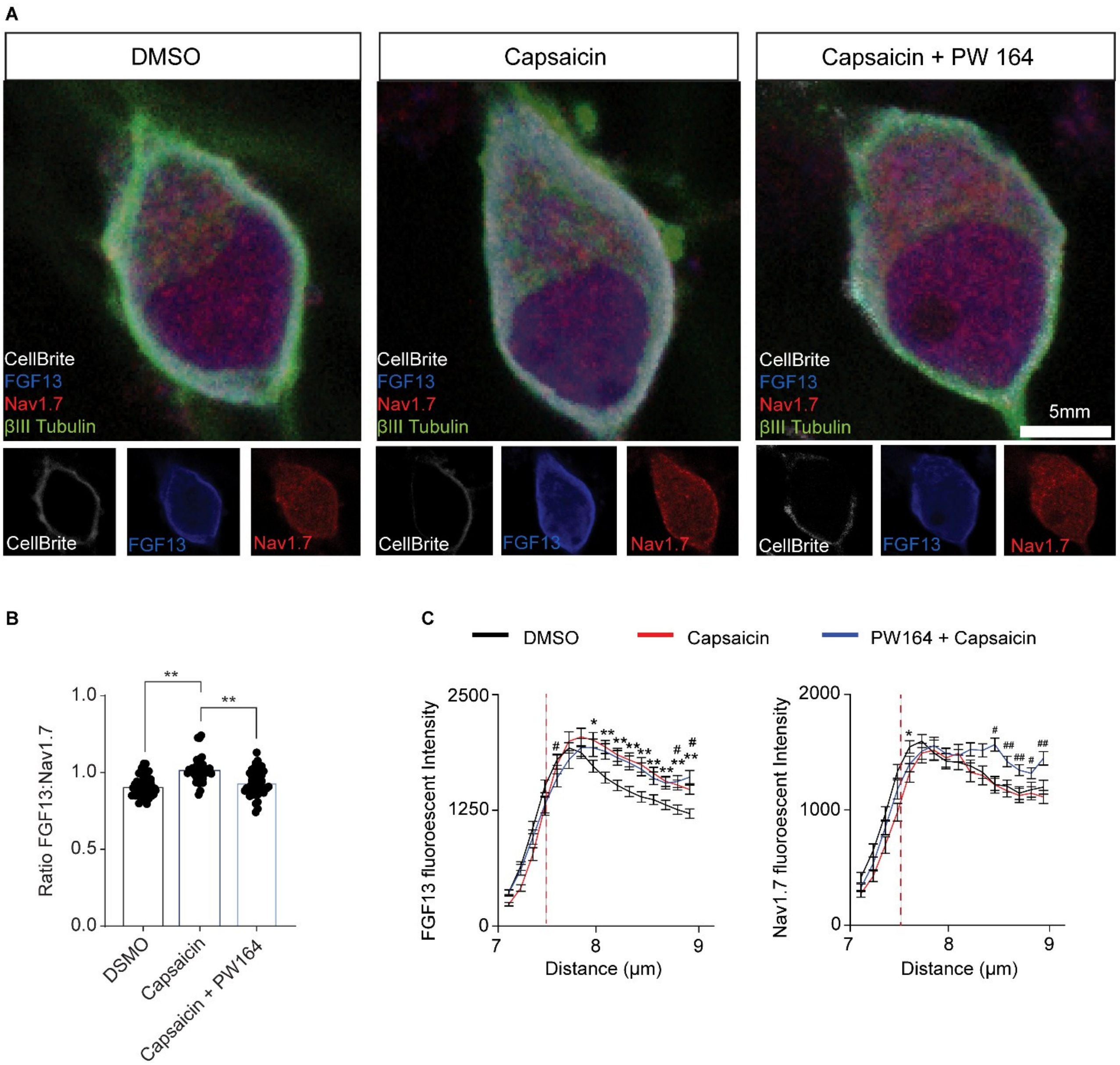

Supplemental Figure 6. PW164 reverses capsaicin-induced increases in FGF13 and Nav1.7 colocalization in RealDRG[TM] hIPSC-derived sensory neurons. (**A**) Merge of membrane marker (CellBrite, white), FGF13 (blue), Nav1.7 (red), and bIII tubulin (green) in DRG treated with DMSO, Capsaicin, or Capsaicin + PW164, with individual channels below. Scale bar is 5um. (**B**) Treatment with capsaicin increases the ratio of FGF13:Nav1.7 fluorescent intensity in the cytoplasm of treated cells, an effect rescued by co-treatment with PW164. (**C**) Profile of FGF13 and Nav1.7 from membrane and cytoplasm adjacent to the membrane, red dotted lines depicted the membrane and cytoplasm boundaries. ** indicates $p<0.01$ by Kruskal-Wallis test with Dunnet's multiple comparisons test (B); * indicates $p<0.05$ and ** indicates $p<0.01$ (capsaicin vs DMSO), or # indicates $p<0.05$ and ## $p<0.01$ (capsaicin + PW164 vs DMSO) by two-way repeated-measure ANOVA on log-normalized data with Dunnett's multiple comparisons test.

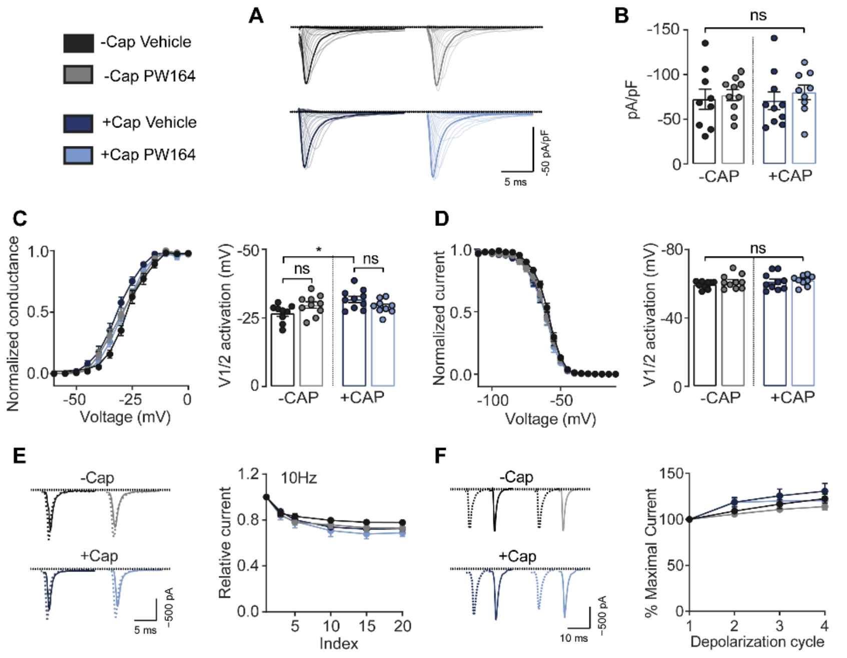

Supplemental Figure 7. PW164 does not have any effect on capsaicin-potentiated Na+ currents in the presence of Nav1.7 blocker ProTx-II. (**A**) Representative traces of I_{Na} recorded from RealDRG^{TM} hIPSC-derived sensory neurons. Scale bar is 5 ms, 75 pA/pF. (**B**) Bar graph of peak I_{Na} density for the experimental groups (n = 8-11 cells/group) described in A. (**C**) Normalized conductance as a function of voltage; bar graph of $V_{1/2}$ of voltage-dependence of activation for experimental groups (n = 8-11 cells/group) described in A. (**D**) Normalized current as a function of voltage (n = 8-11 cells/group); bar graph of $V_{1/2}$ of voltage-dependence of steady-state inactivation for experimental groups described in A. (**E**) Representative traces of I_{Na} recorded before (first pulse) and after (20th pulse) a series or repetitive stimulations at -10 mV (dotted line-1st pulse); plot of use dependency recorded at various frequencies of 20th pulse. Scale bar is 5 ms, 500 pA. (**F**) Representative traces of putative Nav1.8-mediated channels entering long-term inactivation (LTI); characterization of the fraction of Nav1.8 channels entering LTI for the experimental groups described in A. All the protocols were recorded in the presence of Nav1.7 blocker (ProTx-II 10 nM). Scale bar is 5 ms, 500 pA. Data represented as mean ± SEM; ns = not significant, * $p<0.05$; one-way ANOVA with post hoc Tukey's multiple comparisons test.

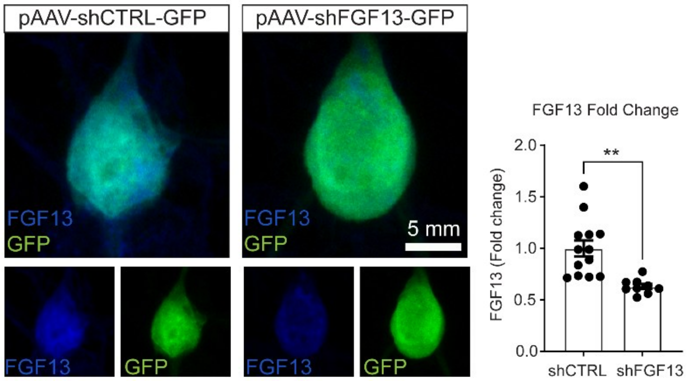

Supplemental Figure 8. pAAV-shFGF13-GFP expression reduces FGF13 protein in RealDRG^{TM} hIPSC-derived sensory neurons. Representative confocal images of RealDRG^{TM} hIPSC-derived sensory neurons expressing pAAV-shCTRL-GFP vs pAAV-shFGF13-GFP stained with an anti-FGF13 antibody (blue). The GFP signal is depicted in green. Summary bar graph of fold FGF13 change upon *FGF13* mRNA silencing. ** $p<0.01$; Student's t-test, scale bar 5 μm.

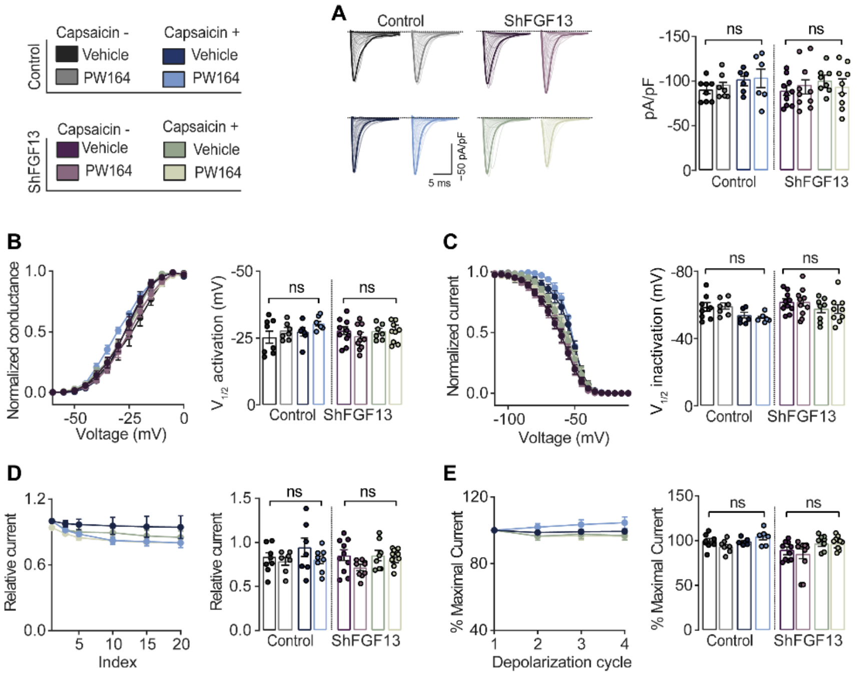

Supplemental Figure 9. Neither capsaicin nor PW164 regulate Na+ currents in RealDRG™ hIPSC-derived sensory neurons in the presence of Nav1.7 blocker ProTx-II. (**A**) Color coded scheme of experimental groups of RealDRG™ hIPSC-derived sensory neurons expressing pAAV-control or pAAV-shFGF13 in the absence or presence of capsaicin and treated with either vehicle (0.1% DMSO) or PW164 (50 µM). Representative traces of I_{Na} along with bar graphs of peak I_{Na} density (right; n = 8-11 cells/group). Scale bars correspond to 5 ms, 25 pA/pF. (**B**) Normalized conductance as a function of voltage and corresponding bar graph (right). (**C**) Normalized current as a function of voltage and corresponding bar graph from experimental groups described in A (n = 8-11 cells/group). (**D**) Plots of I_{Na} recorded before (first pulse) and after (20th pulse) a series or repetitive stimulations at -10 mV, 10 Hz and corresponding bar graph (n = 8-11 cells/group). (**E**) Characterization of the fraction of Nav channels entering LTI (left) and corresponding bar graph (right, n = 8-11 cells/group). All the protocols were recorded in the presence of Nav1.7 blocker (ProTx-II 10 nM). Data represented as mean ± SEM; ns = not significant, one-way ANOVA with post-hoc Tukey's multiple comparisons test.

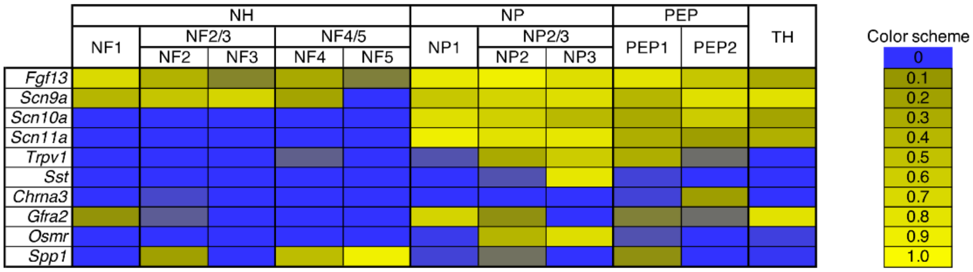

Supplemental Figure 10. FGF13 (*Fgf13*) and Nav1.7 (*Scn9a*) transcripts are expressed in molecularly defined neuronal subtypes. In mouse DRG single-cell transcriptomics derived from previously published investigations (*45*) *Fgf13* was similarly ubiquitous, with the highest expression in the non-peptidergic (NP) populations, and the lowest expression in large neurons expressing neurofilament proteins (NF populations) and/or osteopontin (Spp1). *Fgf13* and *Scn9a* show similar enrichment. Peptidergic nociceptors (PEP1), tyrosine hydroxylase (TH).

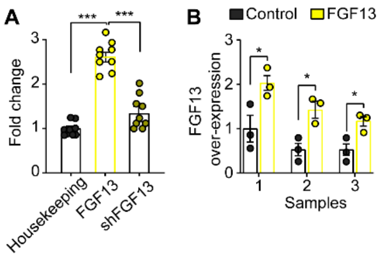

Supplemental Figure 11. In vivo FGF13 (*Fgf13*) silencing and overexpression is analyzed by qRT-PCR. (**A**) Fold change in *Fgf13* mRNA level in surgically isolated L4-L5 mouse DRG neurons following intrathecal delivery of AAV2-shFGF13-GFP (gold) or AAVs-shCTRL-GFP (yellow). The housekeeping gene is 18S (black). (**B**) Fold change in *Fgf13* mRNA level in surgically isolated mouse DRG neurons that overexpressing FGF13 (n=3). The experimental groups data were normalized with housekeeping mRNA expression. Data represented as mean ± SEM; * $p<0.05$, *** $p<0.001$; Student's t-test.

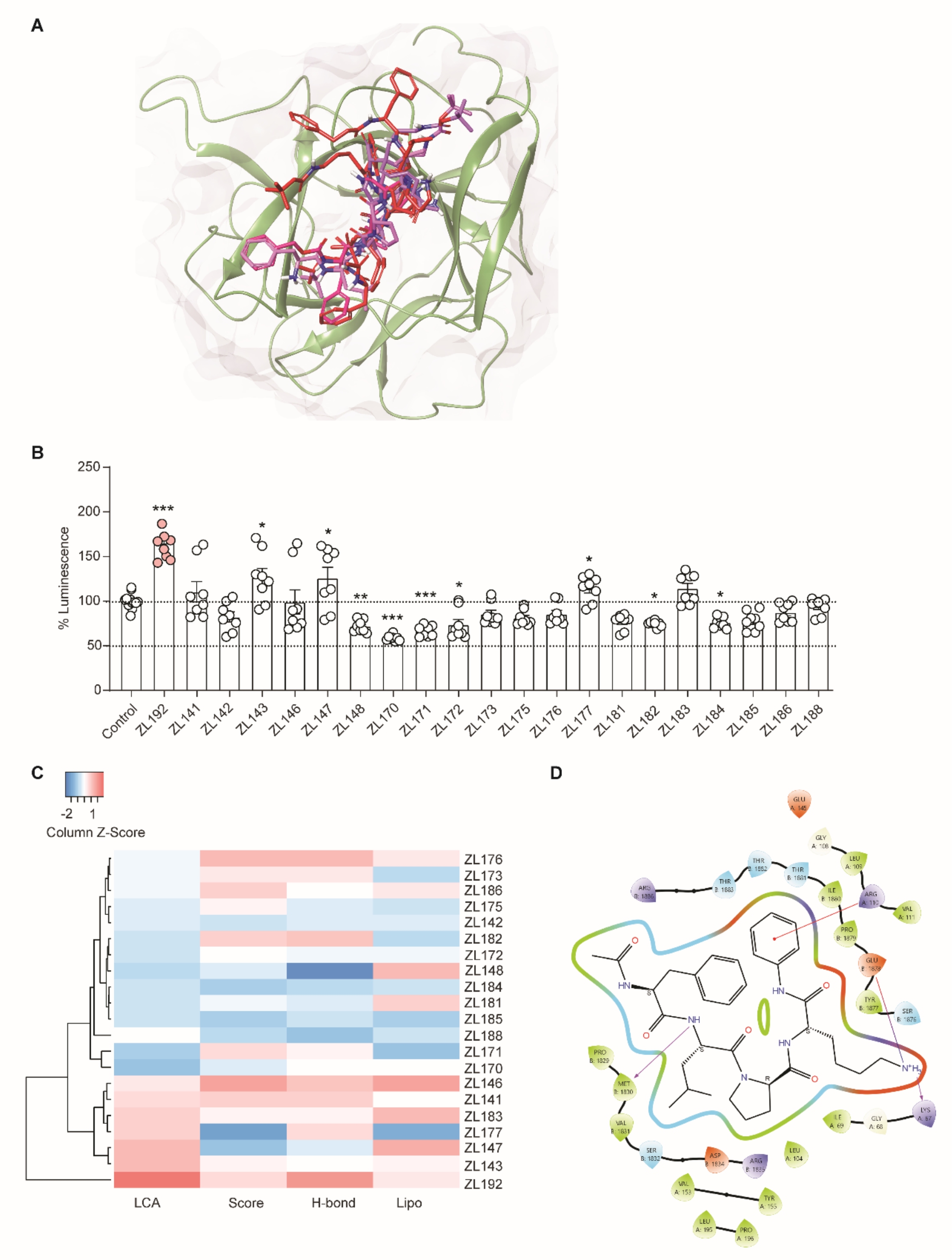

Supplemental Figure 12. In silico and in-cell screening identify the FGF13 positive modulator ZL192. (**A**) Representative set of docking pose ZL-series screening is shown on the surface of the FGF13 binding site. FGF13 is shown as green ribbons, and the interactions are shown as purple dotted lines. (**B**) Bar graph showing the screening of ZL-series compounds at one single concentration of 50 μM. Luminescence responses are normalized to per-plate control wells treated with 0.5% DMSO (light grey). (**C**) Heat map depicting docking score, H-bond coefficient, and lipophilic terms derived from Glide properties and one the LCA signal of ZL-series compounds. The colorimetric scale refers to each column Z-score ranging from red to blue. ZL192 was selected as hit based on in-cell and in silico performance. (**D**) 2D interaction map of ZL192 with FGF13/Nav1.7 CTD showing π-

cation interaction with Arg110 (red arrow) and H-bond with Lys67 (purple arrow) on FGF13; H-bond (purple arrow) and salt bridge interaction (red-blue gradient line) with Met 1830 and Glu1878 on the Nav1.7 CTD, respectively. Data represented as mean ± SEM; ** $p<0.01$, *** $p<0.001$, one-way ANOVA with post hoc Tukey's multiple comparisons test.

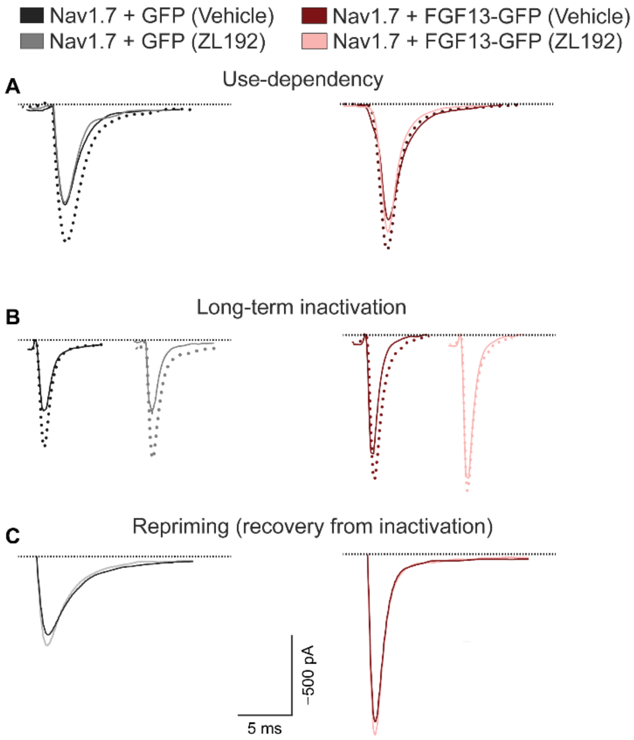

Supplemental Figure 13. ZL192 modulates use-dependency and long-term inactivation of Nav1.7 currents in an FGF13-dependent manner. (**A**) Representative traces of use-dependency in color-coded experimental groups. (**B**) Representative traces of long-term inactivation in color-coded experimental groups. (**C**) Representative traces of repriming in color-coded experimental groups. Dotted line represents the first step with respect to that particular trace for both use-dependency and long-term inactivation.

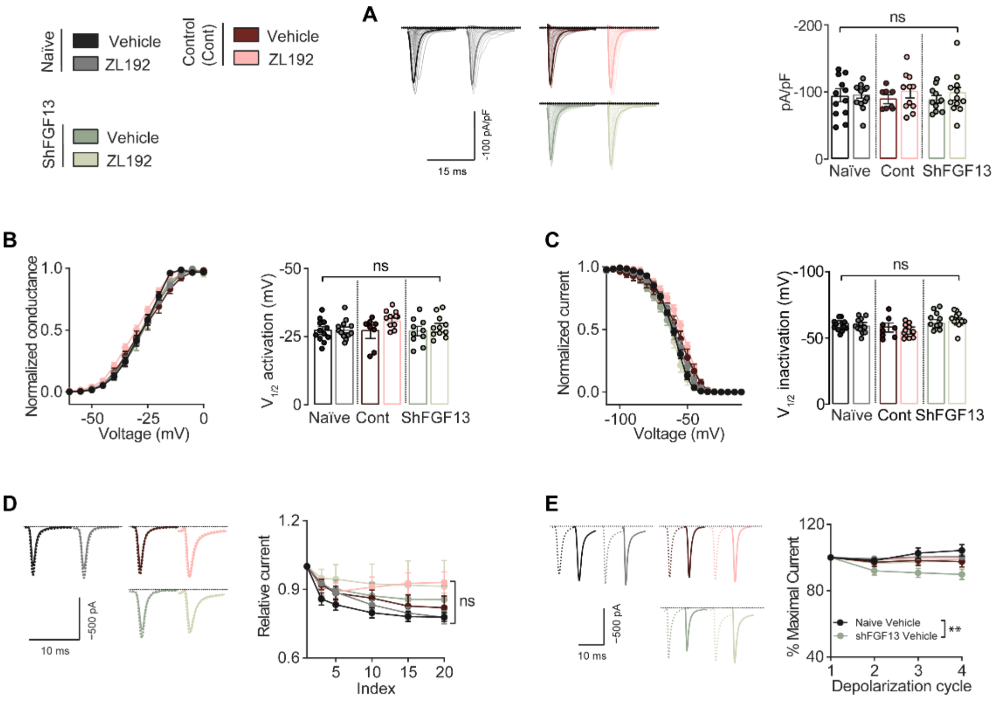

Supplemental Figure 14. ZL192 has no activity on Na+ currents recorded in RealDRG^{TM} hIPSC-derived sensory neurons in the presence of Nav1.7 blocker ProTx-II. (**A**) Color-coded scheme of experimental groups including neurons expressing pAAV-control or pAAV-shFGF13 in the absence or presence of capsaicin and treated with either vehicle (0.1% DMSO) or ZL192 (50 µM). Representative traces of I_{Na} in response to depolarizing voltage steps (left) along with corresponding bar graphs of peak I_{Na} density (right). Scale bars correspond to 15 ms, 100 pA/pF. (**B**) Normalized conductance as a function of voltage and corresponding bar graph (right) from experimental groups described in A. (**C**) Normalized current as a function of voltage and corresponding bar graph from experimental groups described in A. (**D**) Plots of I_{Na} from the indicated experimental groups recorded before (first pulse) and after (20th pulse) a series or repetitive stimulations at -10 mV and corresponding bar graph (bottom). Scale bar is 10 ms, 500 pA. (**E**) Characterization of the fraction of Nav channels entering LTI (top) in cells and corresponding bar graph (bottom). Scale bar is 15 ms, 500 pA. All the protocols were recorded in the presence of a Nav1.7 blocker (ProTx-II 10 nM). Dotted line represented the first step with respect to that trace for both use-dependency and long-term inactivation. For every experiment, n = 8-11 cells/group. Data represented as mean ± SEM; ns = not significant, one-way ANOVA with post-hoc Tukey’s multiple comparisons test.

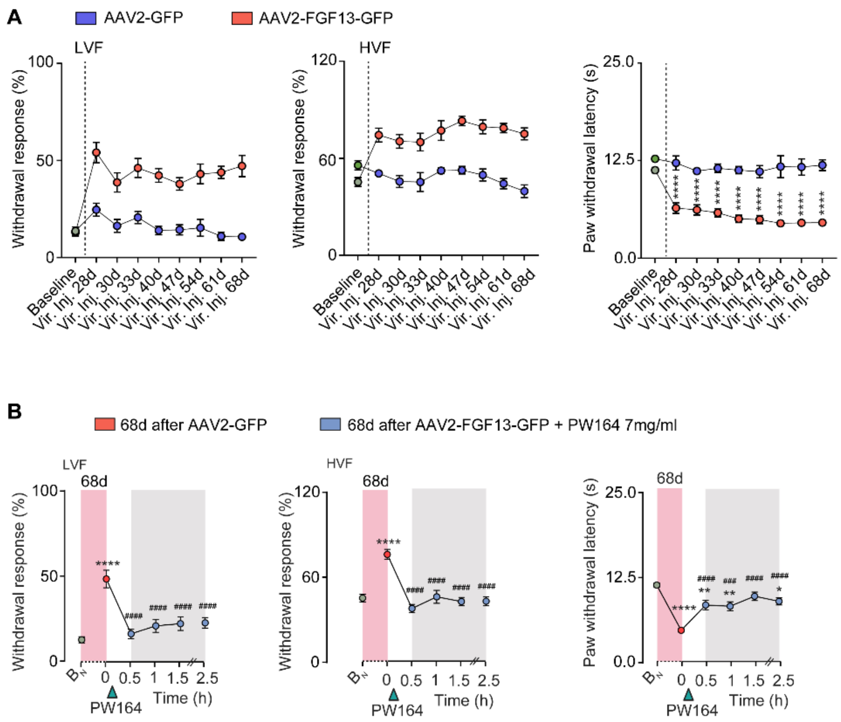

Supplemental Figure 15. FGF13 overexpression leads to persistent mechanical and thermal hypersensitivity. (**A**) Time-course of mechanical and thermal hypersensitivity induced by L4-L5 intrathecal injection of AAV2-FGF13-GFP; paw withdrawal responses at a low von Frey filament (LVF) (left) and high von Frey filament (HVF) (middle) and paw withdrawal latency (right) were measured starting 28 days after intrathecal injection for 40 days (day 68). A separate group of mice were injected with AAV2-GFP particles. Each point represents the average and the SEM from 3 trials. Each trial is the percentage of the frequency of 10 paw withdrawal responses (n = 12-8 mice/group). Each point of thermal hypersensitivity (right) represents the average with the SEM of 3 trials. For each trial the time of the paw withdrawal response (seconds) was recorded setting the Hargreaves's Apparatus with infrared intensity (IR) 30% and cutoff 20s. Data were analyzed using Two-Way ANOVA with Dunnett's multiple comparisons test; *p< 0.05, ** p< 0.01, ***p<0.001, ****p<0.0001 represents the significance vs baseline time point. (**B**) Time course of mechanical and thermal hypersensitivity and pharmacological responses of chronic nociplastic pain to intraplantar injection of PW164 (7 mg/ml). Vir. Inj.=Viral injection. Data were analyzed using One-Way ANOVA with Turkey's multiple comparisons tests; *p< 0.05, ** p< 0.01, ***p<0.001, ****p<0.0001 represents the significance vs baseline time point, while #p< 0.05, ## p< 0.01, ###p<0.001, ####p<0.0001 represents the significance vs time point 0 (viral injection day 40).

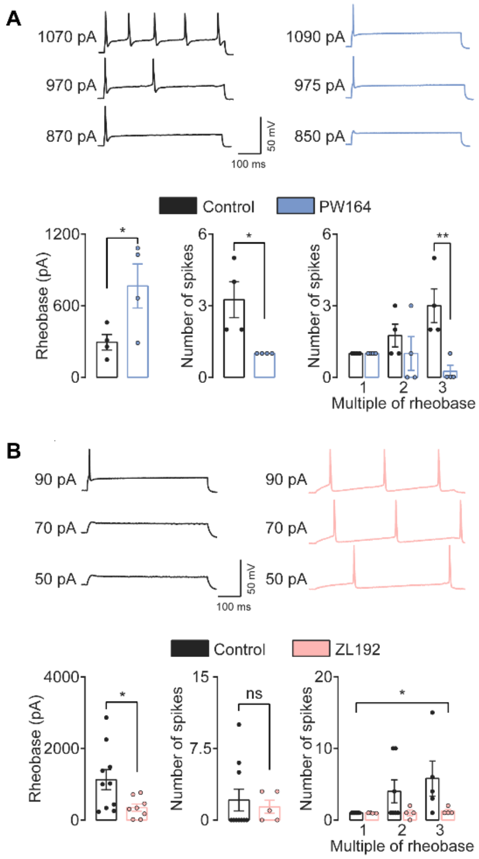

Supplemental Figure 16. FGF13 is a translationally relevant target in donor-derived DRG neurons. (**A**) Representative current-clamp recordings of action potentials from neurons in control (0.1% DMSO; grey) or treated with PW164 (50 µM; blue). Rheobase at first action potential in vehicle-treated vs PW164-treated neurons (left), number of action potentials in response to a stimulus of ~1000 pA (middle), number of action potentials evoked with an increase in multiple of rheobase (right). (**B**) Representative current clamp recordings of action potentials from neurons in control (0.1% DMSO; grey) or treated with ZL192 (50 µM; pink) and plots corresponding to panel A. The right panel indicates the main effect for the two-way ANOVA (multiple rheobase). Data represented as mean ± SEM, ns = not significant, *$p<0.05$, **$p<0.01$; n = 4 neurons, N=1 donor per recording conditions. Student's t-test with or without Welch correction and two-way ANOVA with Sidak’s multiple comparisons test.

Supplemental Tables 1-11

Supplemental Table 1. Functional characterization of PW164 in HEK293 cells stably expressing Nav1.7.

Condition	Peak density	Activation	Steady state inactivation	Tau (τ)
	pA/pF	mV	mV	ms
GFP + vehicle	−60.3 ± 6.8 (12)	−27.2 ± 0.7 (12)	−82.6 ± 1.9 (11)	1.55 ± 0.15 (10)
GFP + PW164	−69.2 ± 7.5 (11)$^{ns\#}$	−26.1 ± 0.4 (11)$^{ns\#}$	−83.5 ± 1.7 (9)$^{ns\#}$	1.48 ± 0.25 (11)$^{ns\#}$
FGF13-GFP + vehicle	−102.6 ± 18.0 (12)$^{a\#}$	−23.7 ± 0.8 (13)$^{d\#}$	−72.2 ± 1.9 (11)$^{g\#}$	1.03 ± 0.07 (10)$^{j\#}$
FGF13-GFP + PW164	−26.7 ± 3.6 (14)$^{b\$,c€}$	−19.7 ± 0.9 (17)$^{e\$,f€}$	−64.7 ± 2.1 (13)$^{h\$,i€}$	0.80 ± 0.03 (11)$^{k\#}$

Long-term inactivation (% maximal Na^+ current)			
**Condition**	2nd Pulse	3rd Pulse	4th Pulse
GFP + vehicle	−63.72 ± 4.30 (11)	−59.34 ± 5.34 (11)	−61.99 ± 5.63 (11)
GFP + PW164	−67.94 ± 3.77 (8)$^{ns\#}$	−62.35 ± 5.35 (8)$^{ns\#}$	−60.93 ± 3.90 (8)$^{ns\#}$
FGF13-GFP + vehicle	−89.11 ± 2.82 (17)$^{l\#}$	−84.66 ± 2.55 (17)$^{n\#}$	−82.64 ± 3.01 (17)$^{p\#}$
FGF13-GFP + PW164	−69.72 ± 5.21 (17)$^{m\$}$	−60.33 ± 5.60 (17)$^{o\$}$	−59.34 ± 4.68 (17)$^{q\$}$

Data are mean ± SEM; ns = not significant; one way ANOVA Tukey's multiple comparisons test.

Overall one-way ANOVA: peak current density (p<0.0001, $F_{3,45}$); V½ activation (p<0.0008, $F_{3,48}$); V½ steady-state inactivation (p<0.0001, $F_{3,40}$); tau (p<0.0130, $F_{3,43}$); long-term inactivation (p<0.0447, 0.0469, 0.0290, $F_{3,42}$)

Tukey's multiple comparisons were calculated against the following groups:

$^{\#}$=Nav1.7-GFP + vehicle
$^{\$}$=FGF13-GFP + vehicle
$^{€}$=Nav1.7-GFP + PW164

$^{a\#}p = 0.0290$, $^{b\$}p = 0.0001$, $^{c€}p < 0.0254$, $^{d\#}p = 0.0211$, $^{e\$}p = 0.0028$, $^{f€}p = 0.0001$, $^{g\#}p = 0.0038$, $^{h\$}p = 0.042$, $^{i€}p = 0.0001$, $^{j\#}p = 0.0050$ (Student t-test), $^{k\#}P = 0.0500$, $^{l\#}p = 0.0059$, $^{m\$}p = 0.0394$, $^{n\#}p = 0.0058$, $^{o\$}p = 0.0013$, $^{p\#}p = 0.0427$, $^{q\$}p = 0.0019$.

Supplemental Table 2. Functional characterization of PW164 in HEK293 cells stably expressing Nav1.7 and transiently expressing FGF13-1a.

Condition	Peak density	Activation	Steady state inactivation	Tau (τ)
	pA/pF	mV	mV	ms
FGF13A GFP + vehicle	−109.2 ± 15.0 (11)	−29.26 ± 1.05 (11)	−63.14 ± 3.06 (13)	1.52 ± 0.11 (13)
FGF13A GFP + PW164	−109.1 ± 11.7 (12)[a#ns]	−26.91 ± 1.25 (12)[b#ns]	−63.11 ± 2.14 (12)[c#ns]	1.41 ± 0.06 (10)[d#ns]
**Long-term inactivation (% maximal Na^+ current)**				
**Condition**		2nd Pulse	3rd Pulse	4th Pulse
FGF13A-GFP + vehicle		−48.22 ± 4.31 (13)[ns#]	−36.13 ± 3.68 (13)[ns#]	−30.08 ± 2.85 (13)[ns#]
FGF13A-GFP + PW164		−47.28 ± 4.68 (11)[e$ns]	−35.27 ± 3.04 (11)[f$ns]	−29.22 ± 4.16 (11)[g$ns]

Data are mean ± SEM; ns = not significant; Student t-test.

Student t-test was calculated against the following group:
[#]=FGF13.1A GFP + vehicle
$^{a\#}p = 0.9986$, $^{b\#}p = 0.1384$, $^{c\#}p = 0.9951$, $^{d\#}p = 0.4712$, $^{e\#}p = 0.8833$, $^{f\#}p = 0.8617$, $^{g\#}p = 0.8615$.

Supplemental Table 3. Functional characterization of PW164 in HEK293 cells stably expressing Nav1.6 and transiently expressing FGF13-1a.

**Condition**	**Peak density**	**Activation**	**Steady state inactivation**	**Tau (τ)**
	pA/pF	mV	mV	ms
FGF13-1a-GFP + vehicle	−122.7 ± 12.1 (17)	−32.28 ± 1.7 (17)	−49.6 ± 1.9 (15)	1.26 ± 0.07 (17)
FGF13-1a-GFP + PW164	−102.7 ± 8.8 (16)$^{a\#ns}$	−29.2 ± 1.2 (16)$^{b\#ns}$	−50.8 ± 2.1 (14)$^{c\#ns}$	1.46 ± 0.04 (16)$^{d\#ns}$

**Long-term inactivation (% maximal Na^+ current)**			
**Condition**	2nd Pulse	3rd Pulse	4th Pulse
FGF13-1a-GFP + vehicle	−76.4 ± 4.8 (15)	−65.1 ± 6.6 (15)	−62.2 ± 6.9 (15)
FGF13-1a-GFP + PW164	−67.4 ± 4.1 (13)$^{e\#ns}$	−59.7 ± 4.1 (13)$^{f\#ns}$	−54.2 ± 4.9 (13)$^{g\#ns}$

Data are mean ± SEM; ns = not significant; Student t-test.

Student t-test was calculated against the following group:
$^{\#}$=FGF13.1A GFP + vehicle

$^{a\#}p = 0.2108$, $^{b\#}p = 0.1545$, $^{c\#}p = 0.6747$, $^{d\#}p = 0.2174$, $^{e\#}p = 0.1754$, $^{f\#}p = 0.5180$, $^{g\#}p = 0.3709$

Supplemental Table 4. Functional characterization of PW164 in HEK293 cells stably expressing Nav1.5.

Condition	Peak density	Activation	Steady state inactivation	Tau (τ)
	pA/pF	mV	mV	ms
GFP + vehicle	−305.7 ± 26.9 (12)	−41.05 ± 2.3 (12)	−82.92 ± 1.0 (12)	1.21 ± 0.09 (12)
GFP + PW164	−314.9 ± 17.9 (12)$^{ns\#}$	−38.11 ± 1.8 (12)$^{ns\#}$	−82.7 ± 1.5 (12)$^{ns\#}$	1.12 ± 0.07 (12)$^{ns\#}$
FGF13-GFP + vehicle	−206.9 ± 13.0 (10)$^{a\#}$	−37.66 ± 1.4 (13)$^{ns\#}$	−73.5 ± 1.3 (10)$^{c\#}$	0.89 ± 0.05 (10)$^{e\#}$
FGF13-GFP + PW164	−203.0 ± 15.6 (14)$^{b\#,ns\$}$	−37.38 ± 2.1 (17)$^{ns\$,\#}$	−75.9 ± 2.1 (10)$^{d\#,ns\$}$	0.90 ± 0.04 (10)$^{f\#,ns\$}$

Long-term inactivation (% maximal Na^+ current)

Condition	2nd Pulse	3rd Pulse	4th Pulse
GFP + vehicle	−90.65 ± 2.31 (12)	−88.28 ± 2.8 (12)	−86.37 ± 3.20 (12)
GFP + PW164	−91.7 ± 1.27 (12)$^{ns\#}$	−85.98 ± 2.18 (12)$^{ns\#}$	−84.62 ± 2.28 (12)$^{ns\#}$
FGF13-GFP + vehicle	−88.88 ± 1.56 (10)$^{ns\#}$	−84.81 ± 1.48 (10)$^{ns\#}$	−83.83 ± 1.44 (10)$^{ns\#}$
FGF13-GFP + PW164	−89.1 ± 1.71 (10)$^{ns\$}$	−86.58 ± 1.69 (10)$^{ns\$}$	−84.72 ± 1.83 (10)$^{ns\$}$

Data are mean ± SEM; ns = not significant; one way ANOVA Tukey's multiple comparisons test.
Overall one-way ANOVA: peak current density ($p<0.0001$, $F_{3,45}$); V½ activation ($p<0.0008$, $F_{3,48}$); V½ steady-state inactivation ($p<0.0001$, $F_{3,40}$); tau ($p<0.0130$, $F_{3,43}$); long-term inactivation ($p<0.0447$, 0.0469, 0.0290, $F_{3,42}$)

Tukey's multiple comparisons were calculated against the following groups:
$^{\#}$=Nav1.5-GFP + vehicle
$^{\$}$=FGF13-GFP + vehicle
$^{a\#}p = 0.0065$, $^{b\#}p = 0.0044$, $^{c\#}p = 0.0001$, $^{d\#}p = 0.0049$, $^{e\#}p = 0.0147$, $^{f\#}p = 0.0155$

Supplemental Table 5. Effect of PW164 on Na+ currents in human RealDRG™ neurons

Condition	Peak Density (pA/pF)	Activation (mV)	Steady-state inactivation (mV)	Tau (τ) (ms)
**+ Nav1.8 blocker A-803467**				
Naïve - vehicle	−78.9 ± 6.7 (14)	−29.18 ± 0.92 (13)	−72.07 ± 0.55 (12)	1.07 ± 0.07 (14)
Naïve - PW164	−84.1 ± 5.9 (9)$^{ns\#}$	−30.27 ± 0.75 (9)$^{ns\#}$	−75.11 ± 0.78 (9)$^{ns\#}$	0.98 ± 0.09 (9)$^{ns\#}$
Cap+ - vehicle	−113.9 ± 9.5 (12)$^{a\#}$	−33.18 ± 0.91 (12)$^{c\#}$	−78.95 ± 0.69 (12)$^{e\#}$	0.95 ± 0.05 (11)$^{ns\#}$
Cap+ - PW164	−71.9 ± 9.4 (10)$^{b\$}$	−27.51 ± 1.22 (10)$^{d\$}$	−74.71 ± 1.43 (9)$^{f\$}$	1.39 ± 0.09 (10)$^{g\$}$
Cap+ SB-366791 - vehicle	−82.3 ± 4.9 (9)	−32.3 ± 0.53 (9)	−71.75 ± 3.08 (9)	1.46 ± 0.12 (9)
Cap+ SB-366791 - PW164	−95.2 ± 9.9 (9)$^{ns\&}$	−30.7 ± 0.53 (9)$^{ns\&}$	−73.56 ± 1.33 (9)$^{ns\&}$	1.52 ± 0.11 (9)$^{ns\&}$
**+ Nav1.7 blocker ProTx-II**				
Naïve - vehicle	−72.52 ± 11.33 (9)	−26.70 ± 1.08 (9)	−59.44 ± 0.73 (9)	0.99 ± 0.08 (9)
Naïve - PW164	−76.99 ± 6.01 (10)$^{ns\#}$	−29.68 ± 1.17 (10)$^{ns\#}$	−61.23 ± 1.29 (10)$^{ns\#}$	0.87 ± 0.04 (10)$^{ns\#}$
Cap+ - vehicle	−70.75 ± 9.87 (10)$^{ns\#}$	−29.59 ± 1.42 (10)$^{ns\#}$	−61.18 ± 1.53 (10)$^{ns\#}$	1.05 ± 0.05 (10)$^{ns\#}$
Cap+ - PW164	−80.16 ± 8.05 (9)$^{ns\$}$	−29.15 ± 0.86 (9)$^{ns\$}$	−61.95 ± 1.29 (9)$^{ns\$}$	0.98 ± 0.08 (9)$^{ns\$}$

Condition	2nd Pulse	3rd Pulse	4th Pulse
**+ Nav1.8 blocker A-803467 Long-term inactivation (% maximal Na^+ current)**			
Naïve + vehicle	−73.14 ± 4.18 (12)	−68.17 ± 2.73 (12)	−64.12 ± 2.64 (12)
Naïve + PW164	−74.84 ± 5.46 (11)$^{ns\#}$	−67.02 ± 3.43 (11)$^{ns\#}$	−65.63 ± 4.22 (11)$^{ns\#}$
Cap+ vehicle	−57.89 ± 3.59 (12)$^{h\#}$	−54.70 ± 3.25 (12)$^{j\#}$	−53.68 ± 2.63 (12)$^{l\#}$
Cap+ PW164	−45.59 ± 3.33 (10)$^{i\#}$	−38.18 ± 2.61 (10)$^{k\#}$	−32.17 ± 4.29 (10)$^{m\#}$
Cap+ SB-366791 vehicle	−59.29 ± 6.06 (9)	−53.13 ± 5.68 (9)	−49.46 ± 5.97 (9)
Cap+ SB-366791 PW164	−54.94 ± 3.35 (9)$^{ns\&}$	−47.15 ± 2.81 (9)$^{ns\&}$	−45.57 ± 4.0 (9)$^{ns\&}$
**+ Nav1.7 blocker ProTx-II Long-term inactivation (% maximal Na^+ current)**			
Naïve + vehicle		−116.62 ± 4.83 (9)	−122.51 ± 4.75 (9)
Naïve + PW164	−105.72 ± 2.72 (10)$^{ns\#}$	−110.76 ± 2.72 (10)$^{ns\#}$	−113.94 ± 3.54 (10)$^{ns\#}$
Cap+ vehicle	−118.72 ± 5.15 (10)$^{ns\#}$	−125.70 ± 7.32 (10)$^{ns\#}$	−130.64 ± 8.60 (10)$^{ns\#}$
Cap+ PW164	−118.43 ± 3.81 (9)$^{ns\$}$	−120.42 ± 4.69 (9)$^{ns\$}$	−120.42 ± 4.69 (9)$^{ns\$}$

data are mean ± SEM; ns = not significant; one way ANOVA Tukey's multiple comparison test.
Overall one-way ANOVA for Na+ currents in the presence of the Nav1.8 blocker A-803467: peak current density ($p<0.0034$, $F_{3,41}$); V½ activation ($p<0.0016$, $F_{3,40}$); V½ steady-state inactivation ($p<0.0001$, $F_{3,38}$); tau ($p<0.0019$, $F_{3,40}$); long-term inactivation ($p<0.0001$, $F_{3,41}$).

Overall one-way ANOVA for Na+ currents in the presence of the Nav1.7 blocker ProTx-II: peak current density ($p<0.8784$, $F_{3,34}$); V½ activation ($p<0.1654$, $F_{3,34}$); V½ steady-state inactivation ($p<0.5368$, $F_{3,34}$); tau ($p<0.3324$, $F_{3,34}$); long-term inactivation ($p<0.0323$, 0.1960, 0.2420, $F_{3,34}$).
Tukey's multiple comparisons were calculated against the following groups:
$^{\#}$=Naïve – vehicle, $^{\$}$=Cap+ - vehicle, $^{\&}$=Cap+ SB-366791- vehicle
$^{a\#}p$= 0.0021, $^{b\$}p$= 0.0003, $^{c\#}p$= 0.0186, $^{d\$}p$ = 0.0011, $^{e\#}p$= 0.0001, $^{f\$}p$= 0.0075, $^{g\#}p$= 0.0021, $^{h\#}p$= 0.0371, $^{i\#}p$= 0.0481, $^{j\#}p$ = 0.0127, $^{k\#}p$= 0.0183, $^{l\#}p$= 0.0034, $^{m\#}p$= 0.0268.

Supplemental Table 6. Effect of PW164 on Na+ currents in human RealDRG™ neurons expressing pAAV-shCTRL-GFP, pAAV-shFGF13-GFP or pAAV-FGF13-GFP with (Cap+) or without (Cap-) capsaicin treatment.

+ Nav1.8 blocker A-803467				
**Condition**	**Peak Density (pA/pF)**	**Activation (mV)**	**Steady-State inactivation (mV)**	**Tau (τ) (ms)**
Cap- Control + vehicle	−107.33 ± 9.71 (11)	−25.21 ± 0.85 (11)	−59.38 ± 1.64 (11)	1.03 ± 0.05 (11)
Cap- Control + PW164	−111.31 ± 11.73 (11)[ns&]	−25.74 ± 1.14 (11)[ns&]	−60.96±1.53 (11)[ns&]	1.18 ± 0.09 (11)[ns&]
Cap + Control + vehicle	−199.63 ± 24.08 (12)[a&]	−29.96 ± 0.86 (11)	−66.08 ± 1.77 (11)[e&]	0.97 ± 0.09 (11)
Cap + Control + PW164	−92.79 ± 7.96 (12)[b#]	−21.92 ± 0.87 (12)[d#]	−58.11 ± 1.04 (12)[f#]	1.42 ± 0.09 (12)[g#]
Cap- ShFGF13 + vehicle	−90.61 ± 6.89 (10)	−26.98 ± 1.12 (10)	−61.58 ± 1.64 (10)	1.25 ± 0.09 (10)
Cap- ShFGF13 + PW164	−96.02 ± 8.94 (10)[ns@]	−29.77 ± 1.74 (10)[ns@]	−64.99 ± 1.30 (10)[ns@]	0.98 ± 0.09 (10)[ns@]
Cap+ ShFGF13 + vehicle	−73.44 ± 3.54 (8)[c&#]	−34.07 ± 1.14 (8)	−68.16 ± 1.27 (8)	1.30 ± 0.12 (8)
Cap+ ShFGF13+PW164	−76.49 ± 4.86 (10)	−32.72 ± 1.59 (10)	−67.68 ± 1.53 (10)	1.38 ± 0.10 (10)
Cap+ AAV-GFP vehicle	−274.72 ± 21.4 (7)	−32.94 ± 1.25 (7)	−66.54 ± 0.99 (7)	1.06 ± 0.05 (7)
Cap+ AAV-GFP PW164	−87.62 ± 9.5 (10)[h€]	−28.39 ± 0.59 (10)[k€]	−75.15 ± 1.69 (10)[m€]	2.0 ± 0.32 (10)[n€]
Cap+ AAV-FGF13 vehicle	−376.25 ± 19.2 (8)[i€]	−31.8 ± 0.75 (8)	−69.44 ± 1.19 (8)	0.96 ± 0.04 (8)
Cap+ AAV-FGF13 PW164	−91.7 ± 10.3 (8)[j£]	−27.8 ± 0.81 (8)[l£]	−73.36 ± 1.4 (8)[ns£]	1.63 ± 0.22 (8)[ns£]

+ Nav1.8 blocker A-803467<br>Long-term inactivation (% maximal Na⁺ current)			
**Condition**	2nd Pulse	3rd Pulse	4th Pulse
Cap- Control + vehicle	−86.54 ± 1.7 (11)	−83.33 ± 2.13 (11)	−80.84 ± 2.42 (11)
Cap- Control + PW164	−86.72 ± 2.2 (11)[ns&]	−82.89 ± 3.3 (11)[ns&]	−81.6 ± 3.6 (11)[ns&]
Cap + Control + vehicle	−67.86 ± 5.26 (11)[o&]	−62.17 ± 5.42 (11)[q&]	−58.74 ± 5.79 (11)[s&]
Cap + Control + PW164	−59.86 ± 2.37 (12)	−49.84 ± 2.93 (12)	−46.98 ± 3.3 (12)
Cap- ShFGF13 + vehicle	−78.89 ± 3.77 (9)	−74.54 ± 4.36 (9)	−72.59 ± 4.39 (9)
Cap- ShFGF13 + PW164	−82.82 ± 2.85 (10)[ns@]	−81.52 ± 3.86 (10)[ns@]	−81.16 ± 4.06 (10)[ns@]
Cap+ ShFGF13 + vehicle	−79.35 ± 4.8 (10)	−75.1 ± 5.2 (10)	−71.34 ± 5.35 (10)
Cap+ ShFGF13 + PW164	−74.3 ± 3.96 (10)[p%,ns$]	−68.4 ± 5.4 (10)[r%,ns$]	−67.2 ± 5.2 (10)[t%,ns$]
Cap+ GFP vehicle	−58.17 ± 2.7 (7)	−56.93 ± 3.3 (7)	−56.62 ± 2.8 (7)
Cap+ GFP PW164	−53.56 ± 4.9 (10)[ns€]	−54.96 ± 3.6 (10)[ns€]	−54.22 ± 4.3 (10)[ns€]
Cap+ hFGF13 vehicle	−42.17 ± 3.6 (8)[u€]	−38.7 ± 2.0 (8)[v€]	−31.19 ± 3.2 (8)[x€]
Cap+ hFGF13 PW164	−55.4 ± 2.7 (8)[ns£]	−52.0 ± 3.7 (8)[w£]	−45.4 ± 3.1 (8)[y£]

data are mean ± SEM; ns = not significant; one way ANOVA Tukey's multiple comparison test. Overall one-way ANOVA: peak current density ($p<0.0001$, $F_{7,76}$); V½ activation ($p<0.0001$, $F_{7,76}$); V½ steady-state inactivation ($p<0.0001$, $F_{7,75}$); tau ($p<0.0018$, $F_{7,76}$); long-term inactivation ($p<0.0001$, $F_{7,74}$).

Tukey's multiple comparisons were calculated against the following groups: [&]=Cap- Control + vehicle; [#]=Cap+ Control + vehicle; [%]=Cap+ Control + PW164; [@]=Cap- ShFGF13 + vehicle; [$]=Cap+ ShFGF13 + vehicle; [€]=Cap+ GFP + vehicle; [£]=Cap+ hFGF13 vehicle.

[a&]*p* = 0.0001, [b#]*p* = 0.0001, [c&#]*p* = 0.0001, [d#]*p*= 0.0013, [e&]*p* = 0.0344, [f#]*p* = 0.0041, [g#]*p* = 0.0168, [h€]*p*=0.0001, [i£]*p*=0.0001, [j€]*p*=0.0001, [k€]*p*=0.0238, [l£]*p*=0.0282, [m€]*p*=0.0048, [n€]*p*=0.0109, [o&]*p*= 0.0048, [p%]*p* = 0.0458, [q&]*p*= 0.0046, [r%]*p* = 0.0054, [s&]*p* = 0.0040, [t%]*p* = 0.0177, [u€]*p*=0.0422, [v€]*p*=0.0053, [w£]*p*=0.0459, [x€]*p*=0.0003, [y£]*p*=0.0469.

Supplemental Table 7. Functional characterization of ZL192 in HEK293 cells stably expressing Nav1.7.

Condition	Peak density	Activation	Steady-state inactivation	Tau (τ)
	pA/pF	mV	mV	ms
GFP + vehicle	−61.6 ± 5.1 (12)	−26.0 ± 0.3 (12)	−82.3 ± 1.3 (10)	1.44 ± 0.15 (12)
GFP + ZL192	−73.5 ± 8.1 (11)[ns#]	−25.7 ± 0.8 (11)[ns#]	−82.8 ± 1.1 (9)[ns#]	1.3 ± 0.1 (10)[ns#]
FGF13-GFP + vehicle	−97.98 ± 12.5 (11)[a#]	−22.3 ± 1.3 (12)[d#]	−74.5 ± 2.2 (12)[g#]	1.06 ± 0.07 (11)[ns]
FGF13-GFP + ZL192	−139.7 ± 12.3 10)[b$,c€]	−26.0 ± 0.8 (10)[e$,f€]	−66.7 ± 2.6 (10)[h$,i€]	1.2 ± 0.06 (9)[ns]

LTI (% maximal Na^+ current)			
**Condition**	2nd Pulse	3rd Pulse	4th Pulse
GFP + vehicle	−63.72 ± 4.30 (11)	−59.34 ± 5.34 (11)	−61.99 ± 5.63 (11)
GFP + ZL192	−67.94 ± 3.77 (9)[ns#]	−62.35 ± 57.35 (9)[ns#]	−60.93 ± 3.90 (9)[ns#]
FGF13-GFP + vehicle	−89.11 ± 2.82 (12)[j#]	−84.66 ± 2.55 (12)[l#]	−82.64 ± 3.01 (12)[n#]
FGF13-GFP + ZL192	−69.72 ± 5.21 (10)[k$]	−60.33 ± 5.60 (10)[m$]	−59.34 ± 4.68 (10)[o$]

data are mean ± SEM; ns = not significant; one way ANOVA Tukey's multiple comparisons test.

Overall one-way ANOVA: peak current density ($p<0.0001$, $F_{3.40}$); V½ activation ($p<0.0102$, $F_{3,41}$); V½ steady-state inactivation ($p<0.0001$, $F_{3,37}$); tau ($p<0.1073$, $F_{3,38}$); long-term inactivation ($p<0.0001$, $F_{3,38}$).

Tukey's multiple comparisons were calculated against the following groups:

[#]=GFP + vehicle

[$]=FGF13-GFP + vehicle

[€]=GFP + ZL192

[a#]*p* = 0.0465, [b$]*p* = 0.0247, [c€]*p* = 0.0002, [d#]*p* = 0.0205, [e$]*p* = 0.0457, [f€]*p* = 0.0301, [g#]*p*= 0.0368, [h$]*p* = 0.0331, [i€]*p* = 0.0001, [j#]*p* = 0.0003, [k#]*p*= 0.0500, [l#]*p*= 0.0014, [m$]*p* = 0.0001, [n#]*p*= 0.0486, [o$]*p* = 0.0002.

Supplemental Table 8. Effect of ZL192 on Na+ currents in RealDRGs neurons naïve or expressing pAAV-GFP or pAAV-shFGF13-GFP.

Condition	Peak Density (pA/pF)	Activation (mV)	Steady-State inactivation (mV)	Tau (τ) (ms)
**+ Nav1.8 blocker A-803467**				
Naive + vehicle	−97 ± 11.09 (17)	−25.31 ± 0.73 (10)	−61.03 ± 0.93 (10)	1.07 ± 0.07 (14)
Naive + ZL192	−181.06 ± 13.74 (10)[a&]	−29.92 ± 0.79 (14)[c&]	−72.03 ± 0.94 (12)[e&]	1.31 ± 0.06 (10)[ns$]
Control + vehicle	−102.26 ± 10.19 (10)	−24.31 ± 0.87 (10)	−61.44 ± 2.66 (10)	1.03 ± 0.05 (12)
Control + ZL192	−189.79 ± 15.05 (10)[b#]	−29.64 ± 0.92 (16)[d#]	−70.68 ± 1.76 (15)[f#]	1.09 ± 0.05 (10) [ns#]
Sh + vehicle	−90.61 ± 6.89 (10)	−26.98 ± 1.12 (10)	−61.58 ± 1.64 (10)	1.25 ± 0.09 (10)
Sh + ZL192	−77.34 ± 6.83 (10)[ns$]	−22.84 ± 1.28 (10)[ns$]	−61.77 ± 2.40 10)[ns$]	1.23 ± 0.05 (10)[ns$]
AAV-GFP	134.7 ± 8.38 (11)	−25.76 ± 1.0 (11)	−68.7 ± 1.2 (11)	1.1 ± 0.07 (11)
AAV-FGF13 + vehicle	−289.8 ± 16.08 (11)	−29.37 ± 0.9 (11)	−70.6 ± 1.9 (11)	1.06 ± 0.06 (11)
AAV-FGF13 + PW164	−114.3 ± 5.6 (10)[g%]	−24.3 ± 0.8 (10)[h%]	−75.65 ± 1.6 (10)[i%]	1.65 ± 0.14 (10)[j%]
**+ Nav1.7 blocker ProTx-II**				
Naive + vehicle	−94.52 ± 8.63 (12)	−27.62 ± 1.11 (12)	−61.59 ± 0.83 (12)	0.99 ± 0.05 (12)
Naive + ZL192	−96.53 ± 4.85 (12)[ns&]	−27.64 ± 0.93 (12)[ns&]	−59.34 ± 1.61 (12)[ns&]	1.33 ± 0.06 (12)[ns&]
Control + vehicle	−90.59 ± 4.6 (8)	−26.66 ± 2.24 (8)	−58.82 ± 2.53 (8)	1.23 ± 0.08 (8)
Control + ZL192	−101.87 ± 5.35 (11)[ns#]	−30.93 ± 0.89 (11)[ns#]	−53.15 ± 0.91 (11)[ns#]	1.0 ± 0.04 (11)[ns#]
Sh + vehicle	−89.32 ± 5.14 (10)	−27.35 ± 1.41 (10)	−61.81 ± 1.85 (10)	1.43 ± 0.09 (10)
Sh + ZL192	−99.91 ± 8.28 (12)[ns$]	−28.74 ± 1.12 (11)[ns$]	−62.97 ± 1.64 (11)[ns$]	1.27 ± 0.08 (11)[ns$]
**+ Nav1.8 blocker A-803467<br>Long-term inactivation (% maximal Na^+ current)**				
**Condition**		2nd Pulse	3rd Pulse	4th Pulse
Naïve + vehicle		−63.14 ± 4.18 (12)	−55.17 ± 2.73 (12)	−54.12 ± 2.64 (12)
Naïve + ZL192		−90.95 ± 1.82 (10)[k#]	−88.17 ± 2.02 (10)[m#]	−86.7 ± 1.7 (10)[o#]
Control + vehicle		−66.62 ± 6.73 (10)	−61.88 ± 7.08 (10)	−60.25 ± 7.14 (10)
Control + ZL192		−90.69 ± 2.00 (11)[l&]	−89.18 ± 2.37 (11)[n&]	−87.9 ± 2.75 (11)[p&]
Sh + vehicle		−78.89 ± 3.77 (9)	−74.54 ± 4.36 (9)	−72.59 ± 4.39 (9)
Sh + Zl192		−84.46 ± 3.81 (10)[ns$]	−82.09 ± 3.89 (10)[ns$]	−80.59 ± 4.08 (10)[ns$]
AAV GFP		−78.2 ± 3.6 (11)	−75.22 ± 2.8 (11)	−72.4 ± 2.6 (11)
AAV FGF13 + vehicle		−78.3 ± 4.1 (11)	−76.36 ± 4.05 (11)	−75.9 ± 4.5 (11)
AAV FGF13 + PW64		−68.4 ± 7.1 (10)[ns%]	−61.9 ± 7.5 (8)[ns%]	−58.5 ± 8.3 (10)[ns%]
**+ Nav1.7 blocker ProTx-II<br>Long-term inactivation (% maximal Na^+ current)**				
**Condition**		2nd Pulse	3rd Pulse	4th Pulse
Naïve + vehicle		−98.16 ± 1.23 (12)	−102.62 ± 3.15 (12)	−104.28 ± 3.47 (12)
Naïve + ZL192		−99.26 ± 0.91 (12)[ns&]	−100.38 ± 1.08 (12)[ns&]	−100.45 ± 1.3 (12)[ns&]
Control + vehicle		−97.13 ± 2.6 (9)	−98.23 ± 2.93 (9)	−97.53 ± 3.21 (9)
Control + ZL192		−98.61 ± 0.74 (11)[ns#]	−99.17 ± 0.84 (11)[ns#]	−98.95 ± 1.01 (11)[ns#]
Sh + vehicle		−91.96 ± 2.48 (10)	−90.76 ± 2.69 (10)	−89.82 ± 3.06 (10)
Sh + Zl192		−96.65 ± 1.81 (11)	−97.25 ± 2.18 (11)	−99.23 ± 3.28 (11)

data are mean ± SEM; ns = nonsignificant; one way ANOVA Tukey's multiple comparison testOverall one-way ANOVA for Na+ currents in the presence of the Nav1.8 blocker A-803467: peak current density ($p<0.0001$, $F_{6,78}$); V½ activation ($p<0.0001$, $F_{6,75}$); V½ steady-state inactivation ($p<0.0001$, $F_{6,72}$); tau ($p<0.0030$, $F_{6,70}$); long-term inactivation ($p<0.0001$, $F_{5,56}$). Tukey's multiple comparisons were calculated against the following groups:[&]=Naive + vehicle; [#]=Control + vehicle; [$]=Sh + vehicle; [%]=hFGF13 + vehicle[a&]$P = 0.0122$, [b#]$P = 0.0107$, [c&]$P = 0.0174$, [d#]$P = 0.0023$, [e&]$P = 0.0005$, [f#]$P = 0.0030$, [g%]$P = 0.0001$, [h%]$P = 0.0057$, [i%]$P = 0.0405$, [j%]$P = 0.0415$, [k#]$P = 0.0001$, [l&]$P = 0.0011$, [m#]$P = 0.0001$, [n&]$P = 0.0001$, [o#]$P = 0.0001$, [p&]$P = 0.0001$.

Supplemental Table 9. Pharmacokinetics study of test compound PW164 on C57BL/6 mice

Animal	No. of pts used for t1/2	T1/2 (h)	Tmax	Cmax (ng/ml)	AUClast (h*ng/ml)	AUCInf (h*ng/ml)	AUC Extr (%)	MRTInf (h)	AUClast/D (h*kg*ng/ml/mg)	F (%)	Last time point for AUClast (h)	Time points for T1/2 (h)	Rsq
**Mouse#19**	3	4.43	0.0833	25.6	71.0	100	29.1	6.36	4.73	NA	8	2,4,8	0.995
**Mouse#20**	4	5.16	0.0833	19.1	59.2	90.0	34.2	7.32	3.95	NA	8	1,2,4,8	0.989
**Mouse#21**	3	1.63	0.0833	13.4	41.3	43.4	4.86	2.78	2.75	NA	8	2,4,8	0.960
**N**	3	3	3	3	3	3	3	3	3	0			
**Mean**		3.74	0.0833	19.4	57.2	77.8	22.7	5.49	3.81	NA			
**SD**		1.86	0.000	6.1	15.0	30.3	15.7	2.39	1.00	NA			
**CV (%)**		49.8	0.0	31.5	26.2	38.9	69.0	43.6	26.2	NA			

Temperature and humidity, 2024 ºC, 40-70%, intradermal administration (i.d.), formulation condition: solution, vehicle: DMSO.
Experiments were conducted by BioDuro (Irvine, CA).

Supplemental Table 10. Efficacy study of test compound PW164 on C57BL/6 mice

Group	No.	Time after dosing									Body weight (g)	
		0h			2h			8h			Pre-dose	Terminal
		Systolic	Diastolic	MAP	Systolic	Diastolic	MAP	Systolic	Diastolic	MAP		
**G1-PW164 (15 mpk)**	85	120	98	105	107	75	86	114	78	89	25.7	24.9
	87	101	72	81	100	64	76	121	94	103	27.0	26.8
	88	108	63	78	103	68	79	123	81	95	24.4	23.9
	89	118	95	103	125	76	92	120	74	89	24.4	23.8
	91	121	77	92	111	85	93	117	76	89	25.0	24.5
**Mean**		**114**	**81**	**92**	**109**	**74**	**85**	**119**	**81**	**93**	**25.3**	**24.8**
**SEM**		**3.8**	**6.8**	**5.4**	**4.3**	**3.6**	**3.4**	**1.6**	**3.7**	**2.8**	**0.5**	**0.5**

Temperature and humidity, 2024 ºC, 40-70%, intradermal administration (i.d.), formulation condition: solution, vehicle: DMSO.
Experiments were conducted by BioDuro (Irvine, CA).

Supplemental Table 11. Rare genetic variants identified in the clinical population.

Gene	Transcript	g.pos	dbSNP ID	c.pos	p.pos	GnomAD	DPN (n=230)	DPLN (n=317)	CP (n=1425)	HC (n=216)	Grantham distance	Pathogenicity Class (ACMG standards)	ClinVar ID	ClinVar interpretation
SCN9A	NM_001365536.1	2:167055682_G/A	rs752580340	c.5467C>T	Leu1823Phe	0.00079%	·	·	1 (0.7%)	·	22	PM2, PP3	846199	VUS
		2:167055658_C/A	rs200196731	c.5491G>T	Val1831Phe	0.0014%	·	1 (0.3%)	·	·	50	PM2, PP3	946549	VUS
		2:167055606_C/T	·	c.5543G>A	Arg1848His	·	·	·	1 (0.7%)	·	29	PM2, PP3	892606	VUS
		2:167055370_G/A	rs111558968	c.5779C>T	Leu1927Phe	0.1%	1 (0.4%)	·	2 (1.4%)	·	22	BP4, BP6	196020	LB
		2:167055200_G/C	rs201640210	c.5949C>G	Ser1983Arg	0.003%	·	·	1 (0.7%)	·	110	BP4, BP6	·	·
FGF13	NM_004114.5	X:137717737_C/T	rs1477376621	c.482G>A	Arg161His	0.0005%	·	·	1 (0.7%)	·	29	PM1, PM2, PP3, BP4	·	·

g.pos, genomic position; c.pos coding position; p.pos, protein position; GnomAD, alternate allele frequency from gnomAD v2.1.1 (GRCh37) including Exomes and Genomes, Total (average among all ancestry groups); CP, chronic pain; DPN, Diabetic Painful Neuropathy; DPLN, Diabetic Painless Neuropathy; HC, Healthy Controls. Grantham distance is a prediction of the effect of substitutions between amino acids based on physiochemical properties. ACMG, American College of Medical Genetics and Genomics classification; VUS, variant of uncertain clinical significance; LB, likely benign
